# Quantification of Binding of Small Molecules to Native Kinases by Flow Cytometry Reveals Divergence from Biochemical Affinities

**DOI:** 10.1021/jacs.6c06577

**Published:** 2026-06-23

**Authors:** Lillian M. Cool, Jogendra Pawar, Sonam Sonam, Smita Kumari, Serena Li Zhao, Xiaojun Hu, Zhihong Lin, Meng Wu, Shuiying Hu, Blake R. Peterson

**Affiliations:** †Division of Medicinal Chemistry and Pharmacognosy, ‡Division of Pharmaceutics and Pharmacology, The Ohio State University, College of Pharmacy, 500 W. 12th Ave., Columbus, Ohio 43210, United States; § The Ohio State University Comprehensive Cancer Center—Arthur G. James Cancer Hospital and Richard J. Solove Research Institute, 460 W. 10th Ave., Columbus, Ohio 43210, United States

## Abstract

Precise measurements of small molecule–protein interactions are critical for drug discovery. However, most biochemical profiling platforms measure binding using recombinant kinase domains or proteins in cell lysates, which can miss conformational regulation present in intact living cells. Here, we used flow cytometry–based fluorescent probe cellular binding assays (FPCBA) to demonstrate that the anticancer drug dasatinib binds native, untagged ABL1 kinase with 3–6-fold higher affinity than NanoLuc- or mVenus-tagged constructs in living cells. We further used this method for in-cell profiling of 25 native kinases, revealing conformational regulatory mechanisms, including SRC autoinhibition and membrane-dependent conformational states of DDR1, DDR2, and EPHA4 that are absent or attenuated in biochemical assays. For these studies, coumarin-dasatinib probes spanning a range of fluorophore acidity (p*K*_a_ 4.1–7.3) were optimized for intracellular target engagement. To enhance sensitivity of detection, we found that uptake of acidic probes can be promoted by expression of the organic anion transporter OATP1B3. Quantitative flow cytometry with NIST-standardized beads established that intracellular concentrations of an intermediate-acidity 6FC-dasatinib probe approximated extracellular concentrations in HEK293T cells at equilibrium. Cellular *K_i_* values of dasatinib and imatinib for 25 kinases by FPCBA were broadly concordant with kinobead LC/MS measurements in cancer cell lysates but diverged substantially from recombinant KINOMEscan values, with divergences attributable to competition with ATP, autoinhibition, and membrane-dependent conformational states in living cells. FPCBA enables profiling of native protein–small molecule interactions in a physiologically relevant cellular context.

## Graphical abstract

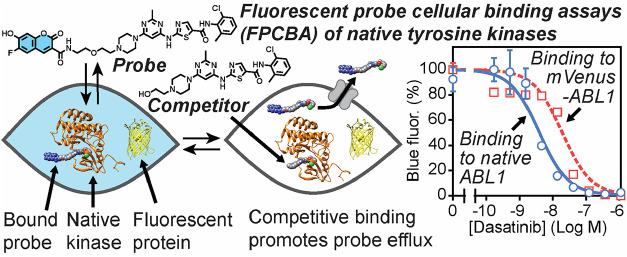

## Introduction

Cellular target engagement assays are essential for drug discovery and development.^1–5^ They provide direct evidence of small molecule–protein interactions in a physiologically relevant context. Unlike biochemical assays with purified or recombinant proteins, cell-based assays can capture the influence of post-translational modifications, protein–protein interactions, conformational regulation, transporters, and subcellular localization on drug binding and efficacy.^6–8^ Some widely used cellular target engagement platforms such as NanoBRET^9–12^ require fusion of reporter tags to target proteins (Figure 1), which can in principle perturb native protein regulation and alter apparent drug affinity. Whether and to what extent these perturbations affect measurements of small molecule-protein interactions across kinase families has remained largely unexplored.

**Figure 1:**
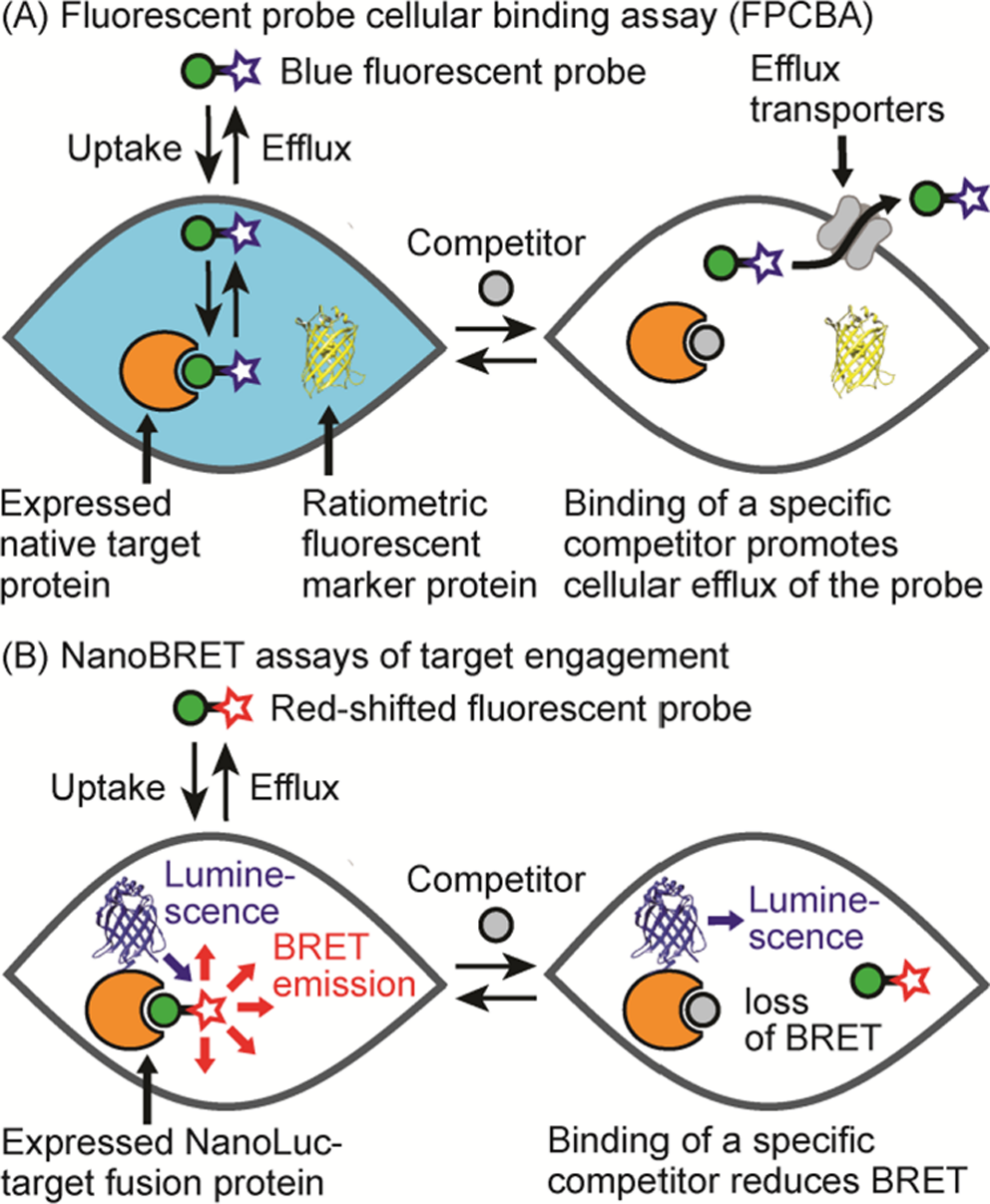
Comparison of FPCBA and NanoBRET for quantifying engagement of protein targets by small molecules. In both methods, cells are typically transiently transfected with genes encoding target protein(s). (A) In FPCBA, overexpression of the target promotes cellular uptake of a blue-fluorescent probe through specific binding. Binding at equilibrium is quantified by flow cytometry by correlating expression of the target with probe fluorescence, using a coexpressed fluorescent reporter (e.g., mVenus) encoded on a shared mRNA using an IRES vector. (B) In NanoBRET, the target is expressed as a nanoluciferase fusion protein. Binding of a red-shifted probe enables bioluminescence resonance energy transfer upon addition of a substrate of NanoLuc, producing red-shifted emission. Both methods support competition assays to quantify interactions between the expressed protein and unlabeled ligands.

We previously reported^13^ the Fluorescent Probe Cellular Binding Assay (FPCBA), a flow cytometry–based method for quantifying small molecule–protein interactions in living cells (Figure 1). Analogous to fluorescence polarization competitive binding assays,^14^ FPCBA enables quantitative measurements of ligand–protein interactions in a homogeneous (no-wash) format by correlating cellular uptake of fluorescent probes with overexpression of target proteins using an internal ribosomal entry site (IRES)^15^ vector that coexpresses the target with a spectrally orthogonal fluorescent reporter protein. This approach preserves endogenous protein regulation and allows measurement of apparent equilibrium dissociation constants (*K_i_*) directly in living cells. Whereas our initial report established proof-of-concept using allosteric probes of protein kinase C (PKC), here we extend FPCBA to orthosteric binding sites of human tyrosine kinases and introduce three platform advances: systematic coumarin probe engineering for orthosteric target engagement, transporter-assisted probe uptake using cells expressing the organic anion transporting polypeptide OATP1B3 (encoded by *SLCO1B3*),^16,17^ and quantitative flow cytometry calibration for direct measurement of intracellular probe concentrations. Based on our previous studies^18^ of the OATP1B3 substrate PB-Gly-Taxol,^19^ we hypothesized that coumarin-derived dasatinib probes with systematic variations in acidity would serve as OATP1B3 substrates, enabling enhanced intracellular accumulation and improved FPCBA sensitivity. We profiled cellular affinities of imatinib and dasatinib (Figure 2), ATP-site inhibitors of the oncogenic kinase Bcr-Abl in chronic myelogenous leukemia (CML)^20,21^ that bind multiple tyrosine kinases,^22–24^ across a panel of 25 native full-length human kinases and compared these values with published biochemical affinities from KINOMEscan^25^ and kinobeads.^24^ Comparison with NanoBRET and with matched N-terminally tagged ABL1 fusion proteins revealed that genetic fusions alter the apparent affinity of kinase inhibitors in an inhibitor-dependent manner, and profiling of 25 kinases revealed both broad concordance and informative divergences from biochemical measurements attributable to native conformational regulation in intact live cells.

**Figure 2:**
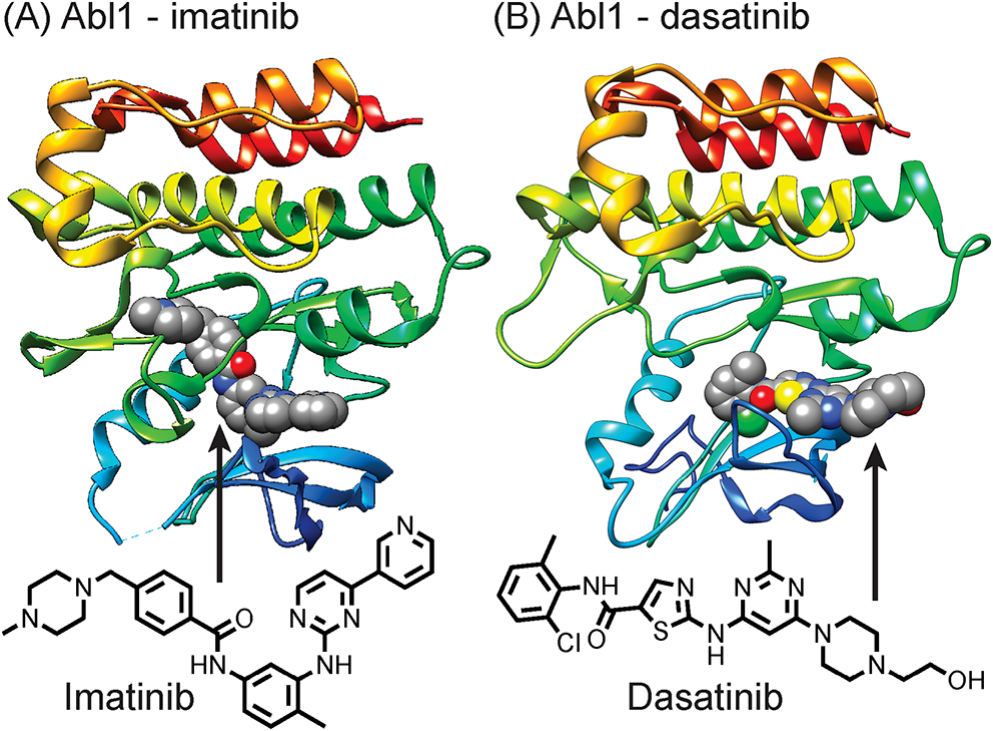
X-ray crystal structures of the kinase domain of ABL1 (ribbon models) bound to the anticancer drugs imatinib (A, PDB 2HYY,^26^ inactive DFG-out conformation) and dasatinib (B, PDB 2GQG,^27^ active DFG-in conformation) shown as sphere models.

## Results

### Design and Synthesis of Fluorescent Molecular Probes

As shown in Figure 3, we developed a series of fluorescent molecular probes based on Pacific Blue (PB),^28^ 6-fluoro-7-hydroxycoumarin-3-carboxylic acid (6FC),^29^ and 7-hydroxycoumarin-3-carboxylic acid (7HC or 7OHCCA).^28^ These coumarin fluorophores were linked either to the kinase inhibitor dasatinib (compounds **1**–**3**) or to a ligand of the HaloTag binding protein (compounds **4**–**6**). HaloTag ligands bind covalently to the Asp106 residue of this protein, allowing these fluorophores to be immobilized on the protein surface. Coumarin 30 (**C30**) was used as a reference compound for optical spectroscopy, and PB-Gly-Taxol (**7**),^19^ a microtubule-binding substrate of OATP1B3,^18^ was used to isolate a stable cell line expressing OATP1B3 derived from HEK293T. As reporter elements, these coumarin fluorophores differ systematically in chemical and biological properties,^29^ including brightness, polarity, and sensitivity to cellular transporters. These differences are largely related to their altered p*K*_a_ values,^29^ where Pacific Blue amides are the most acidic (p*K*_a_ = 4.1), 6FC amides are intermediate acidity (p*K*_a_ = 6.5), and 7HC amides are the least acidic (p*K*_a_ = 7.3). These differences in p*K*_a_ affect both the polarity of these probes (*c* Log *D*_7.4_ (**1**) = 3.0; *c* Log *D*_7.4_ (**2**) = 3.2; *c* Log *D*_7.4_ (**3**) = 3.9, calculated with ChemAxon Marvin 24.3.0) and their fluorescence brightness. For comparison, we also evaluated BODIPY-Dasatinib (**8**), a red fluorescent BODIPY 576/589-dasatinib conjugate commercially available as Tracer K4 (Promega) and previously reported as Energy Transfer Probe 6 by Vasta et al.^9^ (confirmed here by HRMS; see Supporting Information), in NanoBRET-based kinase binding studies. The synthesis of the coumarin-derived probes **1**–**6** is described in the Supporting Information (Figure S1). Other fluorescent derivatives of dasatinib have also been reported.^30^

**Figure 3:**
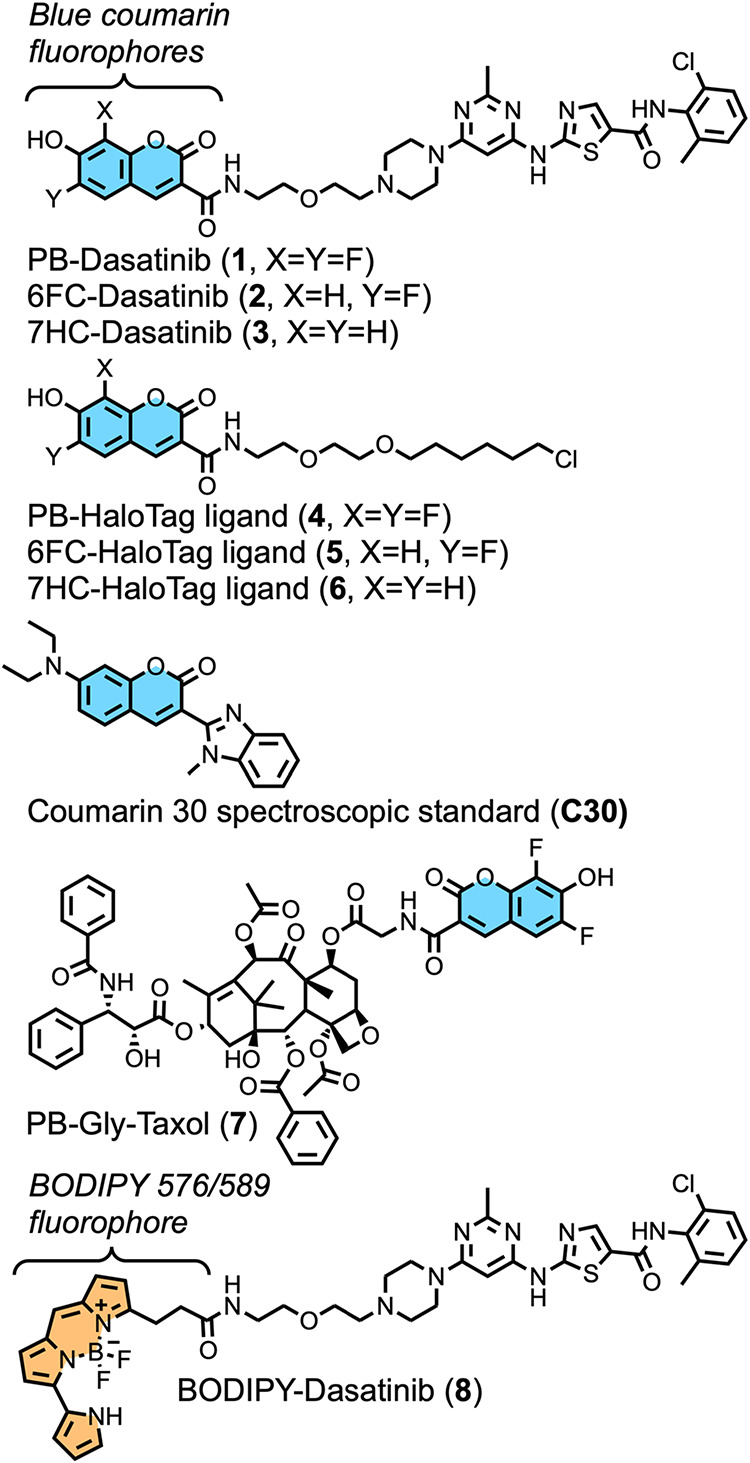
Structures of fluorescent probes. Coumarin derivatives of dasatinib (**1**–**3**) were designed to bind tyrosine kinases and coumarin-chloroalkane derivatives (**4**–**6**) were designed as covalent ligands of HaloTag binding protein. Coumarin 30 (**C30**) was used as a spectroscopic standard, PB-Gly-Taxol (**7**)^19^ was used as a fluorescent substrate to isolate a derivative of HEK293T cells overexpressing OATP1B3, and BODIPY-Dasatinib (**8**)^9^ was used for comparisons of NanoBRET with FPCBA.

### Analysis of the Brightness of Probes Relative to Coumarin 30 to Estimate Intracellular Concentrations of Probes and Target Proteins

Quantitative flow cytometry (qFlow) is an extension of conventional flow cytometry in which the intensity of cellular fluorescence is converted into absolute numbers of fluorescent molecules.^31^ To achieve this, calibration beads bearing defined numbers of fluorophores per bead are used to create a standard curve that allows fluorophore concentrations per cell to be determined. We hypothesized that this could benefit studies of intracellular engagement of target proteins by FPCBA because actions of efflux and influx transporters, among other mechanisms, can profoundly affect intracellular probe concentrations. Through measurement of *B*_max_ values in binding assays, these values were used to estimate the concentrations of expressed intracellular target proteins for analysis of small molecule-protein interactions.

To measure intracellular probe concentrations, we sought to account for the exceptionally protein-rich environment of the cytoplasm of mammalian cells (∼75 mg/mL of total protein).^32^ To investigate probe fluorescence in the presence of proteins, we compared the brightness of coumarin 30 (**C30** in acetonitrile) with probes **1**–**6** in PBS buffer and fetal bovine serum (FBS), which comprises 30–45 mg/mL protein,^33^ using optical spectroscopy. As shown in Figure 4, the halotag probes **4**–**6** (10 μM) in PBS (pH 7.4) showed absorbance trends consistent with our prior report (6FC > PB > 7HC),^29^ with the absorption of the 6FC-derived probe **5** comparable to **C30** in acetonitrile (ε_405nm_ = 32,000 M^–1^cm^–1^, Figure 4). Probes **4**–**6** were strongly emissive in PBS upon excitation at 405 nm, but their fluorescence was reduced when recombinant halotag protein (HTP) was added to covalently immobilize these fluorophores on the protein surface. Further addition of 50% FBS reduced fluorescence compared to PBS alone by ∼2-fold. This quenching likely results from inner filter effects (high concentrations of proteins absorbing excitation or emission light) and/or environmental quenching by aromatic amino acids. In contrast, probes **1**–**3** were extensively quenched alone in PBS but they showed substantial recovery of fluorescence in both 50% and 100% FBS, presumably because protein binding disrupted probe aggregation to restore emission from monomeric fluorophores. Although probes **4**–**6** effectively mimic the properties of simple coumarin fluorophore amides, analysis of the fluorescence properties of **1**–**3** in FBS provided the best approach to estimate the concentrations of these specific probes within cells.

**Figure 4:**
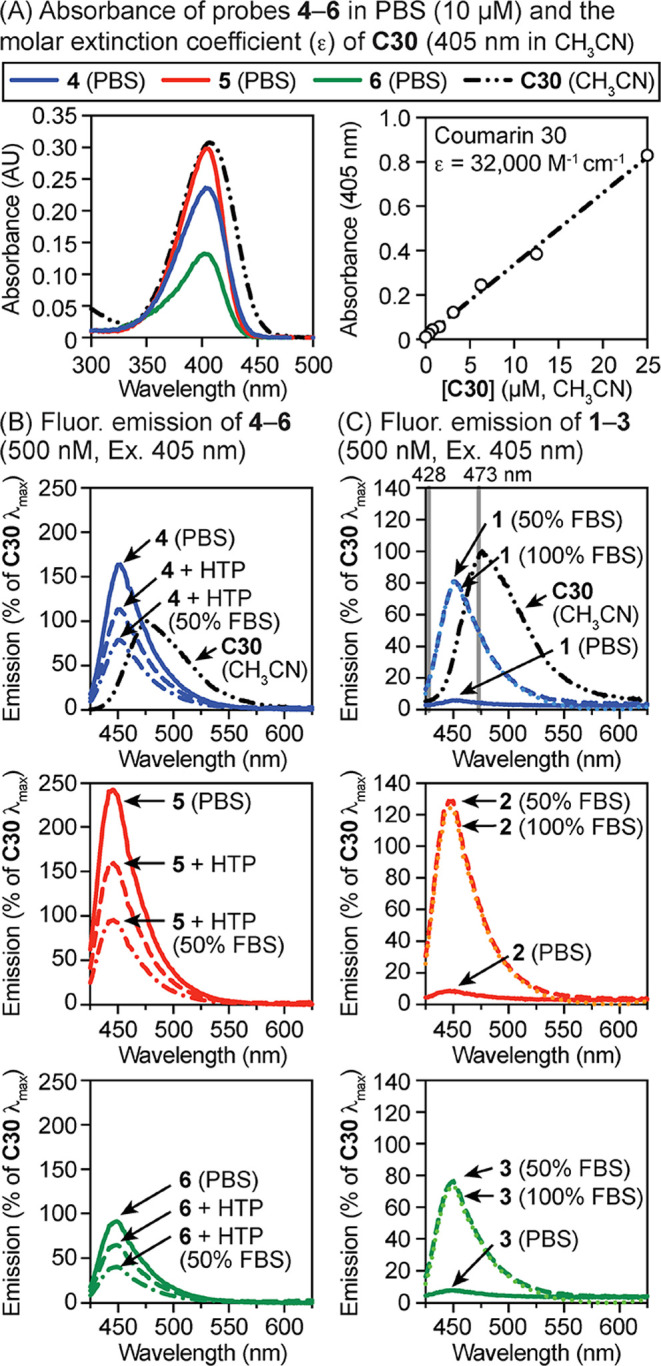
Optical spectroscopy of probes **1**–**6** (in solutions containing 1% DMSO) and **C30** in acetonitrile. (A) Absorbance spectra of **4**–**6** and **C30** acquired at 10 μM (left) and measurement of the molar extinction coefficient of **C30** in acetonitrile (right). (B, C) Normalized emission spectra acquired at 500 nM (Ex. 405 nm) in the absence and presence of proteins. In panel B, recombinant HaloTag protein (HTP) was added at 1 μM without and with 50% FBS (pH 7.3). In panel C, the emission of **1**–**3** in PBS (pH 7.4) was compared with 50% FBS (pH 7.3) and 100% FBS (pH 7.2). The range of 428–473 nm represents the bandpass filter of a CytoFLEX flow cytometer used for cellular analysis.

To analyze intracellular concentrations of **1**–**3** by qFlow, correction factors were calculated based on their emission in FBS relative to the emission of **C30** in acetonitrile after excitation at 405 nm.^34^ As shown in Table 1, these values were based on analysis of the integrated fluorescence emission from 428–473 nm (mimicking a 450/45 nm bandpass filter of a flow cytometer). Correction factors in FBS were calculated as 1.4 for probe **1** (PB-Dasatinib) 2.2 for probe **2** (6FC-Dasatinib), and 1.2 for probe **3** (7HC-Dasatinib) relative to the more red-shifted **C30**. To convert fluorescence of probes into total intracellular concentrations, a standard curve was generated by flow cytometry using NIST-standardized calibration beads with equivalent reference fluorophore (ERF)^35–39^ values provided by the manufacturer (Figure S4, Supporting Information). These values are based on the integrated emission of beads from 425–475 nm (450/50 nm) relative to **C30** in acetonitrile.^38^ In conjunction with the probe brightness correction factors in FBS, and the volume of HEK293T cells, this standard curve allowed calculation of total intracellular probe concentrations.

**Table 1:** Photophysical Properties of PB-Dasatinib (**1**), 6FC-Dasatinib (**2**), and 7HC-Dasatinib (**3**) Compared to **C30** in Acetonitrile (500 nM)^a^

probe	**1**	**2**	**3**	**C30**
ε (M^–1^ cm^–1^)	29,000	37,000	22,000	32,000
AUC_425–650nm_	48,137	73,078	42,731	90,918
AUC_428–473nm_	32,421	53,146	29,840	23,918
Protein-bound brightness_428–473nm_	**1.4**	**2.2**	**1.2**	**1.0**

a Molar extinction coefficients for **1**–**3** in PBS (10% DMSO) are based on reported^29^ coumarin *N*-hexyl amides (pH 10 for 7HC-hexylamide). The molar extinction coefficient of **C30** in acetonitrile was measured as shown in Figure 4A. AUC values of **1**–**3** in FBS (30–45 mg/mL protein, pH 7.2, 1% DMSO) and **C30** in acetonitrile from 425–650 nm and 428–473 nm were calculated from background-subtracted emission spectra. Protein-bound brightness values relative to **C30** were calculated based on AUCs from 428–473 nm to estimate emission through a 450/45 nm bandpass filter of a flow cytometer.

### Fluorophore p*K*_a_ Governs OATP1B3-Mediated Uptake of Coumarin-Dasatinib Probes

OATP1B3 is well established as a broad-specificity hepatic uptake transporter for organic anions.^16,17^ However, the extent to which fluorophore ionization state within a series of probes affects their efficiency as OATP1B3 substrates has not been systematically examined. We hypothesized that the ionization states of PB (p*K*_a_ 4.1, essentially fully anionic at pH 7.4), 6FC (p*K*_a_ 6.5, ∼90% anionic), and 7HC (p*K*_a_ 7.3, ∼50% anionic) amide derivatives would produce a gradient of OATP1B3-mediated intracellular accumulation, providing a rational basis for selecting probes optimized for different cellular contexts. To create a stable OATP1B3-expressing cell line, HEK293T cells were transfected with a PiggyBac transposon vector^40^ encoding the hSLCO1B3 gene and selected using a puromycin resistance marker (see the Supporting Information for methods). A single clone with high OATP1B3 expression was isolated by fluorescence-activated cell sorting that exhibited ∼40-fold greater uptake of the anionic fluorescent probe PB-Gly-Taxol^19^ (500 nM, 2 h) compared to the parental cells. As shown in Figure 5, confocal microscopy revealed weak uptake of PB-Gly-Taxol in parental HEK293T cells, which increased substantially upon addition of the efflux inhibitor verapamil, consistent with previous studies.^19,41^ In contrast, HEK293T cells expressing OATP1B3 exhibited strong uptake of PB-Gly-Taxol without verapamil, indicating that expression of this transporter overcomes active efflux of this coumarin-derived probe. This stable cell line derived from HEK293T was designated HC2 and used for subsequent experiments.

**Figure 5:**
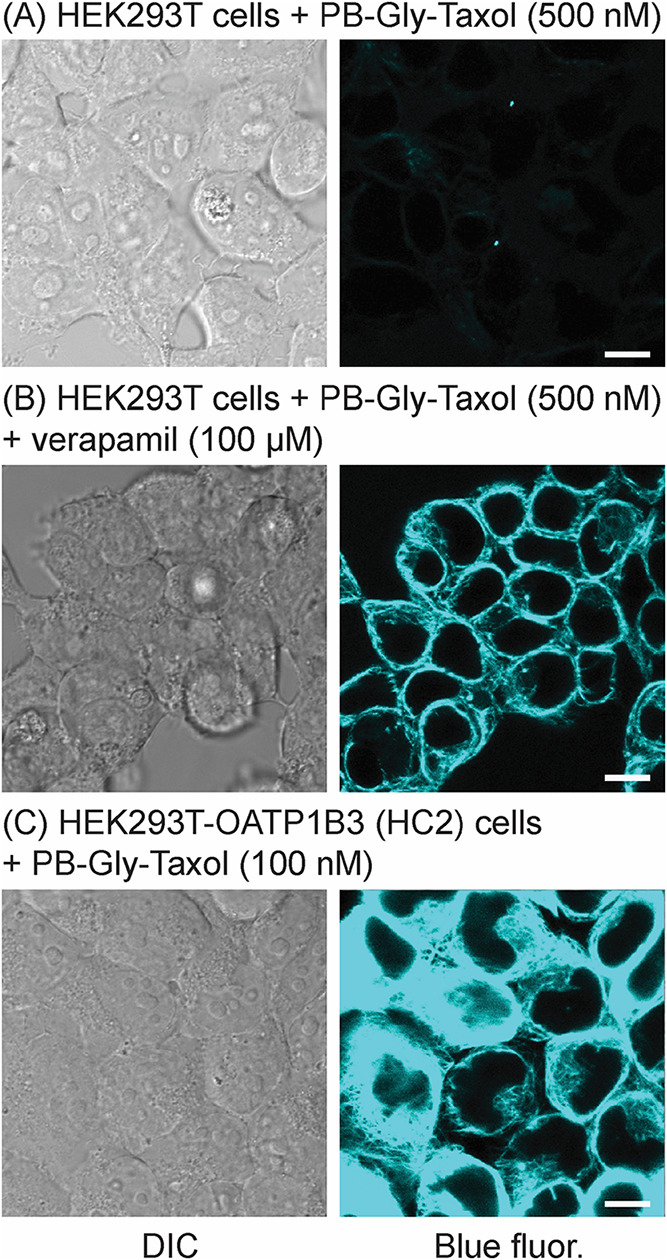
Differential interference contrast (DIC) and confocal laser scanning microscopy of HEK293T (A, B) and OATP1B3-expressing HEK293T (HC2) cells (C) treated with the anionic fluorescent probe PB-Gly-Taxol (2 h, 37 °C). Uptake of PB-Gly-Taxol (**7**) (500 nM) by HEK293T cells was minimal (A) but strongly promoted by addition of the efflux inhibitor verapamil (B, 100 μM). In contrast, stable expression of OATP1B3 by HC2 cells overcomes active efflux, promoting substantial uptake of this anionic fluorescent probe (100 nM) in the absence of verapamil (C). Scale bars = 10 μm.

The uptake of probes **1**–**3** by HEK293T and HC2 cells (Figure 6) was evaluated by flow cytometry. In HEK293T cells, nonspecific probe uptake was modest and increased linearly up to 2.5 μM, whereas HC2 cells displayed a hyperbolic fluorescence response characteristic of transporter-mediated uptake. Consistent with previous observations^29^ that more acidic coumarins will undergo greater efflux, PB-Dasatinib (**1**) showed the lowest fluorescence in HEK293T cells, whereas 6FC-Dasatinib (**2**) and 7HC-Dasatinib (**3**) showed higher nonspecific uptake. In HC2 cells, this trend was reversed with the more anionic probes **1** and **2** promoting the highest cellular fluorescence. Differences in the uptake of probe **3** in HC2 cells suggest that this more hydrophobic compound may inhibit OATP1B3 at high concentrations, resulting in uptake through an alternative pathway. Analysis by qFlow was used to convert cellular fluorescence to total intracellular probe concentrations (Figure 6B), revealing a striking reversal of accumulation order between the two cell lines: 7HC-Dasatinib (**3**), the least acidic probe, showed the greatest passive uptake in parental HEK293T cells (slope = 2.6 relative to extracellular concentration), whereas PB-Dasatinib (**1**), the most acidic, was the most efficiently taken up in OATP1B3-expressing HC2 cells (slope = 102), representing an ∼100-fold intracellular accumulation above the extracellular concentration. This inversion, from hydrophobicity-governed passive permeation in HEK293T cells to ionization state-governed active uptake in HC2 cells, demonstrates that altering fluorophore p*K*_a_ can be used to control the efficiency of OATP1B3 substrates within a probe series.

**Figure 6:**
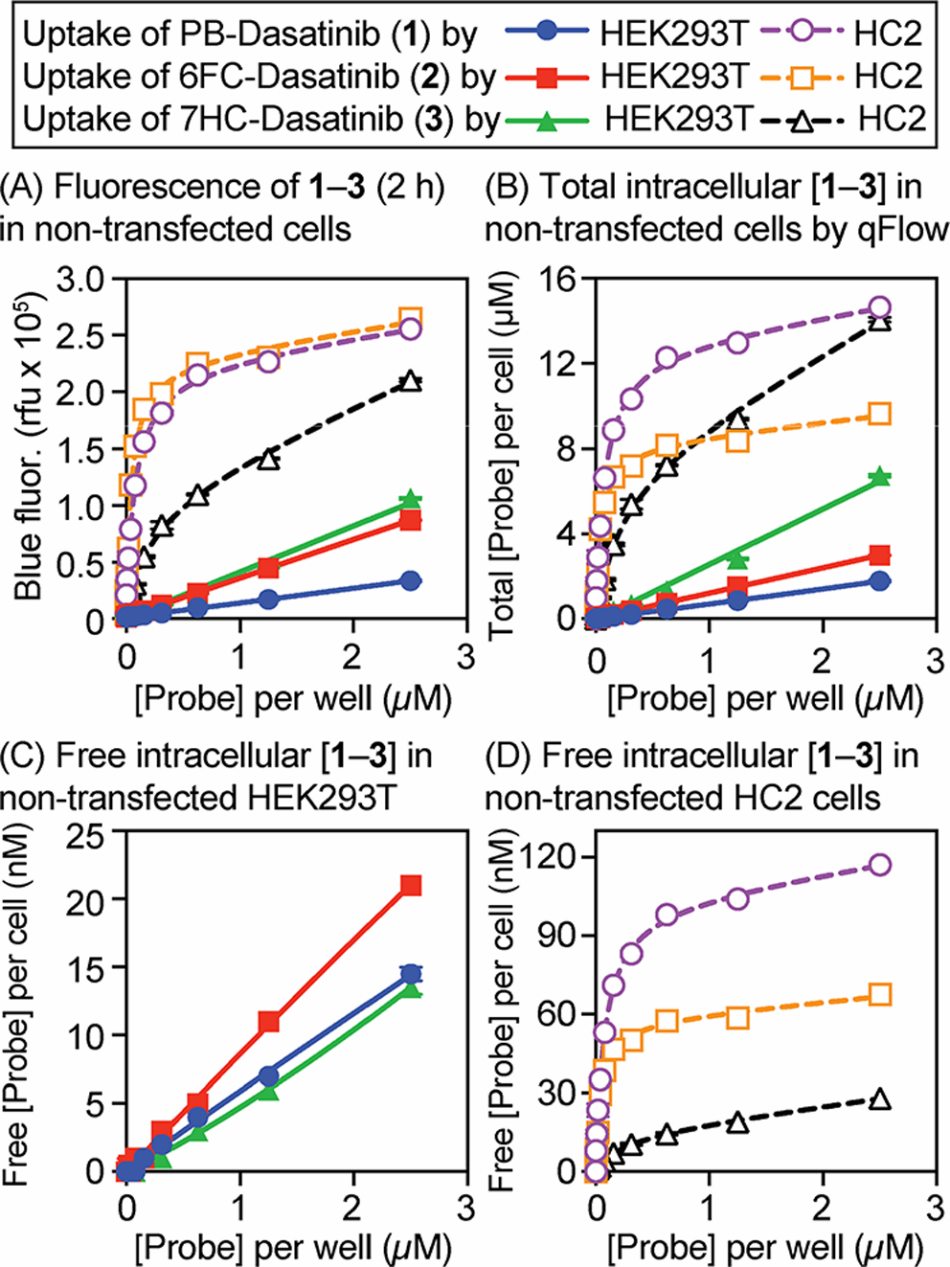
Nonspecific uptake of probes **1**–**3** by HEK293T and HC2 cells measured by flow cytometry. (A) Changes in cellular fluorescence as a function of probe concentrations. (B-D) Total (B) and free (C, D) intracellular concentrations of probes calculated by qFlow, with protein bound fractions measured by equilibrium dialysis, as a function of extracellular probe concentrations.

Linear regression of total probe concentration per well (*x*) versus per cell (*y*) yielded slopes of 0.7 (**1**), 1.2 (**2**), and 2.6 (**3**) in HEK293T cells (*R* ^2^ > 0.99), indicating that efflux reduced total intracellular concentrations of the most polar probe **1** by ∼30% compared to extracellular concentrations, the intermediate polarity probe **2** showed slightly higher intracellular compared to extracellular concentrations, and the most hydrophobic probe **3** accumulated to levels ∼3-fold higher than the applied concentration. In HC2 cells, within the linear uptake range (<50 nM), slopes for total probe uptake (*R* ^2^ = 0.89–0.98) were 102 (**1**), 100 (**2**), and 23 (**3**). This demonstrates two effects of fluorophore p*K*_a_ on OATP1B3-mediated uptake: expression of OATP1B3 enhanced intracellular accumulation of the most acidic probe, PB-Dasatinib (**1**), by ∼146-fold relative to parental HEK293T cells (HC2 slope 102 vs HEK293T slope 0.7), whereas the least acidic probe, 7HC-Dasatinib (**3**), showed a more modest ∼9-fold enhancement (HC2 slope 23 vs HEK293T slope 2.6), illustrating how systematic changes in fluorophore p*K*_a_ affect OATP1B3 substrate efficiency within this probe series.

To better understand the binding of small molecules to specific proteins expressed in these cells, we further investigated the free fraction of probes in the intracellular environment. This is important because for competitive ligand-binding assays at equilibrium, the affinity of a competitor (*K_i_*) is typically calculated using the Cheng–Prusoff equation,^42^ which relates the observed IC_50_ of the competitor to the dissociation constant (*K*_d_) of the free (unbound) probe and its free (unbound) concentration in solution. To measure the free fractions of probes **1–3**, imatinib, and dasatinib, we used equilibrium dialysis against rat liver homogenate. This method is considered^43^ to be one of the most reliable surrogates for analyzing nonspecific intracellular protein binding. We found the extent of protein binding to be 99.2% (**1**), 99.3% (**2**), 99.8% (**3**), 99.2% (dasatinib), and 97.9% (imatinib). Among the fluorescent probes, PB-Dasatinib (**1**), with the lowest p*K*_a_ (4.1) and essentially complete ionization at pH 7.4 conferred the least protein binding (0.8% ± 0.04% unbound), 6FC-Dasatinib (**2**) with an intermediate p*K*_a_ (6.5, ∼90% anionic at pH 7.4) was similar (0.7% ± 0.04% unbound), whereas 7HC-Dasatinib (**3**) with the highest p*K*_a_ (7.3, resulting in ∼50% ionization at pH 7.4) exhibited higher protein binding (0.2% ± 0.04% unbound). Dasatinib (0.8% ± 0.09% unbound) and imatinib (2.1% ± 0.4% unbound) showed comparable and slightly lower nonspecific protein binding, indicating that modification of dasatinib with PB or 6FC did not appreciably affect intracellular protein binding compared to the parent drug. Using these values, intracellular free concentrations of probes **1**–**3** were plotted against extracellular concentrations as shown in Figure 6(C,D).

Although equilibrium dialysis predicted that probes **1**–**3** exhibit high intracellular protein binding and low absolute free intracellular concentrations, we hypothesized that rapid exchange of these probes with the vast excess of probes in the extracellular environment could allow the free extracellular concentration to approximate the free intracellular concentration. In support of this hypothesis, kinetic studies in transfected HEK293T and HC2 cells by confocal microscopy revealed probe uptake half-times of <25 min at 37 °C (Figure S2, Supporting Information). This data suggests that exchange between intra- and extracellular probe pools at physiological temperature during a 2-h cellular binding assay could readily equilibrate specific and nonspecific binding. This observation, and validation of subsequent FPCBA competition binding assays, indicate that the extracellular free concentrations of these probes can approximate free intracellular ligand concentrations if equilibrium is achieved. Additionally, these assays benefit from low cell concentrations (250,000 cells/mL) and the use of low concentrations of FBS in media (4%) that help avoid complications from ligand depletion^44^ due to protein binding.

### Dasatinib, Imatinib, and Fluorescent Probes Exhibit Low Cytotoxicity toward HEK293T and HC2 Cells

Although dasatinib and imatinib are anticancer agents, these targeted drugs selectively kill CML cells by inhibiting the driver oncoprotein Bcr-Abl. Consequently, they have relatively low toxicities toward other cell types. To further establish that these compounds and fluorescent probes **1**–**3** would not be toxic toward HEK293T or HC2 cells, we measured their effects on viability of both cell lines (Figure S3, Supporting Information). Treatment with 10 μM of **1–3** or dasatinib for 24 h did not show appreciable cytotoxicity toward either cell line. Consequently, treatment of either cell line with 10 μM or less of these compounds will not affect studies by FPCBA or NanoBRET using typical equilibration periods of 2 h at 37 °C. This equilibration time has been recommended^45^ for studies of high affinity interactions that equilibrate slowly.

### Comparison of FPCBA with NanoBRET for Binding of Small Molecules to ABL1

To compare FPCBA with NanoBRET and investigate the effect of expression of OATP1B3 on target engagement, we measured cellular *K*_d_ values of probes and cellular IC_50_ and *K_i_* values of competitors in living HEK293T and HC2 cells. Compared to IC_50_, *K_i_* values are more reliable measures of binding affinity because *K_i_* is an equilibrium dissociation constant that relates directly to thermodynamics (Δ*G* = *RT *ln­(*K_i_*)), whereas IC_50_ is an assay-dependent phenomenological parameter that varies with probe concentration and affinity.^46^ In cells, ABL1 is expressed as two different isoforms: type I (ABL1a) and type II (ABL1b) that have identical kinase domains but differ by ∼20–40 amino acids at the N-terminus. To compare NanoBRET with FPCBA, we measured the apparent affinity of BODIPY-Dasatinib (**8**) for NLuc–ABL1b in HEK293T and HC2 cells. This fusion of NanoLuc to ABL1b (commercial Promega construct) prevents N-terminal myristoylation^47,48^ making this protein more closely resemble the nonmyristoylated ABL1a isoform and the oncogenic Bcr-Abl fusion protein. Saturation binding assays (2 h, 37 °C) using transiently transfected cells, together with nonspecific binding defined by addition of excess dasatinib, were fit by nonlinear regression (one-site total and nonspecific model; Figure 7A). Previous NanoBRET experiments with ABL1 indicate that this 2 h incubation should allow dasatinib and imatinib to substantially reach equilibrium in living cells based on their dissociation half-lives of 43.2 and 20.1 min.^49^ Dasatinib rapidly associates with the highly populated DFG-in (active) conformation without a large protein conformational change, whereas imatinib binds more slowly through rate-limiting induced-fit to the DFG-out (inactive) conformation.^50^

**Figure 7:**
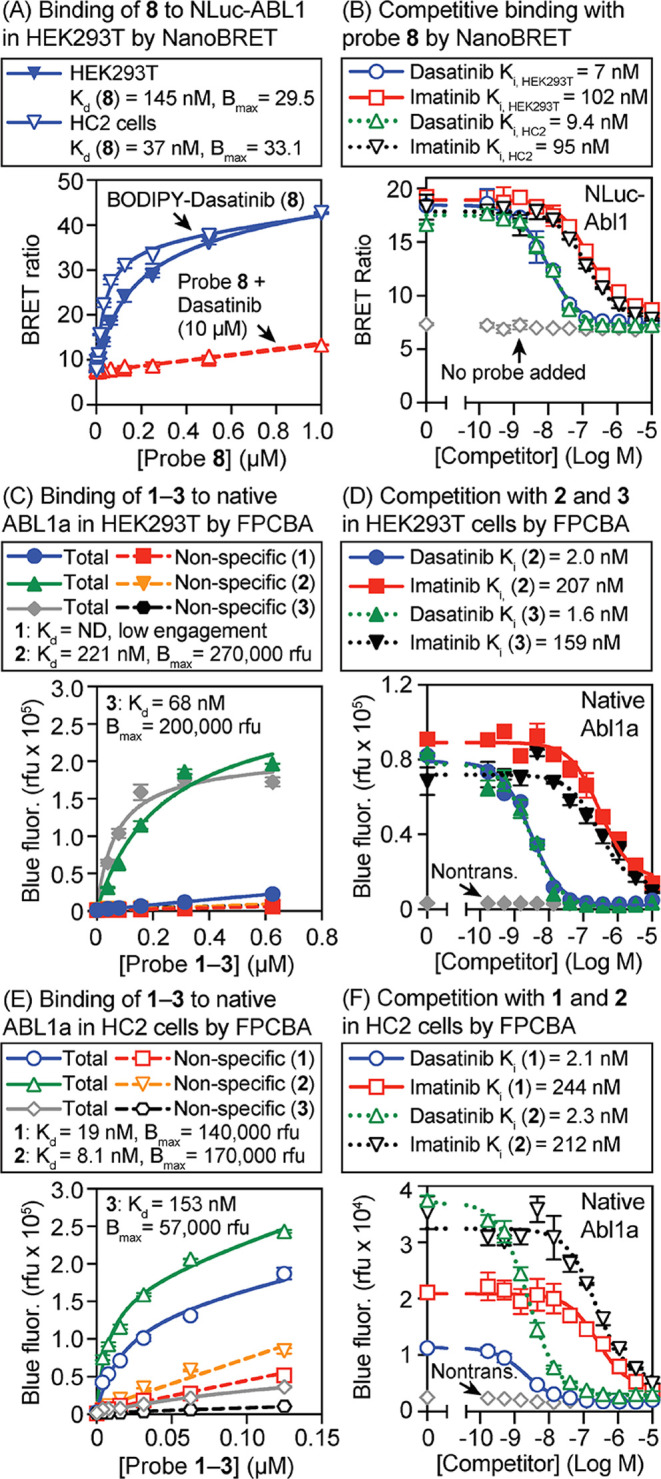
Binding and competition assays comparing NanoBRET with FPCBA. (A) Saturation binding of BODIPY-Dasatinib (**8**) to NLuc-ABL1 in HEK293T and HC2 cells by NanoBRET. (B) Competitive binding assays by NanoBRET with dasatinib and imatinib in HEK293T ([**8**] = 50 nM) and HC2 ([**8**] = 12.5 nM) cells. (C, E) Binding of probes **1**–**3** to native ABL1 in HEK293T and HC2 cells by FPCBA. (D, F) Competitive FPCBA with dasatinib and imatinib in HEK293T (D) and HC2 cells (F). In D (HEK293T), [probe **2**] = 100 nM, [probe **3**] = 50 nM. In F (HC2), [probe **1**, **2**] = 0.5 nM. Cells were treated with probes and competitors for 2 h at 37 °C prior to analysis. Nontrans.: Nontransfected. ND: Not determined due to low engagement.

OATP1B3 expression increased intracellular accumulation of probe **8**, shifting the apparent cellular *K*_d_ from 145 (126–167) nM in HEK293T (consistent with the reported 0.2 μM value^9^) to 37 (32–43) nM in HC2. These affinities were used to determine cellular *K_i_* values for binding of NLuc–ABL1 (Figure 7B) to dasatinib (*K_i_* = 7 (5–9) nM in HEK293T; 9.4 (7–12) nM in HC2) and imatinib (*K_i_* = 102 (74–141) nM in HEK293T; 95 (69–132) nM in HC2). These *K_i_* values are similar to reported NanoBRET measurements^9,49^ and indicate that neither dasatinib nor imatinib appears to be an efficient OATP1B3 substrate under these cellular assay conditions. The lower affinity of imatinib for ABL1 in HEK293T cells is consistent with its known preference for the nonphosphorylated inactive DFG-out conformation; the predominant phosphorylated active DFG-in form reported^51^ to be expressed in these cells binds imatinib more weakly. In the absence of ATP, imatinib is reported to bind purified phosphorylated ABL1 (pABL1) with biochemical *K*_d_ ∼ 20 nM, with dephosphorylation improving affinity by ∼20-fold.^52^ In contrast, dasatinib binds purified pABL1 and ABL1 similarly, with subnanomolar biochemical *K*_d_ values (0.02–0.8 nM) in ATP-free binding assays.^25,52^ In cells, competition by endogenous ATP typically reduces affinity by 10–100-fold, with larger effects observed for kinases with low ATP *K*_m_ values or inhibitors that bind rare conformational states.^9,53^

FPCBA was used to quantify binding of probes **1**–**3** and competitors to native full-length ABL1a in living HEK293T and HC2 cells as shown in Figure 7(C–F). Cells were equilibrated in suspension with small molecules for 2 h at 37 °C, and binding was immediately measured by flow cytometry by comparing blue fluorescence of transiently transfected cells with high (top 20%) versus low (bottom 20%) expression of the fluorescent marker protein mVenus. Cellular *K*_d_ values were derived by nonlinear regression using a one-site total and nonspecific binding model, where the signal from the overexpressed protein provided total binding data and the signal from nontransfected cells provided nonspecific binding data. This approach effectively corrects for nonspecific probe interactions, provided the maximum background-corrected signal-to-background (S/B) is ≥ 3-fold. This ability to distinguish transfected from nontransfected cells in a single population minimizes potential artifacts resulting from variable expression in transient transfection assays.

For FPCBA in HEK293T cells, the more hydrophobic probes **2** and **3** showed the most effective engagement of ABL1a as evidenced by affinities of *K*_d_ = 68–221 nM, high *B*_max_ (110,000–270,000 rfu), and maximal background-subtracted S/B values of >20-fold (**2**: 22–48-fold @ 78–156 nM; **3**: 78-fold at 78 nM), whereas in HC2 cells the more anionic probes **1** and **2** showed higher apparent affinities (*K*_d_ = 8–19 nM), high *B*_max_ (140,000–170,000 rfu) and maximal S/B values of 9-fold (at 8 nM). The two most effective probes in each cell line were further evaluated in competitive FPCBA with imatinib and dasatinib. For these assays, a fixed probe concentration near or below its cellular *K*_d_ was added and cellular IC_50_ values (three parameter log inhibitor vs response model) and cellular *K_i_* values based on [probe] per well (one-site Fit *K_i_* model) were calculated by nonlinear regression. Background-corrected signal-to-background (S/B) values, 95% confidence intervals, and other parameters are shown in Table S1 (Supporting Information).

Comparison of NanoBRET with FPCBA revealed general similarities and some specific differences. Similar overall trends for the lower affinity of imatinib compared to dasatinib were observed. However, imatinib bound NLuc-ABL1 with modestly higher affinity (1.4–2-fold) by NanoBRET compared to FPCBA whereas dasatinib bound weaker to NLuc-ABL1 (∼3–4-fold) compared to native ABL1. This effect is consistent with the 20 kDa NanoLuc-linker fusion protein promoting more of the DFG-out (inactive) conformation of ABL1 engaged by imatinib compared to the native untagged protein. To further investigate whether the affinity of small molecules is affected by fusion of a protein to the ABL1 N-terminus, we used FPCBA to compare mVenus-ABL1b with unmodified ABL1b expressed from an IRES-mVenus vector. As shown in Figure 8, this comparison revealed that fusion of the 29 kDa mVenus-linker to the ABL1b N-terminus reduced the affinities of probe **2**, dasatinib, and imatinib by ∼2–6 fold, indicating a benefit of studies of the physiologically more relevant native protein for analysis of these small molecule-protein interactions. Specific values are listed in Tables 2 and S1.

**Figure 8:**
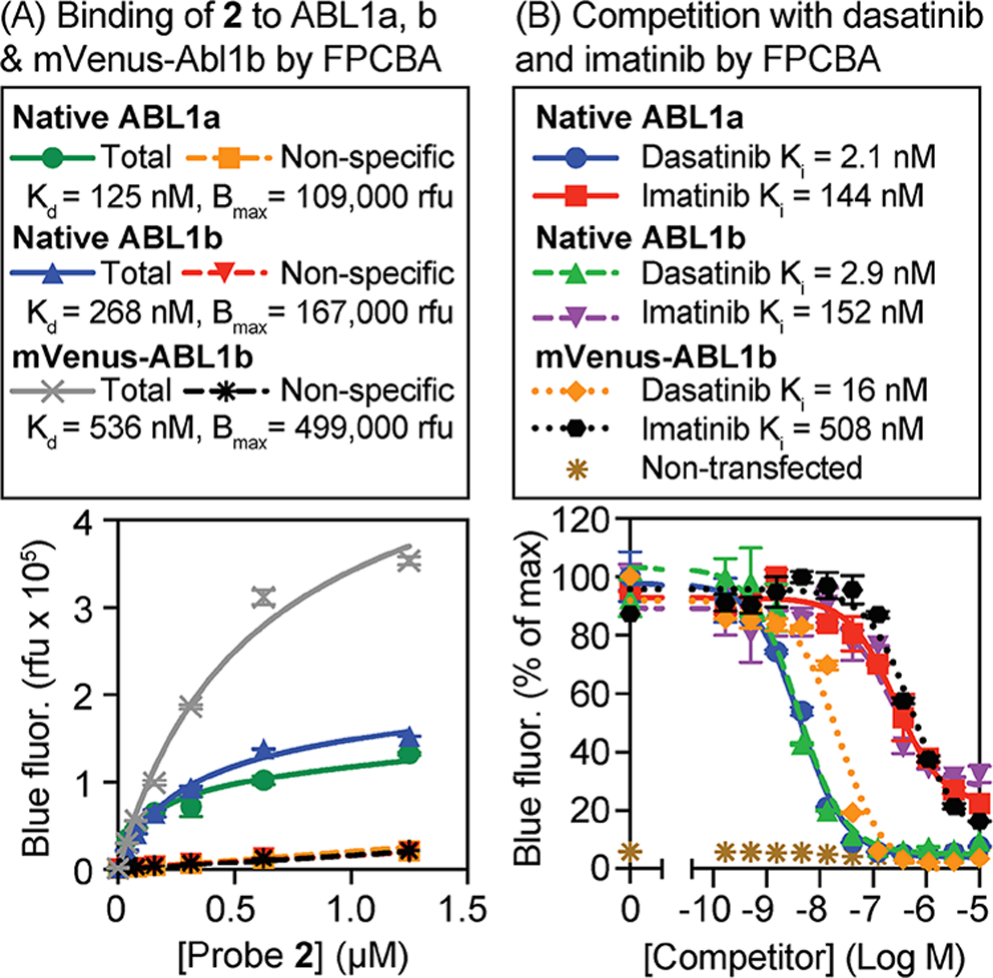
Independent side-by-side comparison of binding of small molecules to native ABL1a, native ABL1b, and mVenus-ABL1b fusion protein by FPCBA in HEK293T cells on 96-well plates. (A) Curve fits, *K*_d_ and *B*_max_ values of probe **2**. (B) Curve fits and *K_i_* values of the competitors dasatinib and imatinib. [probe **2**] = 100 nM.

**Table 2:** Cellular Affinities of Dasatinib and Imatinib for Full-Length Human Kinases by FPCBA Using 6FC-Dasatinib (**2**) as the Probe (Additional Data Used for Curve Fitting is Shown in the Supporting Information, Figures S5–S8) Compared with KINOMEscan (Recombinant Kinases without ATP)^25^ and Kinobead (Cell Lysate)^24^ Assays^a^

kinase	NCBI	FPCBA cell line- format (well)	dasatinib FPCBA *K_i_* (nM)	dasatinib kinobead *K*_d_ (nM, lysate)	dasatinib KINOME-scan *K*_d_ (nM, w/o ATP)	imatinib FPCBA *K_i_* (nM)	imatinib Kinobead *K*_d_ (nM, lysate)	imatinib KINOME-scan *K*_d_ (nM, w/o ATP)
ABL1a	NP_005148.2	HEK-96	2	5	0.029, 0.046	176	106	21*
ABL1b	NP_009297.2	HEK-96	2.8	5	0.029, 0.046	152	106	21*
mVenus-ABL1b	NP_009297.2	HEK-96	16	ND	ND	508	ND	ND
ABL2	NP_005149.4	HEK-96	4.5	4	0.17	365	64	10
BLK	NP_001706.2	HEK-384	4.5	ND	0.21	>10,000	ND	520
BMX	NP_001712.1	HEK-384	2.9	ND	1.4	>10,000	ND	>10,000
BRK	NP_005966.1	HC2–384	12	59	7.8	>10,000	>10000	>10,000
BTK	NP_000052.1	HEK-96	17	5	1.4	>10,000	>10000	>10,000
CSK	NP_004374.1	HEK-384	19	22	1	>10000	>10000	>10,000
DDR1	NP_001945.3	HEK-96	5.1	43	0.69	9.3	22	0.7
DDR2	NP_001014796.1	HEK-96	7.2	70	3.2	152	181	15
EPHA1	NP_005223.3	HC2–384	5.4	15	4.1	>10,000	>10000	>10,000
EPHA2	NP_004422.2	HEK-384	5.7	6	0.85	>10,000	>10000	>10,000
EPHA4	NP_004429.1	HEK-384	0.7	7	1.2	>10,000	>10000	>10,000
EPHA5	NP_872272.2	HEK-384	1.5	4	0.24	>10,000	>10000	>10,000
EPHA8	NP_065387.1	HEK-384	0.8	ND	0.24	>10,000	ND	1400
EPHB1	NP_004432.1	HEK-384	11	ND	0.45	>10,000	ND	>10,000
EPHB2	NP_059145.2	HEK-384	4.7	4	0.39	>10,000	>10,000	>10,000
EPHB3	NP_004434.2	HEK-384	6.5	29	6.9	>10,000	>10,000	>10,000
EPHB4	NP_004435.3	HEK-384	3.3	4	0.34	>10,000	>10,000	>10,000
FGR	NP_001036194.1	HEK-384	4.3	5	0.5	>10,000	>10,000	2400
FYN	NP_694592.1	HEK-384	3.5	4	0.79	>10,000	>10,000	3100
HCK	NP_002101.2	HC2–384	14	29	0.35	>10,000	>10,000	>10,000
LCK	NP_005347.3	HEK-384	13	7	0.2	>10,000	>10,000	40
LYN	NP_002341.1	HEK-96	2.2	8	0.57	>10,000	>10,000	890
PDGFRB	NP_002600.1	HEK-96	23	292	0.63	771	>10,000	14
SRC	NP_005408.1	HEK-96	34	3	0.21	>10,000	>10,000	>10,000

a *KINOMEscan *K*_d_ values for imatinib at ABL1 reflect the phosphorylated construct (pABL1, 21 nM); the nonphosphorylated construct gives *K*_d_ = 1.1 nM. The ∼19-fold difference is consistent with the preference of imatinib for the inactive DFG-out conformation, which is disfavored by activation-loop phosphorylation. For FPCBA, cellular probe *K*_d_, *B*_max_, S/B, IC_50_, probe concentrations, total kinase concentrations, and 95% confidence intervals are shown in Table S1. Values represent a single representative experiment except for ABL1a imatinib, which shows the mean of two independent experiments (144 and 207 nM; see Table S1). Cells were analyzed using CytoFLEX (96-well) or iQue3 (384-well) flow cytometers with cellular *K_i_* values calculated with a One site—Fit *K_i_* model. Cellular *K_i_* values were obtained in technical duplicates and replicated independently at least once. HEK: HEK293T cells. HC2: HEK293T cells stably expressing OATP1B3. ND: kinase not detected in the kinobead cell lysate panel. *K*_d_ or *K_i_* > 10,000 nM: no binding detected.

### Profiling of Tyrosine Kinases by FPCBA

To evaluate the generality of FPCBA beyond ABL1, we profiled probe **2** and the competitors dasatinib and imatinib against a panel of 24 additional full-length native human kinases (Tables 2, S1, and Figures S5–S8). In HEK293T cells, most of these proteins bound probe **2** with S/B ≥ 6, but some kinases with low efficacy were studied in HC2 cells to improve S/B and facilitate analysis. To increase throughput, most of these kinases were screened in 384-well format using an iQue3 high throughput flow cytometer, but some were analyzed in 96-well format on a CytoFLEX S instrument. Both platforms yielded consistent *K_i_* values for competition by dasatinib. Probe **2** bound all kinases tested, with cellular *K*_d_ values in HEK293T cells spanning 2 orders of magnitude from 27 nM (EPHA4) to 1700 nM (BTK) and S/B of 6- to 56-fold. Cellular *K_i_* values for dasatinib were uniformly in the low nanomolar range (0.7–34 nM), with the highest affinity observed for EPHA4 (0.7 nM) and EPHA8 (0.8 nM), and the lowest for SRC (34 nM). Beyond ABL1, imatinib showed measurable cellular binding only to kinases known^54–56^ to adopt the DFG-out conformation, including DDR1 (*K_i_* = 9.3 nM), DDR2 (*K_i_* = 152 nM), ABL2 (*K_i_* = 365 nM), and PDGFRB (*K_i_* = 771 nM), with all other kinases exceeding 10 μM. In HC2 cells, OATP1B3-mediated probe accumulation markedly enhanced the apparent affinity of probe **2**, enabling FPCBA measurements at substantially lower probe concentrations, with improved S/B for BRK, EPHA1 and HCK. Although imatinib has been reported to inhibit transport by OATP1B3,^57,58^ its affinity for ABL1a in HEK293T and HC2 cells was within experimental error (Table S1), indicating low inhibition of this transporter under these conditions. These FPCBA-derived *K_i_* values were compared with two established biochemical platforms: KINOMEscan,^25^ which measures binding to recombinant, C-terminally truncated kinase domains in the absence of ATP, and kinobeads,^24^ which measures binding to endogenous full-length kinases captured from lysates of K562 CML cells by quantitative mass spectrometry (Table 2). Concordances and divergences are described in the Discussion section.

## Discussion

FPCBA expands flow cytometry beyond cell-surface receptors, enabling quantitative measurement of binding of ligands to native untagged intracellular proteins in living cells. Here, we extended FPCBA to small molecules that engage ATP binding sites of tyrosine kinases. We also explored the use of OATP1B3-mediated uptake to enhance intracellular probe accumulation and implemented quantitative flow cytometry (qFlow) with NIST-calibrated bead standards to directly measure intracellular probe concentrations. Systematic tuning of the acidity of three coumarin-derived probes (p*K*_a_ 4.1–7.3)^29^ revealed that 6FC-Dasatinib (**2**) provided an optimal balance of brightness, moderate acidity, and uptake/efflux in the two cell types studied. In OATP1B3-expressing HC2 cells, acidic probes (PB, 6FC) accumulated ∼ 100-fold above extracellular concentrations, improving apparent *K*_d_ values by 20–27-fold. These concentrations were measured using qFlow calibration with brightness correction factors derived from FBS to mimic the protein-rich intracellular environment and account for aggregation-dependent fluorescence quenching in aqueous buffer. A benefit of FPCBA compared to biochemical binding assays is that it is an open no-wash system, where the extracellular medium serves as a reservoir of free ligand that can circumvent the sensitivity-limiting tight-binding regime when probe concentrations substantially exceed bulk target concentrations.^45,46^ In cases where probe and bulk kinase concentrations are comparable, as in HC2 cells at low probe concentrations, some operation in the tight-binding regime cannot be excluded, but the resulting *K_i_* values remain internally self-consistent because the cellular *K*_d_ used in the Cheng–Prusoff equation is measured under identical conditions. Rapid probe exchange (*t*_1/2_ < 25 min at 37 °C) indicates that extracellular free concentrations will approximate intracellular free concentrations at equilibrium, despite extensive intracellular nonspecific protein binding of probes and competitors (>97% bound). This enables direct application of Cheng–Prusoff analysis,^42^ without requiring independent measurement of intracellular free fractions,^59^ to quantify molecular interactions in complex biological systems. These considerations indicate that the limit of detection for FPCBA will be predominantly controlled by the kinetics of equilibration. FPCBA should achieve a subnanomolar detection limit for type I kinase inhibitors exhibiting fast binding kinetics (*k*_on_ ∼ 5 × 10^6^ M^–1^s^–1^, *k*_off_ > 0.01 s^–1^),^60^ exemplified by dasatinib, when equilibrated for 2 h at 37 °C with a probe whose uptake *t*_1/2_ is <30 min.^61^

In a side-by-side comparison, FPCBA and NanoBRET showed similar trends in affinity where dasatinib binds substantially more tightly than imatinib to ABL1. However, studies of untagged ABL1 by FPCBA consistently revealed a 3–4-fold higher affinity of dasatinib and a 1.4–2-fold lower affinity of imatinib compared to NanoBRET. These results are consistent with the NanoLuc fusion protein required for NanoBRET affecting regulatory conformational states of ABL1 that are differentially bound by dasatinib and imatinib. An additional controlled comparison of ABL1b with an analogous ABL1b-mVenus fusion protein by FPCBA showed that fusion of this protein to the ABL1b N-terminus reduced affinity by ∼6-fold for dasatinib (2.8 nM vs 16 nM) and ∼3-fold for imatinib (152 nM vs 508 nM) compared with unmodified ABL1b, further confirming that N-terminal tagging can substantially perturb inhibitor binding. Interestingly, comparison of the two native isoforms revealed that ABL1a (*K_i_* = 1.6–2.2 nM dasatinib; 144–207 nM imatinib in HEK293T) and ABL1b (*K_i_* = 2.8 nM dasatinib; 152 nM imatinib in HEK293T) bind both drugs with broadly similar affinity, despite the fact that ABL1b possesses the N-terminal Gly2 myristoylation site that mediates autoinhibitory myristate-to-C-lobe interactions whereas ABL1a lacks this site. This suggests that the affinity reduction caused by N-terminal protein fusions is primarily a steric effect of the bulky tag disrupting N-terminal cap regulatory interactions, rather than a specific consequence of lost myristoylation. These results highlight a key advantage of FPCBA: direct measurement of target engagement for native, unmodified proteins while maximally preserving post-translational modifications and regulatory interactions that govern cellular pharmacology.

To assess the broader validity of FPCBA for studies of native proteins in living cells, we measured cellular *K_i_* values of dasatinib and imatinib for 25 kinases with published biochemical affinities. These FPCBA values were compared with two complementary platforms: KINOMEscan,^25^ which measures binding to recombinant kinase domains in an ATP-free cell-free format, and kinobeads,^24^ which measures binding to endogenous full-length kinases captured from cancer cell lysate by quantitative mass spectrometry (Table 2). Of the 21 kinases detected in both the FPCBA and kinobead panels (counting ABL1a and ABL1b as a single entry since kinobead does not distinguish between them), 14 showed concordant affinities for dasatinib within 4-fold. Concordant kinases spanned the Abl family (ABL1a: 2 vs 5 nM; ABL2: 4.5 vs 4 nM), multiple Src-family kinases (FYN: 3.5 vs 4 nM; FGR: 4.3 vs 5 nM; LYN: 2.2 vs 8 nM), and Eph receptors (EPHA2: 5.7 vs 6 nM; EPHB4: 3.3 vs 4 nM). The seven discordant kinases fell into two mechanistic categories: SRC (34 vs 3 nM, 11-fold) showed weaker affinity in living cells than in lysate, consistent with autoinhibitory regulation disrupted by lysis; whereas DDR1, DDR2, EPHA4, PDGFRB, and the borderline cases BRK (12 vs 59 nM, 4.9-fold) and EPHB3 (6.5 vs 29 nM, 4.5-fold) all showed tighter binding by FPCBA than by kinobeads, consistent with intact cellular or membrane-dependent conformational states that favor inhibitor engagement. For ABL1, dasatinib showed a 35–76-fold shift in affinity comparing the isolated kinase domain in the absence of ATP (KINOMEscan) with FPCBA (0.029–0.046 vs 1.6–2.2 nM in HEK293T), consistent with binding to the active conformation of ABL1 in cells and competition with endogenous ATP.^26,53,62^ Imatinib showed a lower 6–10-fold shift (21 vs 144–207 nM in HEK293T), consistent with selective binding to the inactive DFG-out conformation of ABL1, where the effective *K*_m_ for ATP is higher.^26,62^ Imatinib was concordant between FPCBA and kinobeads for three of four targets with measurable affinity in both platforms, DDR1, DDR2, and ABL1 (treating the ABL1a/ABL1b isoforms as a single entry), including DDR1 (9.3 vs 22 nM), DDR2 (152 vs 181 nM), ABL1a (144–207 vs 106 nM), and ABL1b (152 vs 106 nM). ABL2 was an exception: dasatinib showed excellent concordance (FPCBA *K_i_* = 4.5 nM vs kinobead *K*_d_ = 4 nM), but imatinib was 6-fold less potent in living cells than in lysate (FPCBA *K_i_* = 365 nM vs kinobead *K*_d_ = 64 nM). This selective disconnect for the conformation-selective inhibitor imatinib, but not the conformation-promiscuous inhibitor dasatinib, suggests that native ABL2 in living cells populates the inactive DFG-out conformation less readily than ABL2 captured from cell lysate, consistent with the broader pattern of conformation-dependent variability observed for imatinib across the Abl family. In contrast, KINOMEscan *K*_d_ values for recombinant kinase domains were consistently 5- to 170-fold more potent than either FPCBA or kinobead values (e.g., dasatinib at SRC: 0.21 nM KINOMEscan vs 3 nM kinobead vs 34 nM FPCBA; imatinib at ABL1: 21 nM KINOMEscan vs 106 nM kinobead vs 207 nM FPCBA). Studies of CSK (FPCBA *K_i_* = 19 nM; kinobead *K*_d_ = 22 nM; KINOMEscan *K*_d_ = 1 nM) revealed agreement of FPCBA with kinobeads but divergence from KINOMEscan. This is consistent with the recombinant cell-free format of KINOMEscan overestimating potency by eliminating conformational gating, ATP competition, and regulatory interactions present in more native contexts.

The most informative disconnects observed were those where FPCBA diverged from both biochemical platforms. SRC showed at least 11-fold lower potency in live cells (*K_i_* = 34 nM) than in either kinobead or KINOMEscan assays (3 and 0.21 nM, respectively), consistent with autoinhibition of SRC by intramolecular SH3-SH2-kinase domain interactions^63,64^ maintained by endogenous CSK-mediated phosphorylation in intact cells. This regulatory mechanism is likely disrupted by cell lysis and is absent using a recombinant protein. Conversely, DDR1, DDR2, and EPHA4 were 8- to 10-fold more potent by FPCBA than by kinobeads for dasatinib (DDR1: 5.1 vs 43 nM; DDR2: 7.2 vs 70 nM; EPHA4: 0.7 vs 7 nM), indicating that the full-length membrane-anchored conformations of these receptor tyrosine kinases in living cells favor inhibitor binding more than the endogenous kinases captured from cell lysate. For EPHA4, FPCBA even revealed slightly higher affinity than KINOMEscan (0.68 vs 1.2 nM), suggesting that cellular membrane anchoring further enhances dasatinib engagement beyond the kinase domain alone. These findings align with the recent report by Binder, Robers, and Axtman,^65^ who demonstrated using NanoBRET that cellular context affects kinase inhibitor selectivity profiles, with type II inhibitors engaging targets in live cells that are missed by cell-free profiling. FPCBA extends these observations by providing quantitative *K_i_* values for native, untagged proteins. This approach enables direct comparison with equilibrium dissociation constants from biochemical assays without potential effects of genetic fusions on kinase regulation.

A practical consideration when applying FPCBA in HC2 cells is the possibility that test compounds may interact with OATP1B3 as substrates or inhibitors. These interactions could alter intracellular probe concentrations independent of competition at the target binding site. We reported previously^18^ and showed here that PB-Gly-Taxol (**7**) is an OATP1B3 substrate that accumulates in HC2 cells ∼40-fold more efficiently than in parental HEK293T cells. This probe provides a microtubule-binding control that can be used to examine whether compounds affect OATP1B3 independent of interactions of other target proteins. If compounds affect OATP1B3 using this assay, cross-validation of *K_i_* values in parental HEK293T cells (when S/B is sufficient) or using an orthogonal method is recommended.

## Conclusion

The generalizability of FPCBA rests on the requirement that the target protein can be overexpressed in a host cell line relative to endogenous background, and that a cell-permeable fluorescent probe can be identified whose cellular uptake is detectably enhanced by binding to a specific target. Having demonstrated FPCBA with allosteric^13^ and orthosteric modulators of kinases, we anticipate that the platform is broadly applicable to numerous intracellular protein classes, or extracellular domains of membrane proteins, for which a fluorescent ligand suitable for flow cytometry can be designed. The coumarin probe design principles established here, including systematic tuning of p*K*_a_ and related physicochemical properties to balance brightness, permeability, and sensitivity to efflux and influx transporters, provide a generalizable approach for probe development beyond kinases. The IRES-mVenus expression vector used here is one method for identifying transfected cells expressing the target, and the open-system thermodynamic framework applies regardless of the expression strategy or target class. We are currently extending FPCBA to additional protein classes.

## Supplementary Material



## Notes

Synthesis of fluorescent probes, determination of calibration parameters for flow cytometers, NMR spectra, methods, and compound characterization data (PDF)
