# Supplementary material for: Quantification of Binding of Small Molecules to Native Kinases by Flow Cytometry Reveals Divergence from Biochemical Affinities
Source: J Am Chem Soc. 2026 Jun 23;148(26):27613–25. doi: 10.1021/jacs.6c06577 (PMC13352625; doi:10.1021/jacs.6c06577)
Supplement: Supplementary file 1 [file ja6c06577_si_001.pdf]

Supporting Information

Quantification of Binding of Small Molecules to Native Kinases by Flow Cytometry Reveals Divergence from Biochemical Affinities

Lillian M. Cool,[§] Jogendra Pawar,[†] Sonam Sonam,[§] Smita Kumari,[§] Serena Li Zhao,[§] Xiaojun Hu,[§] Zhihong Lin,[†] Meng Wu,[§,†] Shuiying Hu,[‡,†] and Blake R. Peterson[§,†]*

[§]Division of Medicinal Chemistry and Pharmacognosy, [‡]Division of Pharmaceutics and Pharmacology, The Ohio State University, College of Pharmacy, Columbus, OH, USA.

[†]The Ohio State University Comprehensive Cancer Center – Arthur G. James Cancer Hospital and Richard J. Solove Research Institute, Columbus, OH, USA.

Contents | **Page**

Figure S1. Synthesis of fluorescent probes. S2

Figure S2. Kinetics of cellular uptake of probes **1**–**3**. S2

Figure S3. Cytotoxicity of imatinib, dasatinib and **1**–**3** towards HEK293T and HC2 cells. S3

Figure S4. Determination of calibration parameters for flow cytometers. S3

Table S1. FPCBA affinities of probes and inhibitors and related parameters for kinases. S4

Figures S5–S8. FPCBA binding curves for **2,** imatinib, and dasatinib in living cells. S5–S8

General experimental section for synthesis and compound characterization data S9

Figures S9–S14. Analytical HPLC profiles of probes subjected to biological evaluation. S14–S19

Figures S15-S38. NMR spectra. S20–S31

Biological methods S32–S49

Figure S39. Morphology of the HC2 (HEK293T-OATP1B3) cell line. S33

References for the supporting information S49

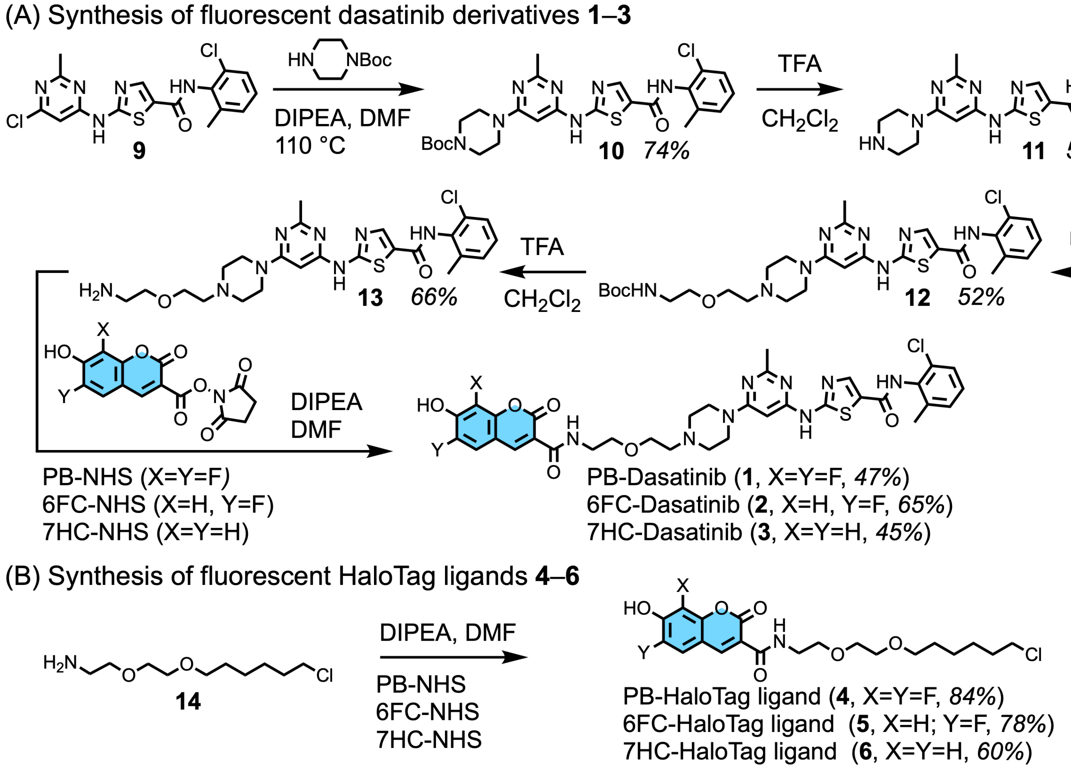

Figure S1. Synthesis of fluorescent dasatinib derivatives **1**–**3** (A) and HaloTag ligands **4**–**6** (B).

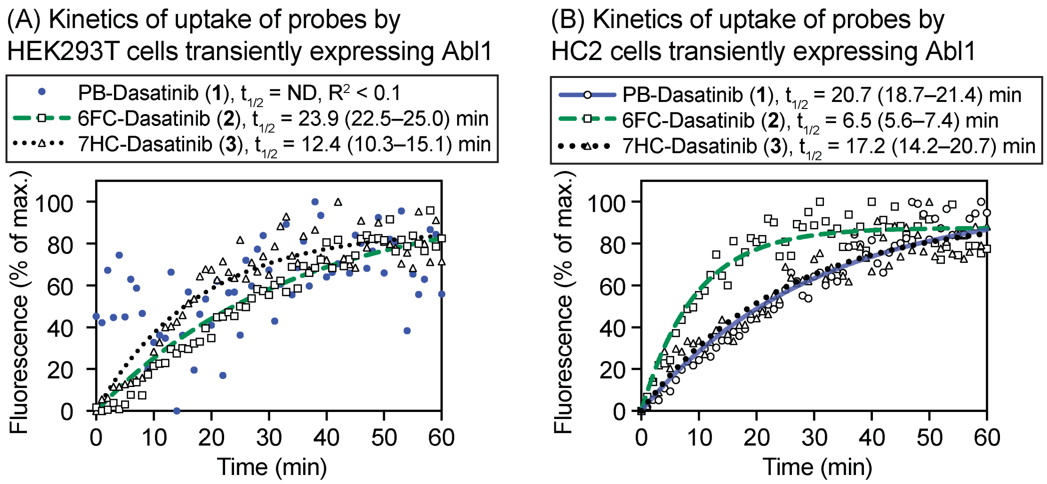

Figure S2. Kinetics of cellular uptake of probes **1**–**3** by living HEK293T (1 µM probe) and HC2 cells (100 nM probe) transiently transfected with ABL1a-IRES-mVenus at 37 °C by confocal microscopy. In HEK293T cells, the least polar probes **2** and **3** showed the most effective uptake with half times of 23.9 (**2,** $R^2 = 0.95$) and 12.4 (**3**, $R^2 = 0.89$) minutes, whereas the most polar probe PB-Dasatinib (**1**) exhibited low permeability and fit poorly to the one-phase association model ($R^2 < 0.1$, curve fit not shown). In HC2 cells, the most anionic probes **1** and **2** showed the most effective uptake with half times of 20.7 (**1**, $R^2 = 0.94$) and 6.5 (**2**, $R^2 = 0.90$) minutes. In this cell line, the more hydrophobic 7HC-Dasatinib (**3**) exhibited lower uptake (likely associated with some substrate inhibition of OATP1B3) and a half-time of 17.2 (**3**, $R^2 = 0.92$) minutes. With the exception of PB-Dasatinib (**1**) in HEK293T, these probes exhibited > 96% equilibration (5 half-times) after treatment of cells for 2 h, which was the incubation time used for FPCBA. Data points are representative of two independent replicates. Ranges in half-times are 95% confidence intervals from curve fitting.

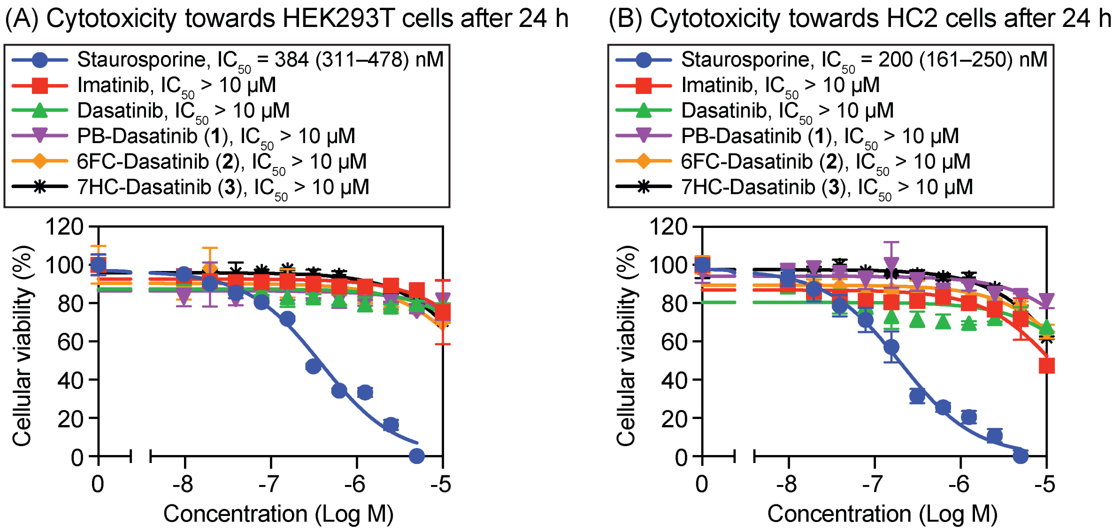

Figure S3. Cytotoxicity of imatinib, dasatinib, and probes **1**–**3** towards HEK293T (A) and HC2 (B) cells after treatment for 24 h at 37 °C. Viability was measured with CellTiter-Glo 2.0 (Promega). Compared to the pan kinase inhibitor staurosporine as a cytotoxic positive control, imatinib, dasatinib, and probes **1**–**3** exhibited low cytotoxicities with IC_{50} values of > 10 μM. Data was fit to a log(inhibitor) vs. response (three parameters) model (GraphPad Prism). Ranges in parenthesis represent 95% confidence intervals from curve fitting.

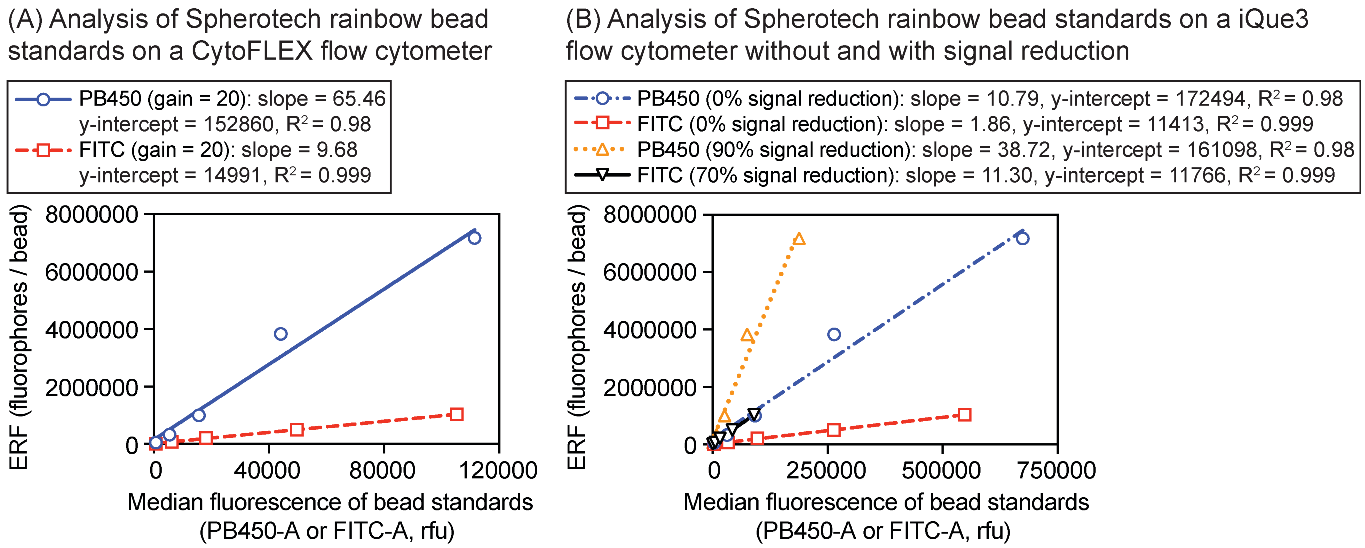

Figure S4. Determination of parameters for quantitative flow cytometry on CytoFLEX (96-well) and iQue3 (384-well) instruments. NIST-Standardized Spherotech rainbow calibration beads were analyzed on 96-well plates with the CytoFLEX and on 384-well plates with the iQue3 flow cytometer. On the iQue3, signal reduction values of 90% (PB450) and 70% (FITC) were used to allow more consistent comparisons of data obtained on the two instruments (values without and with signal reduction are shown).

Kinase (probe)	Cell line-format (well)	Cellular K_d of probe (nM)	Cellular B_{max} of probe (x 10^5, rfu)	Bg-sub S/B (@ nM)	Est. Bulk [Kinase] (nM)	[Probe] for K_i (nM)	Dasatinib FPCBA IC_{50} (nM)	Dasatinib FPCBA K_i (nM)	Imatinib FPCBA IC_{50} (nM)	Imatinib FPCBA K_i (nM)
ABL1a (**1**)	HC2-96	19 (12–28)	1.4 (1.2–1.6)	9.3 (7.8)	0.96	0.5	2.1 (1.8–2.6)	2.1 (1.7–2.5)	251 (139–449)	244 (135–437)
ABL1a (**2**)	HEK-96	221 (159–310)	2.7 (2.3–3.2)	48 (78)	1.2	100	2.9 (2.3–3.5)	2 (1.6–2.4)	300 (189–477)	207 (130–328)
ABL1a (**2**)	HEK-96	125 (78–202)	1.1 (1.0–1.3)	22 (156)	0.47	100	4 (3.3–4.9)	2.2 (1.8–2.7)	259 (170–395)	144 (94–220)
ABL1a (**2**)	HC2-96	8.1 (5.8–11)	1.7 (1.5–1.8)	9.2 (7.8)	0.72	0.5	2.5 (2.2–2.8)	2.3 (2.0–2.6)	225 (134–379)	212 (126–357)
ABL1a (**3**)	HEK-96	68 (48–96)	2.0 (1.8–2.2)	78 (78)	1.6	50	2.8 (2.1–3.8)	1.6 (1.2–2.2)	276 (139–555)	159 (80–320)
ABL1b (**2**)	HEK-96	268 (223–321)	1.7 (1.6–1.8)	22 (156)	0.72	100	3.9 (2.7–5.7)	2.8 (2.0–4.1)	209 (115–376)	152 (84–273)
mVenus-ABL1b	HEK-96	536 (416–696)	5.0 (4.4–5.7)	34 (156)	2.2	100	19 (13–27)	16 (11–23)	602 (395–928)	508 (332–782)
ABL2 (**2**)	HEK-96	125 (109–142)	4.9 (4.7–5.2)	44 (78)	2.1	100	8 (6.7–9.6)	4.5 (3.7–5.3)	657 (401–1083)	365 (223–601)
BLK (**2**)	HEK-384	140 (109–179)	2.5 (2.2–2.7)	13 (39)	0.63	20	5.1 (3.9–6.6)	4.5 (3.4–5.8)	> 10000	> 10000
BMX (**2**)	HEK-384	412 (262–685)	5.4 (4.3–7.3)	13 (625)	1.4	500	6.3 (4.3–9.4)	2.9 (1.9–4.2)	> 10000	> 10000
BRK (**2**)	HC2-384	13 (9.1–20)	3.3 (2.8–4.0)	9.6 (16)	0.84	20	30 (22–41)	12 (8.8–16)	> 10000	> 10000
BTK (**2**)	HEK-96	1708 (1285–2380)	3.3 (2.7–4.2)	9.3 (313)	1.4	500	21 (14–33)	17 (11–25)	> 10000	> 10000
CSK (**2**)	HEK-384	965 (728–1314)	6.6 (5.6–7.9)	11 (625)	1.7	100	21 (14–32)	19 (12–29)	> 10000	> 10000
DDR1 (**2**)	HEK-96	296 (239–370)	4.5 (4.1–5.0)	35 (78)	1.9	100	6.8 (5.1–9.2)	5.1 (3.8–6.9)	12 (9.2–17)	9.3 (6.9–13)
DDR2 (**2**)	HEK-96	276 (203–381)	2.4 (2.1–2.8)	25 (313)	1.0	100	9.9 (6.3–16)	7.2 (4.6–12)	206 (129–327)	152 (95–240)
EPHA1 (**2**)	HC2-384	4.1 (3.1–5.4)	1.8 (1.7–2.0)	12 (8)	0.47	8	16 (11–23)	5.4 (3.7–7.8)	> 10000	> 10000
EPHA2 (**2**)	HEK-384	215 (181–255)	4.9 (4.6–5.4)	14 (313)	1.3	20	6.2 (3.9–9.7)	5.7 (3.6–8.9)	> 10000	> 10000
EPHA4 (**2**)	HEK-384	27 (19–37)	0.8 (0.7–0.8)	11 (20)	0.19	20	1.2 (0.91–1.5)	0.68 (0.52–0.88)	> 10000	> 10000
EPHA5 (**2**)	HEK-384	51 (34–79)	2.0 (1.6–2.5)	13 (10)	0.5	50	2.9 (2.1–4)	1.5 (1.1–2.0)	> 10000	> 10000
EPHA8 (**2**)	HEK-384	60 (46–77)	2.1 (1.9–2.4)	13 (10)	0.54	100	2 (1.6–2.5)	0.76 (0.62–0.94)	> 10000	> 10000
EPHB1 (**2**)	HEK-384	467 (377–587)	8.8 (7.8–10.1)	24 (625)	2.3	100	14 (9.9–19)	11 (8.1–16)	> 10000	> 10000
EPHB2 (**2**)	HEK-384	193 (133–284)	6.3 (5.3–7.5)	22 (313)	1.6	100	7.1 (5.5–9.2)	4.7 (3.7–6)	> 10000	> 10000
EPHB3 (**2**)	HEK-384	508 (370–722)	2.6 (2.2–3.2)	5.5 (625)	0.67	200	9.1 (6.9–12)	6.5 (5–8.6)	> 10000	> 10000
EPHB4 (**2**)	HEK-384	81 (61–107)	4.2 (3.7–4.7)	20 (78)	1.1	20	4.1 (3–5.8)	3.3 (2.4–4.7)	> 10000	> 10000
FGR (**2**)	HEK-384	172 (146–203)	3.1 (2.9–3.3)	13 (156)	0.79	20	4.8 (3.7–6.4)	4.3 (3.3–5.8)	> 10000	> 10000
FYN (**2**)	HEK-384	88 (72–108)	2.8 (2.5–3.0)	16 (20)	0.71	20	4.3 (3.5–5.2)	3.5 (2.9–4.2)	> 10000	> 10000
HCK (**2**)	HC2-384	6.8 (4.3–11)	2.0 (1.6–2.4)	8.1 (16)	0.5	15	45 (13–178)	14 (4–56)	> 10000	> 10000
LCK (**2**)	HEK-384	266 (213–333)	12.8 (11.5–14.4)	36 (313)	3.3	20	14 (10–19)	13 (9.6–18)	> 10000	> 10000
LYN (**2**)	HEK-96	197 (147–265)	2.6 (2.3–2.9)	38 (78)	1.1	500	7.6 (6.3–9.2)	2.2 (1.8–2.6)	> 10000	> 10000
PDGFRB (**2**)	HEK-96	1123 (776–1795)	2.9 (2.2–4.1)	10 (78)	1.2	500	34 (24–47)	23 (17–33)	1115 (294–6623)	771 (204–4584)
SRC (**2**)	HEK-96	333 (285–390)	13.5 (12.6–14.4)	56 (156)	5.8	100	45 (32–62)	34 (25–48)	> 10000	> 10000

Table S1. Representative cellular affinities and associated parameters of kinases for probes, dasatinib, and imatinib by FPCBA. Two independent replicates are shown for ABL1a with probe **2** (data shown in Figures 7 and 8 of the main text). With the exception of background-subtracted (Bg-sub) S/B, where the value in parenthesis is the concentration observed at maximal S/B in nM, values in parentheses represent 95% confidence intervals from curve fitting.

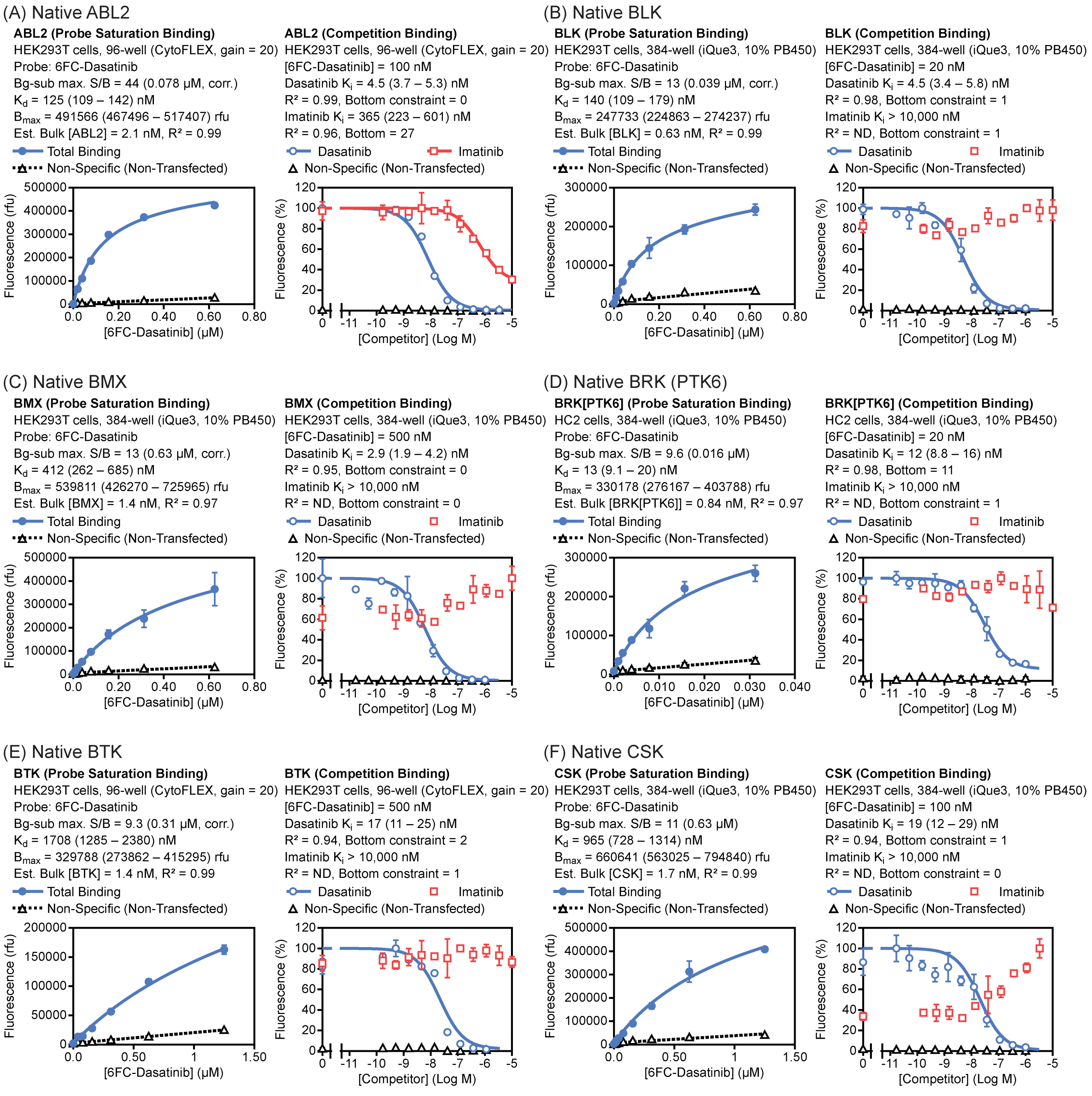

Figure S5. Nonlinear curve fitting for binding of 6FC-Dasatinib (probe **2**, One Site – Total and Nonspecific Binding model) and the competitors dasatinib and imatinib (One Site – Fit Ki model) to native full-length human ABL2, BLK, BMX, BRK (PTK6), BTK, and CSK by FPCBA. For curves where Bottom was manually constrained to improve fit stability, Bottom was fixed to the mean fluorescence of that curve's own non-specific-binding wells. Probe Bg-sub max. S/B values labeled "corr." were corrected for a denominator-collapse artifact at non-specific signal <10% of peak. For calculated values, parenthetical numbers represent 95% confidence intervals from curve fitting. ND: no specific binding observed (K_i > 10,000 nM).

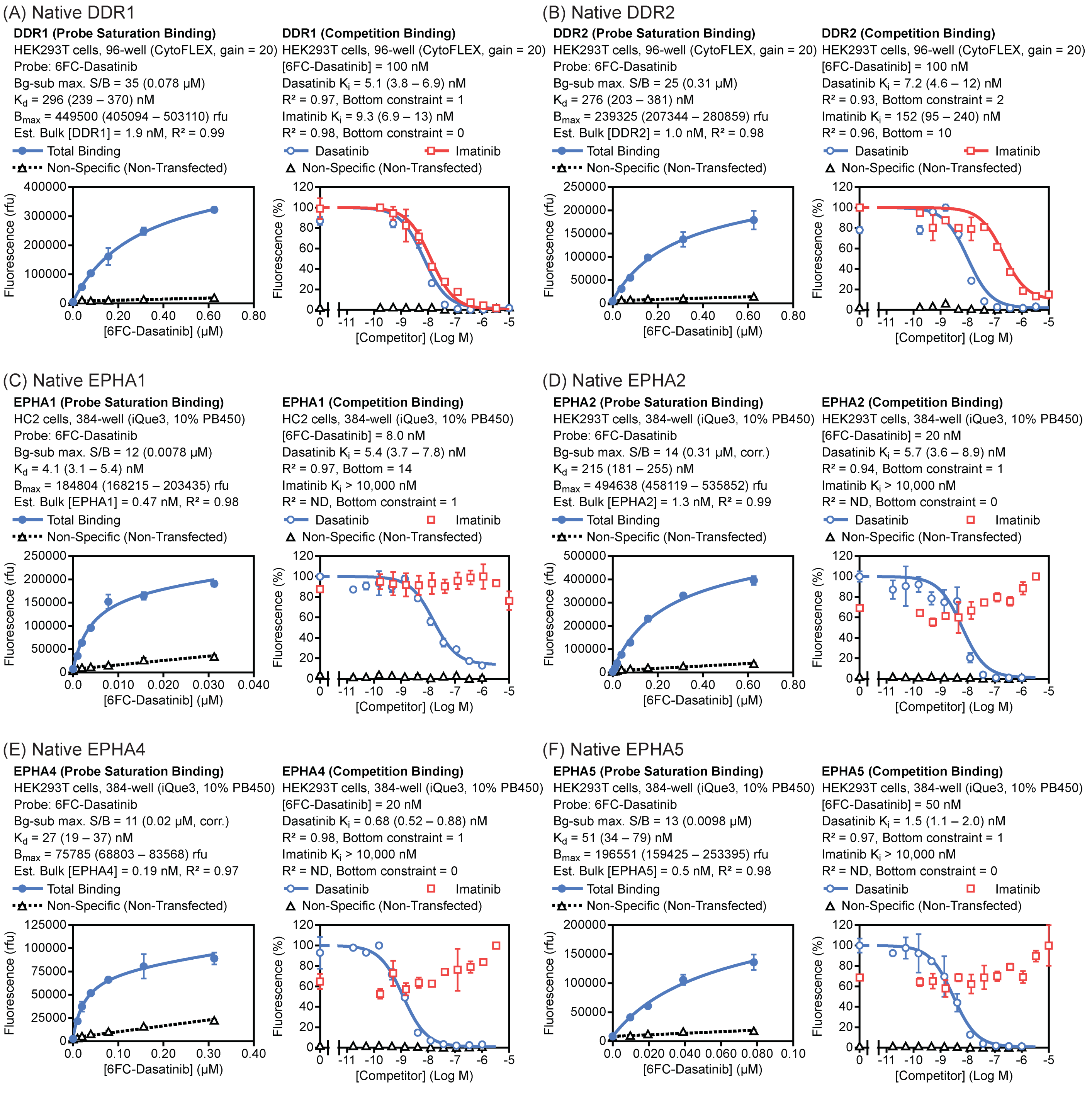

Figure S6. Nonlinear curve fitting for binding of 6FC-Dasatinib (probe **2**, One Site – Total and Nonspecific Binding model) and the competitors dasatinib and imatinib (One Site – Fit Ki model) to native full-length human DDR1, DDR2, EPHA1, EPHA2, EPHA4, and EPHA5 by FPCBA. For curves where Bottom was manually constrained to improve fit stability, Bottom was fixed to the mean fluorescence of that curve's own non-specific-binding wells. Probe Bg-sub max. S/B values labeled "corr." were corrected for a denominator-collapse artifact at non-specific signal <10% of peak. For calculated values, parenthetical numbers represent 95% confidence intervals from curve fitting. ND: no specific binding observed (K_i > 10,000 nM).

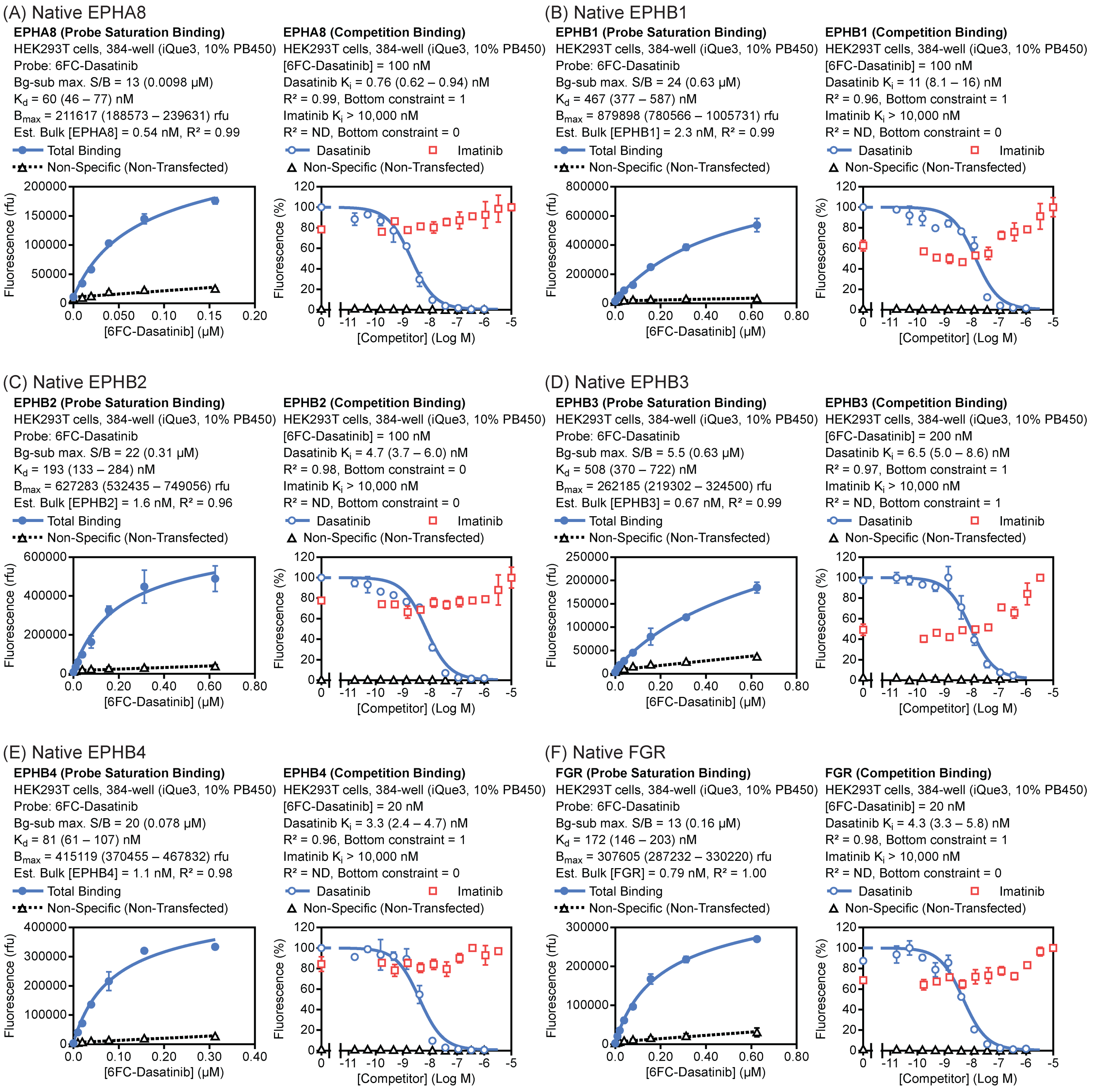

Figure S7. Nonlinear curve fitting for binding of 6FC-Dasatinib (probe **2**, One Site – Total and Nonspecific Binding model) and the competitors dasatinib and imatinib (One Site – Fit Ki model) to native full-length human EPHA8, EPHB1, EPHB2, EPHB3, EPHB4, and FGR by FPCBA. For curves where Bottom was manually constrained to improve fit stability, Bottom was fixed to the mean fluorescence of that curve's own non-specific-binding wells. For calculated values, parenthetical numbers represent 95% confidence intervals from curve fitting. ND: no specific binding observed (K_i > 10,000 nM).

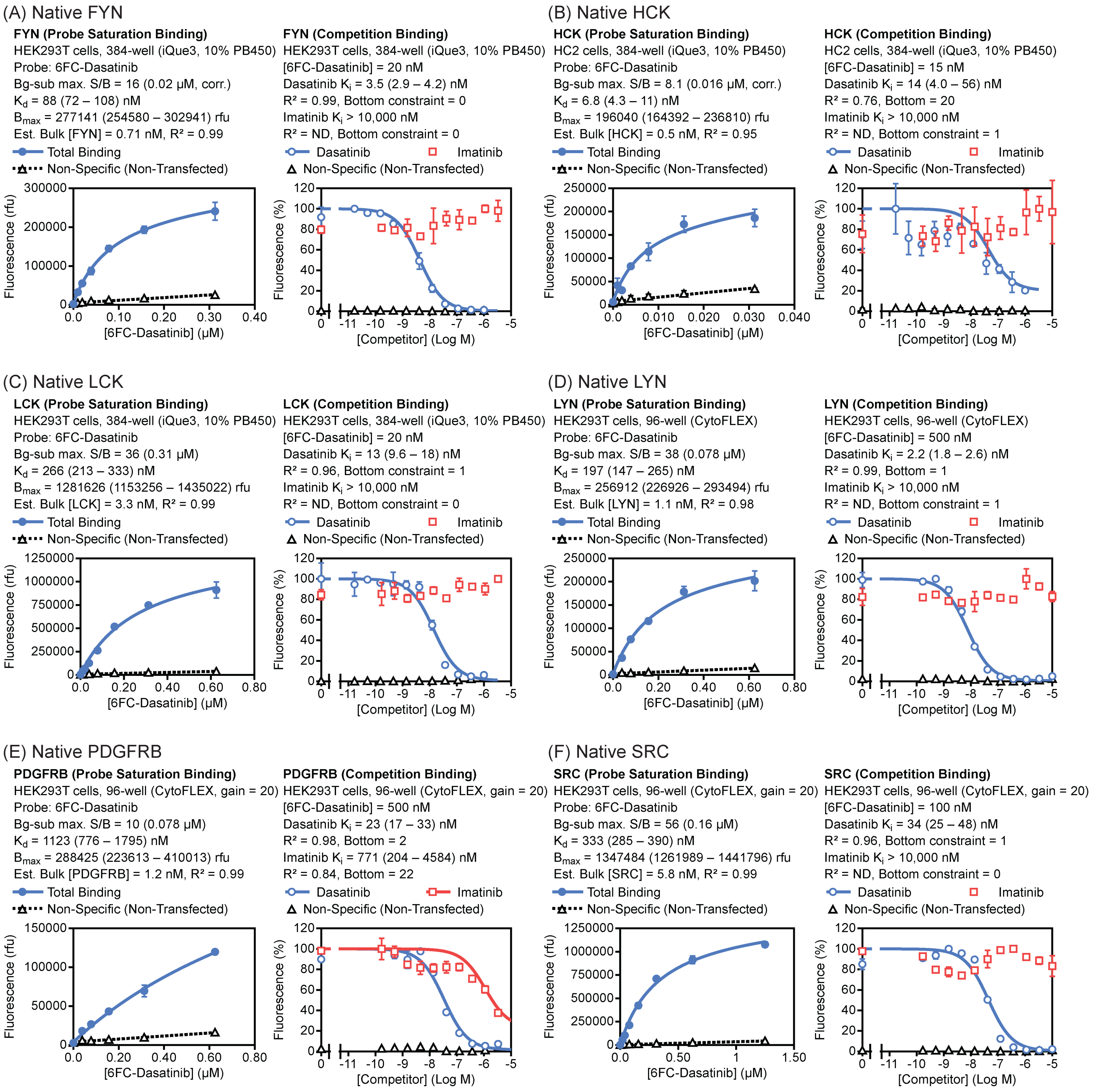

Figure S8. Nonlinear curve fitting for binding of 6FC-Dasatinib (probe **2**, One Site – Total and Nonspecific Binding model) and the competitors dasatinib and imatinib (One Site – Fit Ki model) to native full-length human FYN, HCK, LCK, LYN, PDGFRB, and SRC by FPCBA. For curves where Bottom was manually constrained to improve fit stability, Bottom was fixed to the mean fluorescence of that curve's own non-specific-binding wells. Probe Bg-sub max. S/B values labeled "corr." were corrected for a denominator-collapse artifact at non-specific signal <10% of peak. For calculated values, parenthetical numbers represent 95% confidence intervals from curve fitting. ND: no specific binding observed (K_i > 10,000 nM).

General experimental section for synthesis and compound characterization

Chemicals were purchased from 1ClickChemistry, Combi-Blocks, TCI America, Oakwood Chemical, Ambeed, or MedChemExpress. Nonaqueous reactions were carried out using flame- or oven-dried glassware under an atmosphere of dry argon or nitrogen. Tetrahydrofuran (THF), dichloromethane (CH_2Cl_2), *N,N*-dimethylformamide (DMF), and methanol (CH_3OH) were purified via filtration through two columns of activated basic alumina under an atmosphere of Argon using a solvent purification system from Pure Process Technology. ^{1}H NMR, ^{13}C NMR, and ^{19}F NMR spectra were acquired on a Bruker Avance Neo (400 MHz), Avance III HD (400 MHz) or Bruker Avance III Ascend (700 MHz) instruments. Chemical shifts for ^{1}H, ^{13}C, and ^{19}F (δ) are reported in ppm referenced to $CDCl_3$ (7.26 ppm for ^{1}H and 77.2 ppm for ^{13}C), CD_3OD (3.31 ppm for ^{1}H, 49.0 ppm for ^{13}C), or hexadeuterodimethyl sulfoxide (DMSO-d_6, 2.50 ppm for ^{1}H, 39.5 ppm for ^{13}C). ^{19}F chemical shifts were indirectly referenced to residual solvent ^{1}H peaks (MNova software).[1] NMR data is reported as chemical shift, multiplicity (br = broad, s = singlet, d = doublet, t = triplet, q = quartet, and m = multiplet), coupling constant, and integration. High-resolution mass spectra (HRMS) were obtained at the Mass Spectrometry Laboratory at The Ohio State University College of Pharmacy on a Thermo Q-Exactive Orbitrap with Vanquish-H UHPLC. Thin layer chromatography (TLC) was performed using EMD aluminum-backed (0.20 mm) silica plates (60 F-254). Flash chromatography used ICN silica gel (200−400 mesh). TLC plates were visualized with a UV lamp or by staining with I_2. The purity of fluorescent probes was analyzed using a Waters Acquity UHPLC system with a PDA detector for UV-VIS fitted with a BEH C18 VanGuard FIT (1.7 µm, 2.1 x 50 mm) column and a gradient of H_2O:CH_3CN (95:05) to (05:95) over 4 min followed by H_2O:CH_3CN (95:05) for 1 min at 0.35 mL/min (0.1% formic acid). PB-Gly-Taxol (**7**) was prepared as previously reported.[2] BODIPY-Dasatinib (**8**) was purchased from Promega (Tracer K4) and was confirmed by HRMS to correspond to energy tracer 6 as described by Vasta et al.[3] (calcd for $C_{40}H_{43}BClF_2N_{11}O_3SH^+$: 842.3093; Found: 842.3094).

Synthetic procedures and compound characterization data

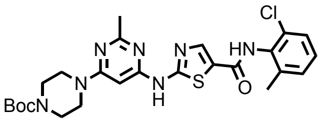

10

tert-Butyl 4-(6-((5-((2-chloro-6-methylphenyl)carbamoyl)thiazol-2-yl)amino)-2-methylpyrimidin-4-yl)piperazine-1-carboxylate (10)

This compound was previously reported[4] but not fully characterized. A mixture of 2-((6-chloro-2-methylpyrimidin-4-yl)amino)-N-(2-chloro-6-methylphenyl)thiazole-5-carboxamide (**9**, Ambeed, 500 mg, 1.27 mmol, 1 equiv.) and *tert*-butyl piperazine-1-carboxylate (472 mg, 2.54 mmol, 2 equiv.) was added to a oven-dried Ar-flushed Biotage microwave vial (5 mL). The vial was sealed and 1.5 mL of anhydrous DMF was added, followed by diisopropylethylamine (DIPEA, 1.12 mL, 6.34 mmol, 5 equiv). The reaction was heated to 110 °C and stirred for 16 h. The crude mixture was dissolved in EtOAc (30 mL) and washed with water (3 x 50 mL). The organic phase was dried over anhydrous $MgSO_4$ and concentrated under reduced pressure to afford a solid. This solid was triturated with cold DCM (10 mL) and filtered to provide **10** (511 mg, 74%), which was used without further purification. ^{1}H NMR (400 MHz, DMSO-d_6) δ 9.95 (s, 1H), 8.31 (s, 1H), 8.25 (s, 1H), 7.47 (dd, *J* = 7.8 Hz, 1H), 7.38 – 7.33 (m, 2H), 6.14 (s, 1H), 3.63 – 3.61 (m, 4H), 3.52 – 3.49 (m, 4H), 2.50 (s, 3H), 2.32 (s, 3H), 1.51 (s, 9H). ^{13}C NMR (100 MHz, DMSO-d_6) δ 165.2, 162.5, 162.3, 159.9, 157.0, 153.9, 140.8, 138.8, 133.5, 132.4, 129.0, 128.2, 127.0, 125.8, 82.8, 79.1, 43.2, 28.1, 25.6, 18.3. HRMS (ESI+) m/z calcd for $C_{25}H_{30}ClN_7O_3SH^+$: 544.1892; Found: 544.1893.

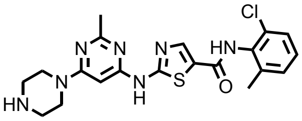

11

N-(2-Chloro-6-methylphenyl)-2-((2-methyl-6-(piperazin-1-yl)pyrimidin-4-yl)amino)thiazole-5-carboxamide (11)

This compound was previously reported.[4] *tert*-Butyl 4-(6-((5-((2-chloro-6-methylphenyl)carbamoyl)thiazol-2-yl)amino)-2-methylpyrimidin-4-yl)piperazine-1-carboxylate (**10**, 514 mg, 0.95 mmol) was dissolved in CH_2Cl_2 / TFA (7:3, 2 mL). After stirring for 1.5 h at room temperature, the reaction mixture was concentrated and diluted with methanol (1 mL). The crude mixture was directly applied to C18 column and purified by reverse phase chromatography with a Buchi C18 column (30 µm spherical; 40 g) on a Teledyne ISCO Combiflash instrument (gradient: H_2O:CH_3CN (90:10) to (0:100) with 0.1% formic acid v/v over 20 min) to afford **11** (226 mg, 54%) as a white solid. ^{1}H NMR (400 MHz, DMSO-d_6) δ 9.96 (s, 1H), 8.30 (s, 1H), 8.25 (s, 1H), 7.46 (dd, J = 7.3, 2.2 Hz, 1H), 7.36 – 7.30 (m, 2H), 6.20 (s, 1H), 3.79 – 3.76 (m, 4H), 3.22 – 3.20 (m, 4H), 2.50 (s, 3H), 2.30 (s, 3H). ^{13}C NMR (100 MHz, DMSO-d_6) δ 165.3, 162.5, 162.1, 159.9, 157.2, 140.8, 138.8, 133.5, 132.4, 129.0, 128.2, 127.0, 125.9, 83.2, 42.6, 41.1, 25.5, 18.3. HRMS (ESI+) m/z calcd for $C_{20}H_{22}ClN_7OSH^+$: 444.1368; Found: 444.1361.

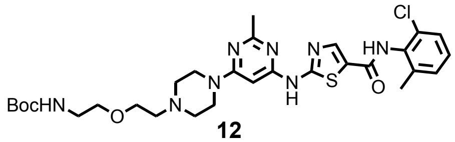

12

tert-Butyl (2-(2-(4-(6-((5-((2-chloro-6-methylphenyl)carbamoyl)thiazol-2-yl)amino)-2-methylpyrimidin-4-yl)piperazin-1-yl)ethoxy)ethyl)carbamate (12)

This compound has been previously reported.[3] *tert*-Butyl *N*-[2-(2-bromoethoxy)ethyl]carbamate (72 mg, 0.27 mmol, 1 equiv.) was added to *N*-(2-chloro-6-methylphenyl)-2-((2-methyl-6-(piperazin-1-yl)pyrimidin-4-yl)amino)thiazole-5-carboxamide (**11**, 119 mg, 0.27 mmol, 1 equiv) in DMF (1.5 mL). Ethylbis(propan-2-yl)amine (DIPEA, 143 µL, 0.81 mmol, 3 equiv.) was added, and the reaction was allowed to stir at 110 °C for 16 h. The mixture was loaded onto a C18 column and purified by reverse phase chromatography with a Buchi C18 column (30 µm spherical; 40 g) on a Teledyne ISCO Combiflash instrument (gradient: H_2O:CH_3CN (90:10) to (0:100) with 0.1% formic acid v/v over 20 min) to yield **12** (88 mg, 52%). ^{1}H NMR (400 MHz, DMSO-d_6) δ 11.47 (s, 1H), 9.87 (s, 1H), 8.22 (s, 1H) 8.13 (s, 1H) (different protonation states observed), 7.40 (dd, J = 7.6, 2.0 Hz, 1H), 7.30 – 7.23

(m, 2H), 6.79 (t, J = 6.0 Hz, 1H), 6.06 (s, 1H), 3.55 (m, 6H), 3.39 (t, J = 6.0 Hz, 3H), 3.08 (q, J = 5.8 Hz, 2H), 2.58 – 2.54 (m, 5H), 2.41 (s, 3H), 2.24 (s, 3H), 1.37 (s, 9H). ^{13}C NMR (100 MHz, DMSO-d_6) δ 165.2, 163.0, 162.6, 159.9, 157.0, 155.6, 140.8, 138.8, 133.5, 132.4, 129.0, 128.2, 127.0, 125.7, 82.7, 77.6, 68.9, 67.5, 56.9, 52.5, 43.3, 39.71, 28.2, 25.6, 18.3. HRMS (ESI+) m/z calculated for $C_{29}H_{39}ClN_8O_4SH^+$: 631.2576; Found: 631.2560.

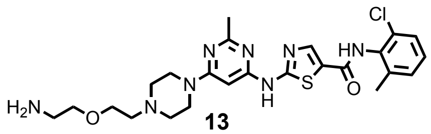

13

2-((6-(4-(2-(2-Aminoethoxy)ethyl)piperazin-1-yl)-2-methylpyrimidin-4-yl)amino)-N-(2-chloro-6-methylphenyl)thiazole-5-carboxamide (13)

This compound has been previously reported.[3] Dichloromethane (0.7 mL) was added to *tert*-butyl(2-(2-(4-(6-((5-((2-chloro-6-methylphenyl)carbamoyl)thiazol-2-yl)amino)-2-methylpyrimidin-4-yl)piperazin-1-yl)ethoxy)ethyl)carbamate (**12**, 88 mg, 0.14 mmol, 1 equiv) in a flask in an ice bath and was allowed to stir for 15 min. Trifluoroacetic acid (0.3 mL) was added to the mixture. The ice bath was removed after 5 min, and the reaction was stirred for 1.5 h. The dichloromethane was removed under vacuum and the crude product was purified by reverse phase chromatography with a Buchi C18 column (30 µm spherical; 40 g) on a Teledyne ISCO Combiflash instrument (gradient: H_2O:CH_3CN (90:10) to (0:100) with 0.1% formic acid v/v over 20 min) to give **13** (49 mg, 66%). ^{1}H NMR (400 MHz, DMSO-d_6) δ 11.70 (s, 1H), 9.96 (s, 1H), 8.30 (s, 1H), 8.00 (bs, 2H), 7.45 (dd, J = 7.6, 2.0 Hz, 1H), 7.36 – 7.29 (m, 2H), 6.23 (s, 1H), 4.36 (s, 1H), 3.85 – 3.82 (m, 2H), 3.71 – 3.68 (m, 2H), 3.43 (m, 9H), 3.11 – 3.08 (m, 2H), 2.50 (s, 3H), 2.29 (s, 3H). ^{13}C NMR (175 MHz, DMSO-d_6) δ 165.4, 162.4, 162.0, 159.9, 158.5, 158.2, 157.2, 140.8, 138.8, 133.5, 132.4, 129.0, 128.2, 127.0, 126.0, 83.3, 66.6, 55.1, 50.8, 38.4, 25.5, 18.3. HRMS (ESI+) m/z calcd for $C_{24}H_{31}ClN_8O_2SH^+$: 531.2052; Found: 531.2048.

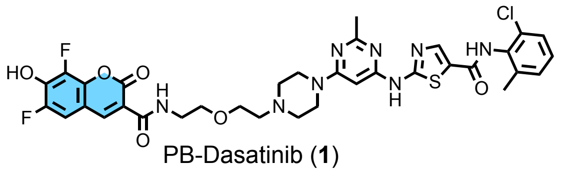
PB-Dasatinib (**1**)

N-(2-chloro-6-methylphenyl)-2-((6-(4-(2-(2-(6,8-difluoro-7-hydroxy-2-oxo-2H-chromene-3-carboxamido)ethoxy)ethyl)piperazin-1-yl)-2-methylpyrimidin-4-yl)amino)thiazole-5-carboxamide (PB-Dasatinib, 1)

To 2-((6-(4-(2-(2-aminoethoxy)ethyl)piperazin-1-yl)-2-methylpyrimidin-4-yl)amino)-N-(2-chloro-6-methylphenyl)thiazole-5-carboxamide (**13**, 23 mg, 0.043 mmol, 1 equiv.) in DMF (1 mL) was added Pacific Blue-NHS ester, prepared as previously described[5, 6] (14.7 mg, 0.043 mmol, 1 equiv.) and DIPEA (19.2 µL, 0.108 mmol, 2.5 equiv.). An argon balloon was added, and the reaction stirred at room temperature (22 °C) for 16 h. The reaction was purified by reverse phase chromatography with a Buchi C18 column (30 µm spherical; 40 g) on a Teledyne ISCO Combiflash instrument (gradient: H_2O:CH_3CN (90:10) to (0:100) with 0.1% formic acid v/v over 20 min) to yield **1** as a yellow solid (15 mg, 47%). ^{1}H NMR (700 MHz, DMSO-d_6) δ 9.89 (s, 1H), 8.75 (t, J = 5.6 Hz, 1H), 8.47 (s, 1H), 8.25 (s, 1H), 8.23 (s, 1H), 7.40 (d, J = 7.0 Hz, 1H), 7.30 – 7.29 (m, 1H), 7.26 (t, J = 8.0 Hz, 1H), 7.19 (d, J = 11.2 Hz, 1H), 6.06 (s, 1H), 3.58 (t, J = 6.0 Hz, 4H), 3.53 – 3.45 (m, 5H), 3.46 (q, J = 5.6 Hz, 4H), 2.55 – 2.54 (m, 4H), 2.40 (s, 3H), 2.25 (s, 3H). ^{13}C NMR (175 MHz, DMSO-d_6) δ 165.6, 164.7, 163.7, 163.0, 162.8, 162.2, 160.4, 159.4 (dd, $^2J_{C\text{-}F}$ = 19.4, 14.2 Hz), 157.4, 154.4 (dd, $^1J_{C\text{-}F}$ = 238.6, 9.0 Hz), 147.7 (t, $^4J_{C\text{-}F}$ = 3.9 Hz), 143.5 (d, J = 7.7 Hz), 141.8 (dd, $^1J_{C\text{-}F}$ = 233.4, 10.9 Hz), 141.3, 139.3, 134.0, 132.9, 129.5, 128.6, 127.5, 126.1, 109.3 (d, $^2J_{C\text{-}F}$ = 20.6 Hz), 102.4, 99.6 (d, $^3J_{C\text{-}F}$ = 11.0 Hz), 83.1, 69.6, 68.7, 57.6, 53.2, 44.0, 39.1, 34.8, 26.0, 18.8. ^{19}F NMR (376 MHz, DMSO-d_6) δ = -135.05 (t, J = 10.3 Hz), -154.20 (d, J = 10.6 Hz). HRMS (ESI+) m/z calcd for $C_{34}H_{33}ClF_2N_8O_6SH^+$: 755.1973; Found: 755.1967.

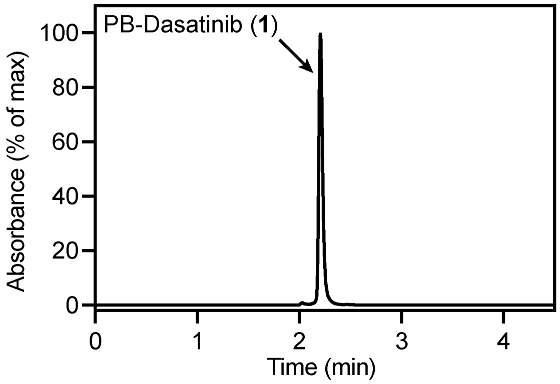

Figure S9. Analytical HPLC profile of purified probe **1**. Retention time = 2.2 min monitored by absorbance at 254 nm. Purity by HPLC > 99%.

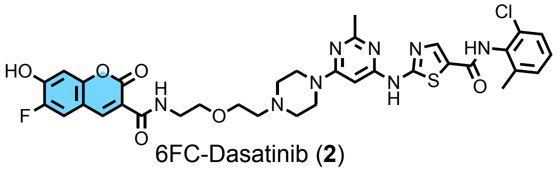
6FC-Dasatinib (**2**)

N-(2-chloro-6-methylphenyl)-2-((6-(4-(2-(2-(6-fluoro-7-hydroxy-2-oxo-2H-chromene-3-carboxamido)ethoxy)ethyl)piperazin-1-yl)-2-methylpyrimidin-4-yl)amino)thiazole-5-carboxamide (6FC-Dasatinib, 2)

To 2-((6-(4-(2-(2-aminoethoxy)ethyl)piperazin-1-yl)-2-methylpyrimidin-4-yl)amino)-N-(2-chloro-6-methylphenyl)thiazole-5-carboxamide (**13**, 49 mg, 0.093 mol, 1 equiv.) in DMF (2.5 mL) was added 2,5-dioxopyrrolidin-1-yl 6-fluoro-7-hydroxy-2-oxo-2H-chromene-3-carboxylate (29.8 mg, 0.093 mmol, 1 equiv.) and DIPEA (41 µL, 0.232 mmol, 2.5 equiv.). An argon balloon was added, and the reaction stirred at room temperature (22 °C) for 16 h. The reaction was purified by reverse phase chromatography with a Buchi C18 column (30 µm spherical; 40 g) on a Teledyne ISCO Combiflash instrument (gradient: H_2O:CH_3CN (90:10) to (0:100) with 0.1% formic acid v/v over 20 min) to yield **2** as a yellow solid (44 mg, 65%) ^1H NMR (400 MHz, DMSO-d_6) δ 11.55 (s, 1H), 9.97 (s, 1H), 8.90 (t, J = 5.2 Hz, 1H), 8.87 (s, 1H), 8.33 (s, 1H), 8.24 (s, 1H), 7.89 (d, J = 10.8 Hz, 1H), 7.50 (dd, J = 7.4, 2.2 Hz, 1H), 7.41 – 7.34 (m, 2H), 6.99 (d, J = 7.6 Hz, 1H), 6.15 (s, 1H), 3.71 (t, J = 5.4 Hz, 2H), 3.68 – 3.60 (m, 8H), 2.73 – 2.67 (m, 6H), 2.50 (s, 3H), 2.35 (s, 3H). ^{13}C NMR (175 MHz, DMSO-d_6)

δ 165.1, 163.0, 162.5, 162.3, 161.5, 161.0, 159.9, 156.9, 153.6, 152.6, 149.1 (d, $^1J_{C\text{-}F}$ = 240.0 Hz), 147.5, 140.8, 138.8, 133.5, 132.4, 129.0, 128.1, 127.0, 125.7, 115.4 (d, $^2J_{C\text{-}F}$ = 20.6 Hz), 113.4, 109.5 (d, $^3J_{C\text{-}F}$ = 9.0 Hz), 104.1 (d, $^4J_{C\text{-}F}$ = 3.2 Hz), 82.7, 68.5, 67.9, 56.9, 52.5, 43.3, 25.5, 18.3. ^{19}F NMR (376 MHz, DMSO-d_6) δ = -138.33. HRMS (ESI+) m/z calcd for $C_{34}H_{34}ClFN_8O_6SH^+$: 737.2067; Found: 737.2059.

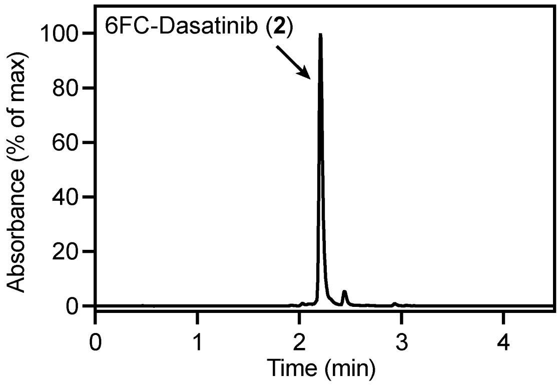

Figure S10. Analytical HPLC profile of purified probe **2**. Retention time = 2.2 min monitored by absorbance at 254 nm. Purity by HPLC > 99%.

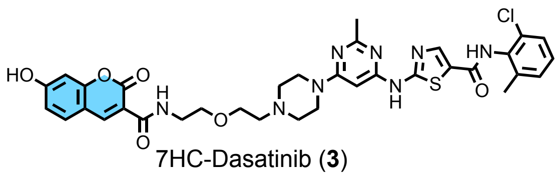

7HC-Dasatinib (**3**)

***N*-(2-Chloro-6-methylphenyl)-2-((6-(4-(2-(2-(7-hydroxy-2-oxo-2*H*-chromene-3-carboxamido)ethoxy)ethyl)piperazin-1-yl)-2-methylpyrimidin-4-yl)amino)thiazole-5-carboxamide (7HC-Dasatinib, 3)**

To 2-((6-(4-(2-(2-aminoethoxy)ethyl)piperazin-1-yl)-2-methylpyrimidin-4-yl)amino)-N-(2-chloro-6-methylphenyl)thiazole-5-carboxamide (**13**, 13.7 mg, 0.0258 mmol, 1 equiv.) in DMF (2.5 mL) was added 7-hydroxycoumarin-3-carboxylic acid *N*-succinimidyl ester (7.82 mg, 0.0258 mmol, 1 equiv.) and DIPEA (11.4 µL, 0.0645 mmol, 2.5 equiv.). An argon balloon was added, and the reaction stirred at room temperature (22 °C) for 16 h. The reaction was purified by reverse phase chromatography with a Buchi C18 column (30 µm spherical; 40 g) on a Teledyne ISCO Combiflash instrument

(gradient: H_2O:CH_3CN (90:10) to (0:100) with 0.1% formic acid v/v over 20 min) to yield **3** as a yellow solid. (8.4 mg, 45%) ^{1}H NMR (400 MHz, DMSO-d_6) δ 11.44 (s, 1H), 9.86 (s, 1H), 8.81 (s, 1H), 8.79 (t, *J* = 5.4 Hz, 1H), 8.22 (s, 1H), 7.82 (d, *J* = 8.8 Hz, 1H), 7.40 (dd, *J* = 7.6, 2.0 Hz, 1H), 7.28 – 7.25 (m, 2H), 6.87 (dd, *J* = 8.8, 2.4 Hz, 1H), 6.80 (d, *J* = 2.4 Hz, 1H), 6.04 (s, 1H), 3.59 (t, *J* = 5.6 Hz, 2H), 3.54 (d, *J* = 4.6 Hz, 2H), 3.50 (d, *J* = 5.6 Hz, 6H), 2.55 (t, *J* = 5.6 Hz, 6H), 2.39 (s, 3H), 2.24 (s, 3H). ^{13}C NMR (175 MHz, DMSO-d_6) δ 165.1, 163.7, 162.6, 162.4, 162.3, 161.5, 161.1, 159.9, 156.9, 156.3, 148.2, 140.8, 138.8, 133.5, 132.4, 132.0, 129.0, 128.1, 127.0, 125.7, 114.4, 113.3, 111.1, 101.8, 82.6, 68.7, 68.2, 57.1, 52.6, 43.6, 25.5, 18.3. HRMS (ESI+) m/z calcd for $C_{34}H_{35}ClN_8O_6SH^+$: 719.2162; Found: 719.2156

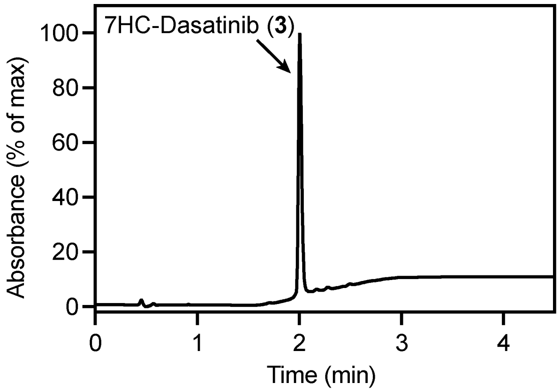

Figure S11. Analytical HPLC profile of purified probe **3**. Retention time = 2.0 min monitored by absorbance at 254 nm. Purity by HPLC > 99%.

General procedure 1: Synthesis of fluorescent probes 4–6 by reaction of amines with fluorophore NHS esters. An oven dried Ar flushed Biotage microwave reaction vial (5 mL) equipped with a magnetic stir bar was charged with the primary amine (1 equiv.) and fluorophore NHS ester (1.1 equiv.). Anhydrous DMF (1.5 mL) was added and the mixture was treated with ethylbis(propan-2-yl)amine (DIPEA, 4 equiv.). The reaction mixture was stirred at 22 °C for 16 h. The mixture was purified by reverse phase preparative high performance liquid chromatography (HPLC, PRP-1 reverse phase column, H_2O and CH_3CN, 0.1% formic acid v/v, gradient: H_2O:CH_3CN (90:10) to (0:100) over 30 min). Pure fractions were collected and combined, and the

solvent was removed using lyophilization for 16 h to afford probes derived from the PB, 6FC, and 7HC fluorophores.

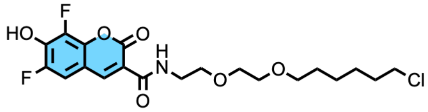

PB-HaloTag ligand (**4**)

N-(2-(2-((6-chlorohexyl)oxy)ethoxy)ethyl)-6,8-difluoro-7-hydroxy-2-oxo-2*H*-chromene-3-carboxamide (4). Following general procedure 1, 2-(2-((6-chlorohexyl)oxy)ethoxy)ethylamine hydrochloride (**14**, 25 mg, 0.096 mmol, 1 equiv.) and Pacific Blue-NHS ester (36 mg, 0.106 mmol, 1.1 equiv.) afforded **4** as a yellow powder (36 mg, 84%). ^{1}H NMR (700 MHz, DMSO-d_6) δ 8.80 (s, 1H), 8.70 (t, J = 5.4 Hz, 1H), 7.73 (dd, J = 10.6, 2.2 Hz, 1H), 3.60 – 3.54 (m, 6H), 3.50 – 3.48 (m, 4H), 3.38 (t, J = 6.6 Hz, 2H), 1.67 (p, J = 6.8 Hz, 2H), 1.47 (p, J = 6.9 Hz, 2H), 1.38 – 1.26 (m, 4H). ^{13}C NMR (175 MHz, DMSO-d_6) δ 161.5, 160.2, 149.5 (dd, $^1J_{C\text{-}F}$ = 239.6, 4.9 Hz), 147.9, 141.2 (d, $^4J_{C\text{-}F}$ = 3.9 Hz), 141.1 (d, $^3J_{C\text{-}F}$ = 9.1 Hz), 139.3 (dd, $^1J_{C\text{-}F}$ = 243.4, 6.8 Hz), 116.0, 111.0 (dd, $^2J_{C\text{-}F}$ = 21.1, 2.9 Hz), 109.6 (d, $^3J_{C\text{-}F}$ = 10.3 Hz), 70.7, 70.2, 69.9, 69.2, 45.8, 39.5, 32.5, 29.6, 26.6, 25.4. ^{19}F NMR (376 MHz, DMSO-d_6) δ -135.05 (t, J = 10.3 Hz), -154.21 (d, J = 10.5 Hz). HRMS (ESI+) m/z calcd for $C_{20}H_{24}ClF_2NO_6H^+$: 448.1333; Found: 448.1327.

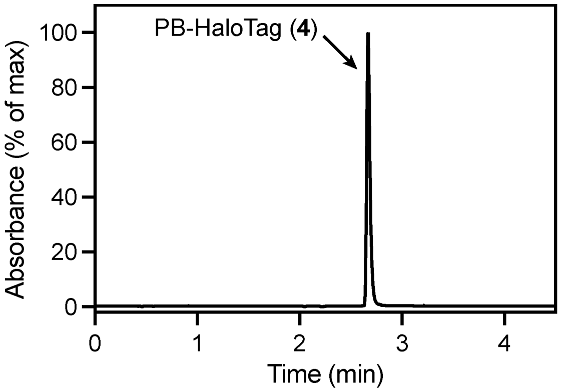

Figure S12. Analytical HPLC profile of purified probe **4**. Retention time = 2.7 min monitored by absorbance at 254 nm. Purity by HPLC > 99%.

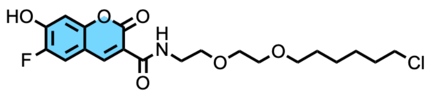

6FC-HaloTag ligand (**5**)

N-(2-(2-((6-chlorohexyl)oxy)ethoxy)ethyl)-6-fluoro-7-hydroxy-2-oxo-2*H*-chromene-3-carboxamide (5). Following general procedure 1, 2-(2-((6-chlorohexyl)oxy)ethoxy)ethylamine hydrochloride (**14**, 25 mg, 0.096 mmol, 1 equiv.) and 2,5-dioxopyrrolidin-1-yl 6-fluoro-7-hydroxy-2-oxo-2*H*-chromene-3-carboxylate (34 mg, 0.106 mmol, 1.1 equiv.) afforded **5** as an off-white powder (32 mg, 78%). ^{1}H NMR (700 MHz, DMSO-d_6) δ 8.79 (s, 1H), 8.77 (t, *J* = 5.6 Hz, 1H), 7.84 (d, *J* = 11.2 Hz, 1H), 6.95 (d, *J* = 7.7 Hz, 1H), 3.59 (t, *J* = 6.6 Hz, 2H), 3.55 (t, *J* = 4.9 Hz, 4H), 3.50 – 3.47 (m, 4H), 3.38 (t, *J* = 7.0 Hz, 2H), 1.67 (p, *J* = 7.0 Hz, 2H), 1.47 (p, *J* = 7.0 Hz, 2H), 1.34 (p, *J* = 7.5 Hz, 2H), 1.28 (p, *J* = 7.5 Hz, 2H). ^{13}C NMR (175 MHz, DMSO-d_6) 161.8, 161.3, 152.8 (d, *J* = 14.2 Hz), 152.7, 149.2 (d, *J* = 240.6 Hz), 148.1 (d, *J* = 2.4 Hz), 116.1 (d, *J* = 20.6 Hz), 114.8, 110.5 (d, *J* = 8.4 Hz), 104.5, 70.7, 70.2, 69.9, 69.3, 45.8, 39.5, 32.5, 29.6, 26.6, 25.4. ^{19}F NMR (376 MHz, DMSO-d_6) δ -138.38. HRMS (ESI+) m/z calcd for $C_{20}H_{25}ClFNO_6H^+$: 430.1427; Found: 430.1419.

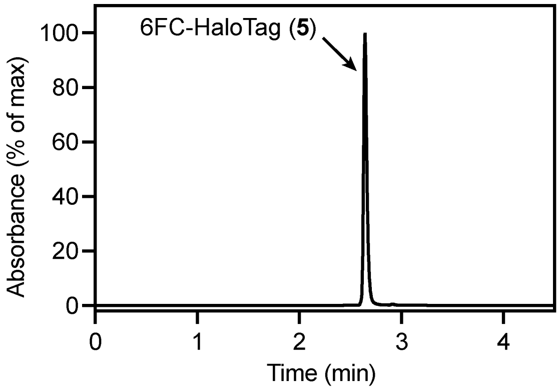

Figure S13. Analytical HPLC profile of purified probe **5**. Retention time = 2.6 min monitored by absorbance at 254 nm. Purity by HPLC > 99%

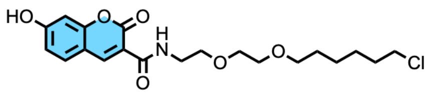

7HC-HaloTag ligand (**6**)

N-(2-(2-((6-chlorohexyl)oxy)ethoxy)ethyl)-7-hydroxy-2-oxo-2*H*-chromene-3-carboxamide (6). Following general procedure 1, 2-(2-((6-chlorohexyl)oxy)ethoxy)ethylamine hydrochloride (**14**, 25 mg, 0.096 mmol, 1 equiv.) and 2,5-dioxopyrrolidin-1-yl 7-hydroxy-2-oxo-2H-chromene-3-carboxylate (32 mg, 0.106 mmol, 1.1 equiv.) afforded **6** as an off-white powder (24 mg, 60%). ^{1}H NMR (400 MHz, DMSO-d_6) δ 8.80 (s, 1H), 8.78 (t, J = 5.6 Hz, 1H), 7.81 (d, J = 8.4 Hz, 1H), 6.87 (dd, J = 8.4, 2.1 Hz, 1H), 6.79 (d, J = 2.1 Hz, 1H), 3.58 (t, J = 7.0 Hz, 2H), 3.55 (t, J = 4.9 Hz, 4H), 3.50 – 3.47 (m, 4H), 3.38 (t, J = 6.5 Hz, 2H), 1.67 (p, J = 7.0 Hz, 2H), 1.47 (p, J = 7.0 Hz, 2H), 1.34 (p, J = 7.4 Hz, 1H), 1.28 (q, J = 7.4 Hz, 1H). ^{13}C NMR (175 MHz, DMSO-d_6) δ 164.5, 162.0, 161.6, 156.8, 148.6, 132.5, 114.9, 113.7, 111.5, 102.3, 70.7, 70.2, 70.0, 69.3, 45.8, 39.4, 32.5, 29.6, 26.6, 25.4. HRMS (ESI+) m/z calcd for $C_{20}H_{26}ClNO_6H^+$: 412.1521; Found: 412.1511.

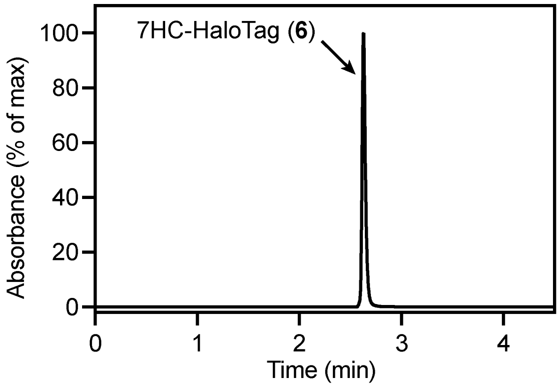

Figure S14. Analytical HPLC profile of purified probe **6**. Retention time = 2.6 min monitored by absorbance at 254 nm. Purity by HPLC > 99%.

NMR Spectra

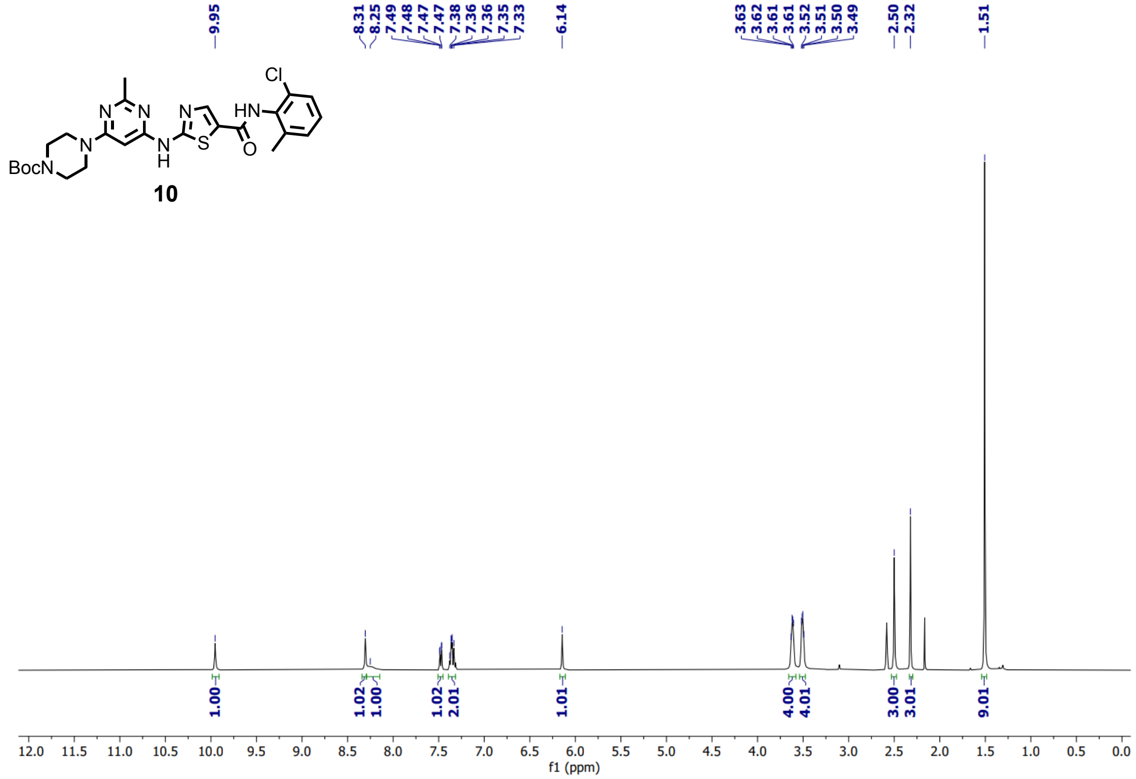

Figure S15. ^{1}H NMR (400 MHz, DMSO-d_6) spectrum of **10**.

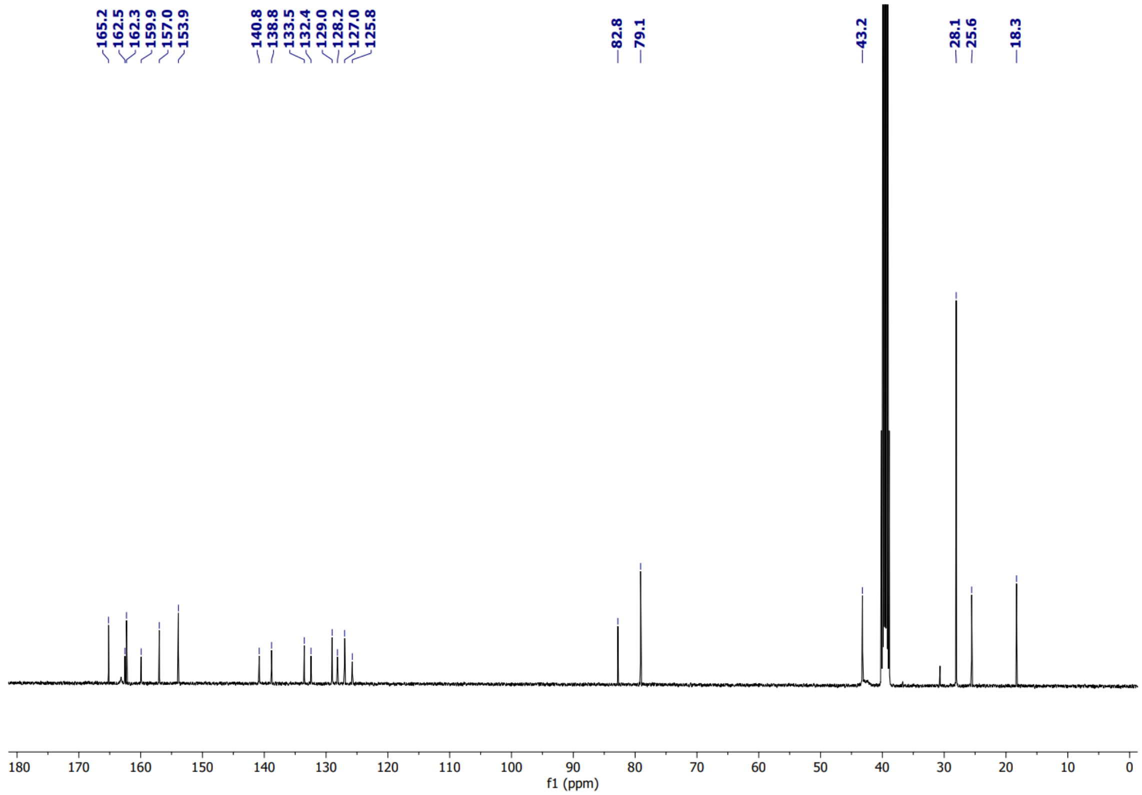

Figure S16. ^{13}C NMR (100 MHz, DMSO-d_6) spectrum of **10**.

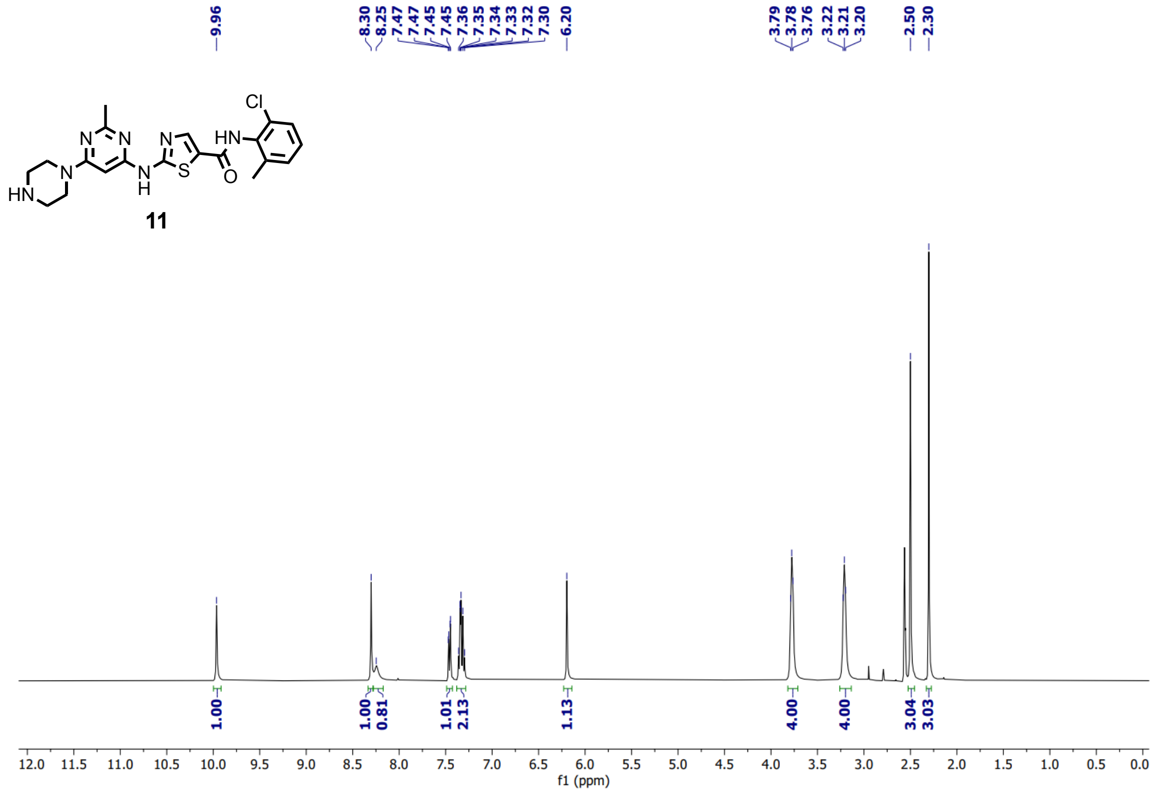

Figure S17. ^{1}H NMR (400 MHz, DMSO-d_6) spectrum of **11**.

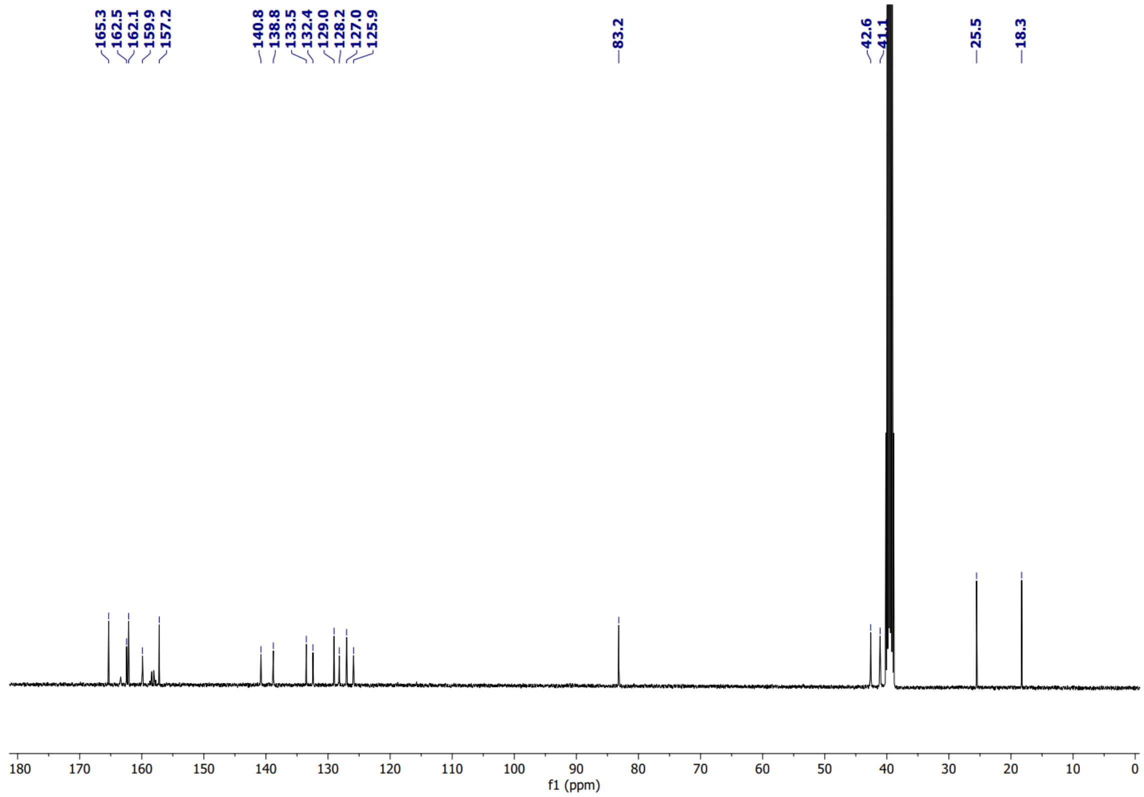

Figure S18. ^{13}C NMR (100 MHz, DMSO-d_6) spectrum of **11**.

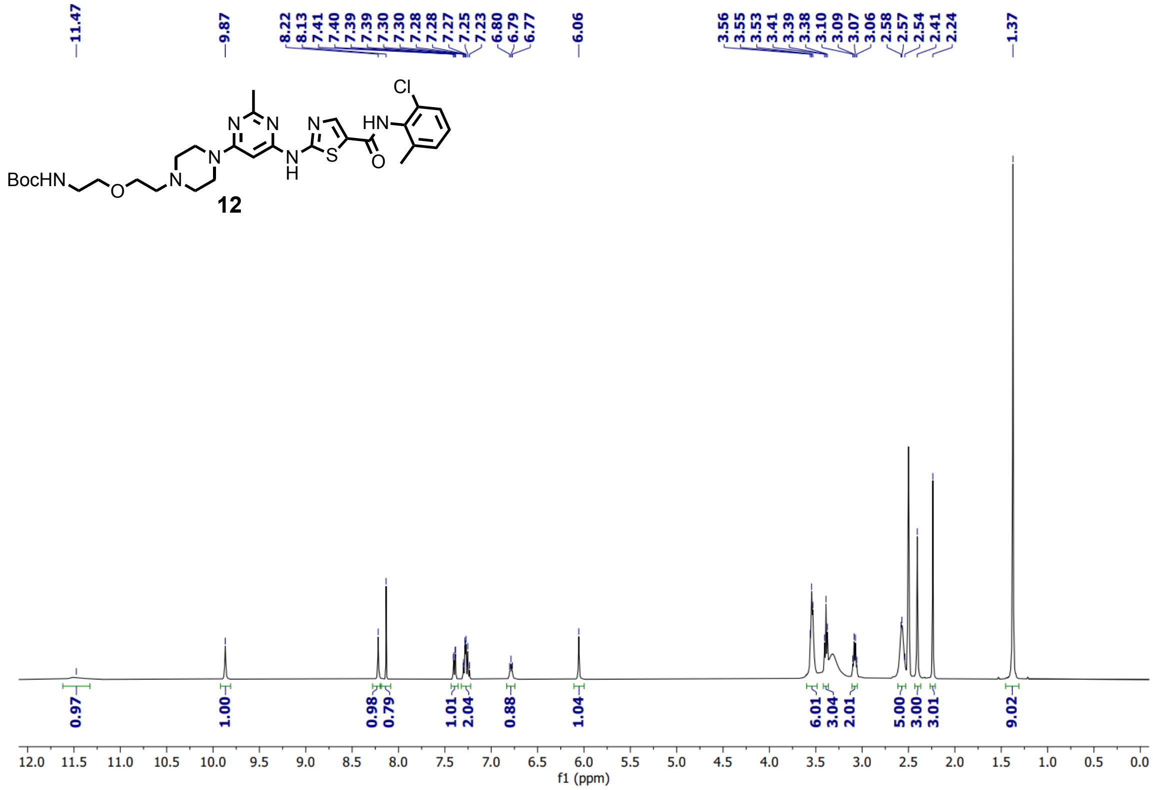

Figure S19. ^{1}H NMR (400 MHz, DMSO-d_6) spectrum of **12**.

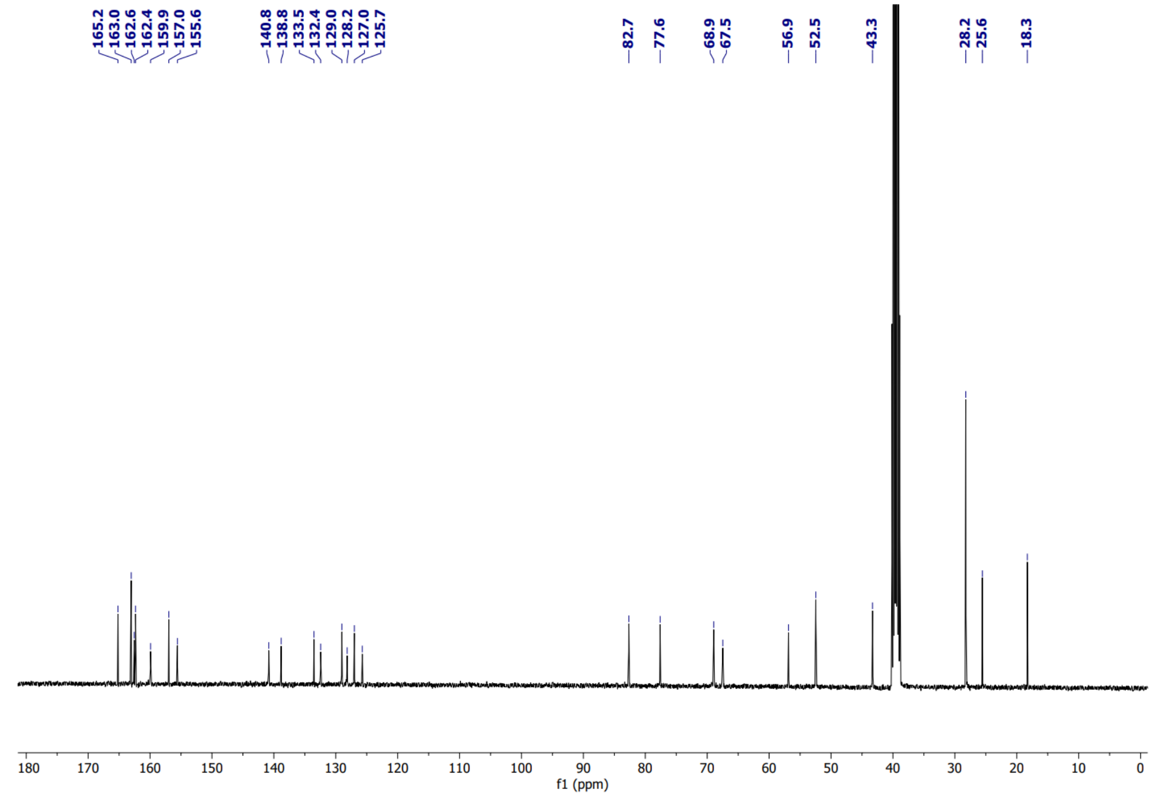

Figure S20. ^{13}C NMR (100 MHz, DMSO-d_6) spectrum of **12**.

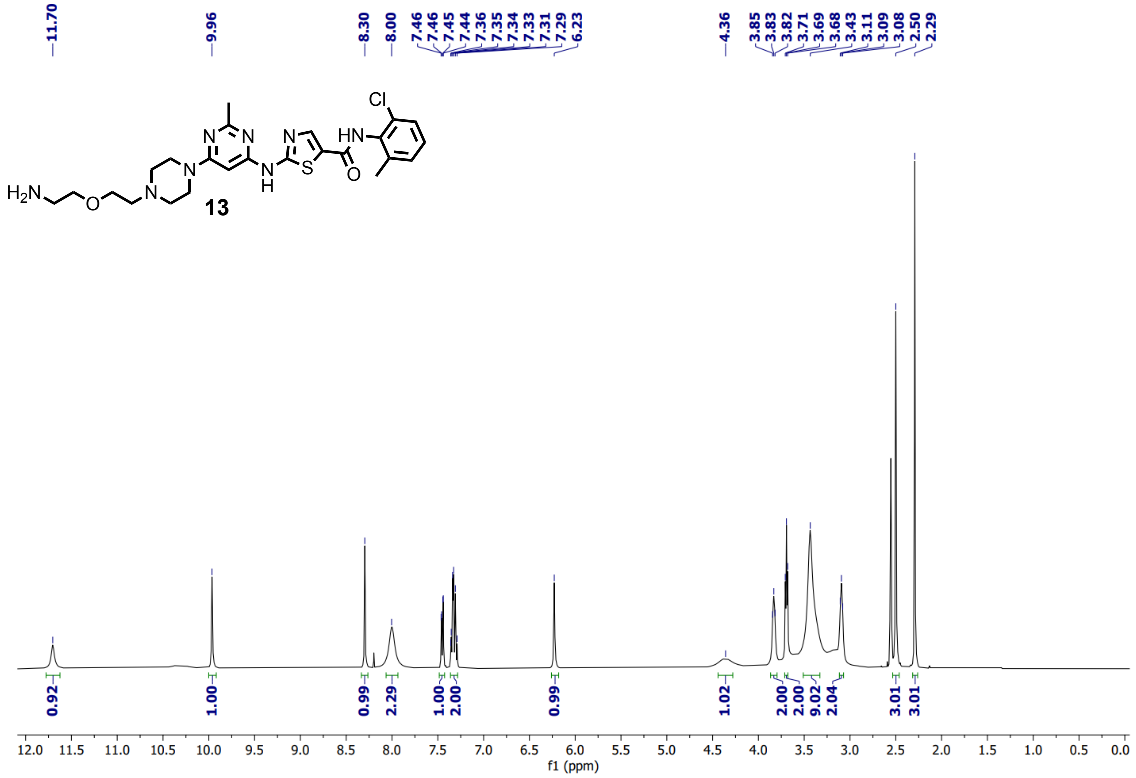

Figure S21. ^{1}H NMR (400 MHz, DMSO-d_6) spectrum of **13**.

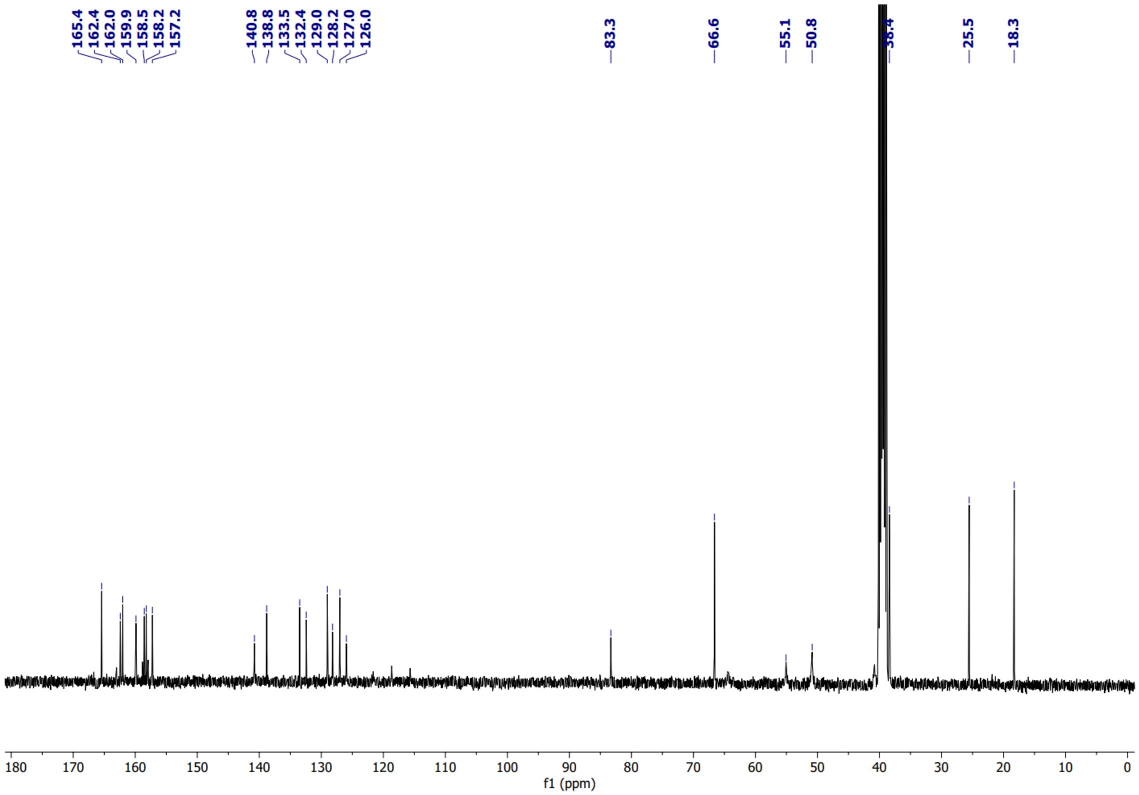

Figure S22. ^{13}C NMR (100 MHz, DMSO-d_6) spectrum of **13**.

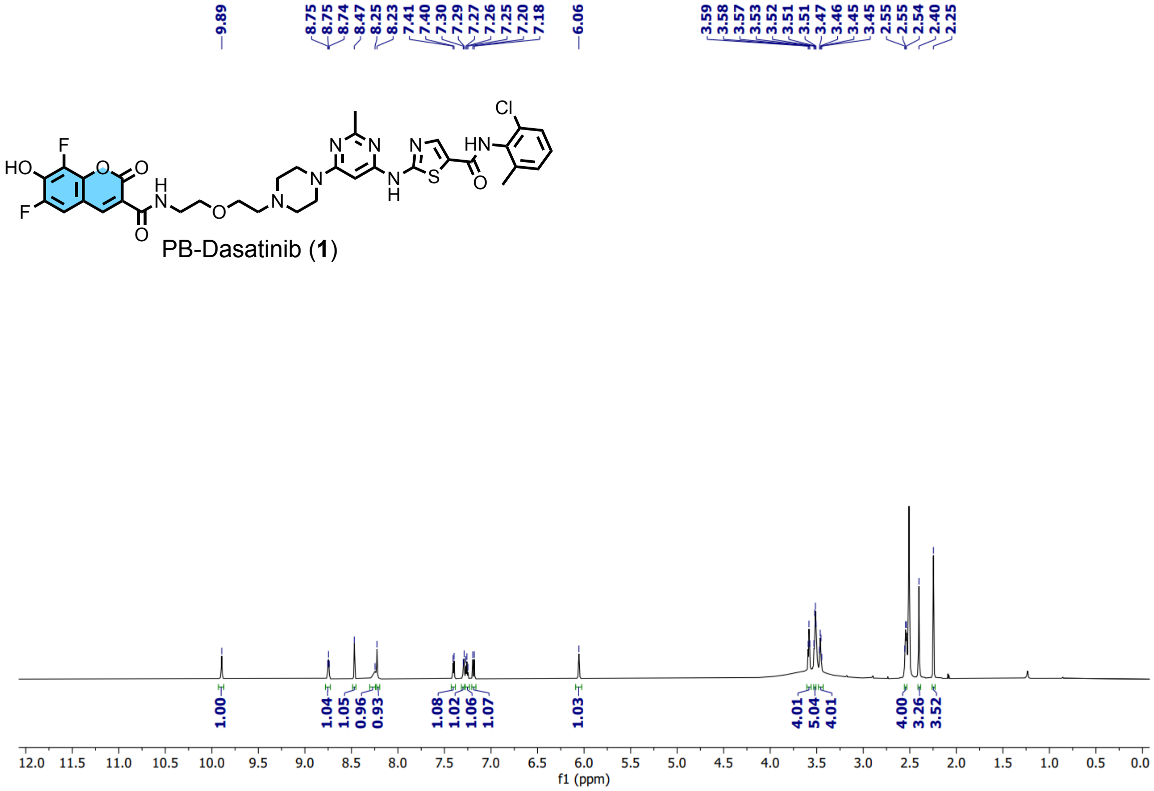

Figure S23. ^{1}H NMR (700 MHz, DMSO-d_6) spectrum of **1**.

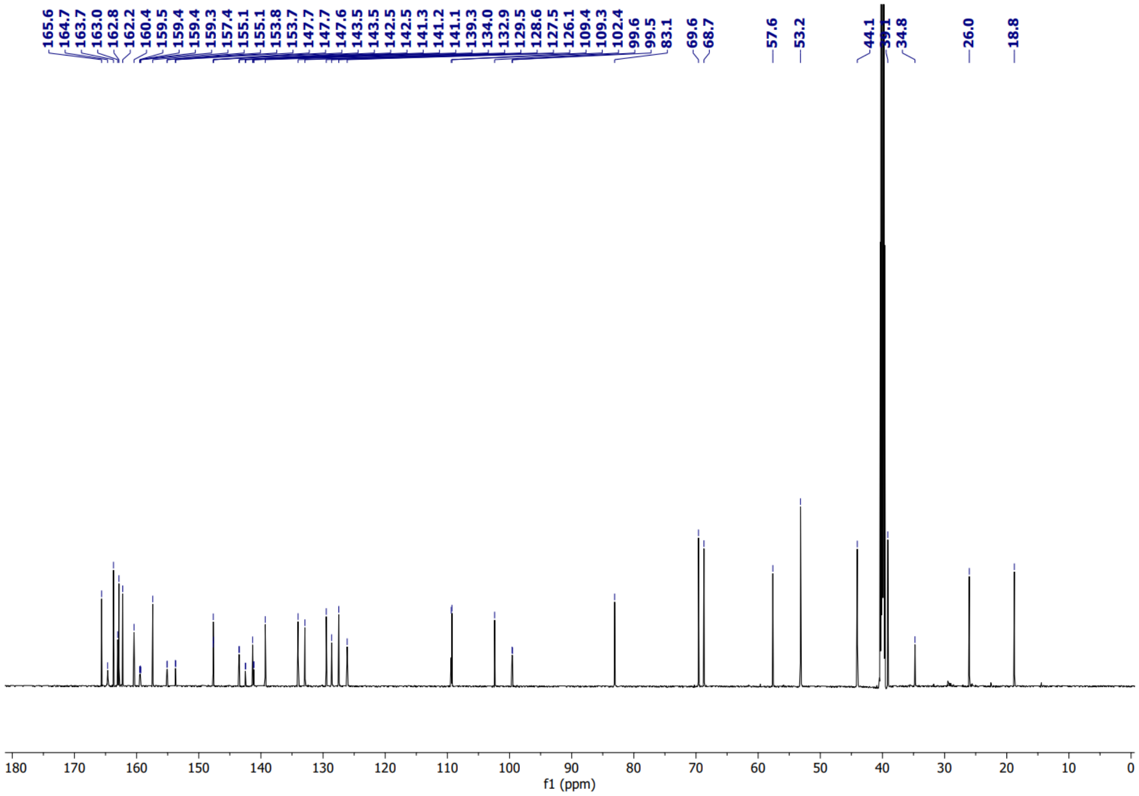

Figure S24. ^{13}C NMR (175 MHz, DMSO-d_6) spectrum of **1**.

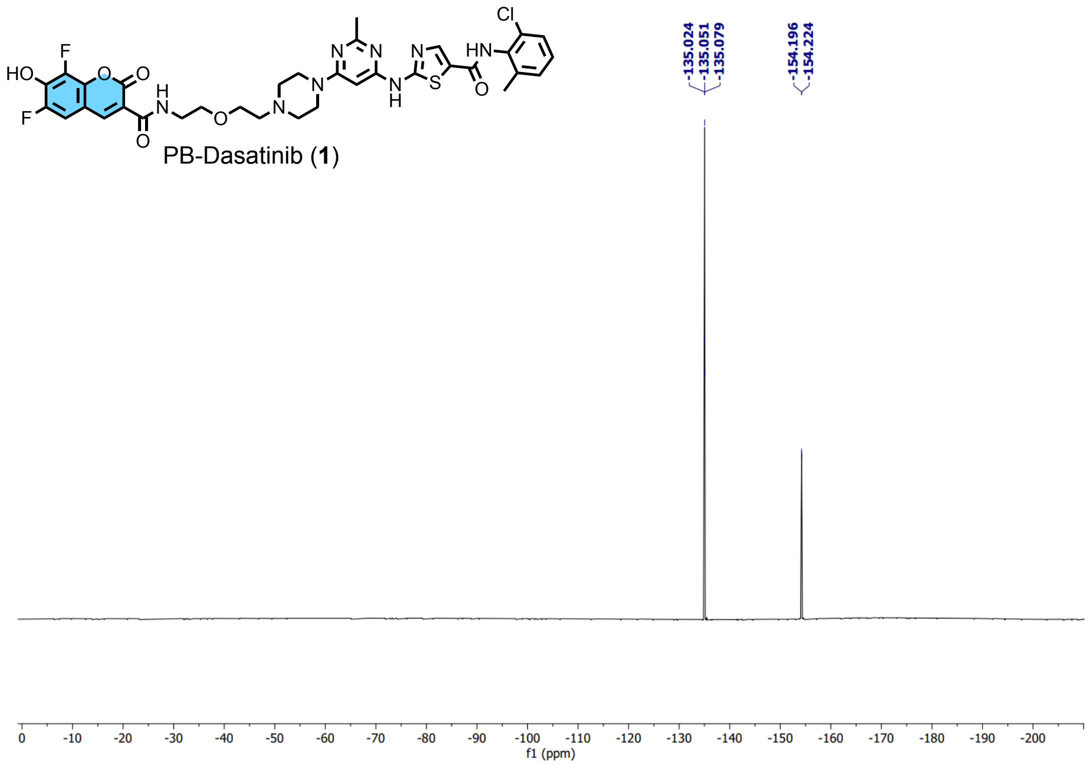

Figure S25. ^{19}F NMR (377 MHz, DMSO-d_6) spectrum of **1**.

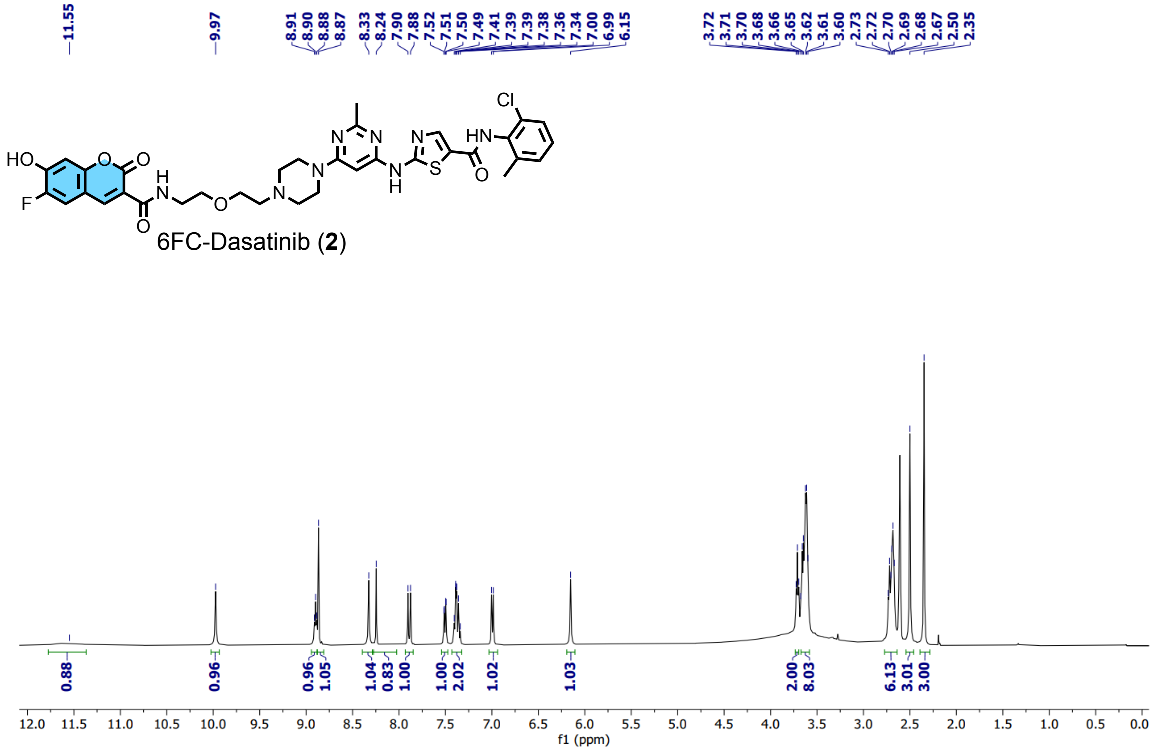

Figure S26. ^{1}H NMR (700 MHz, DMSO-d_6) spectrum of **2**.

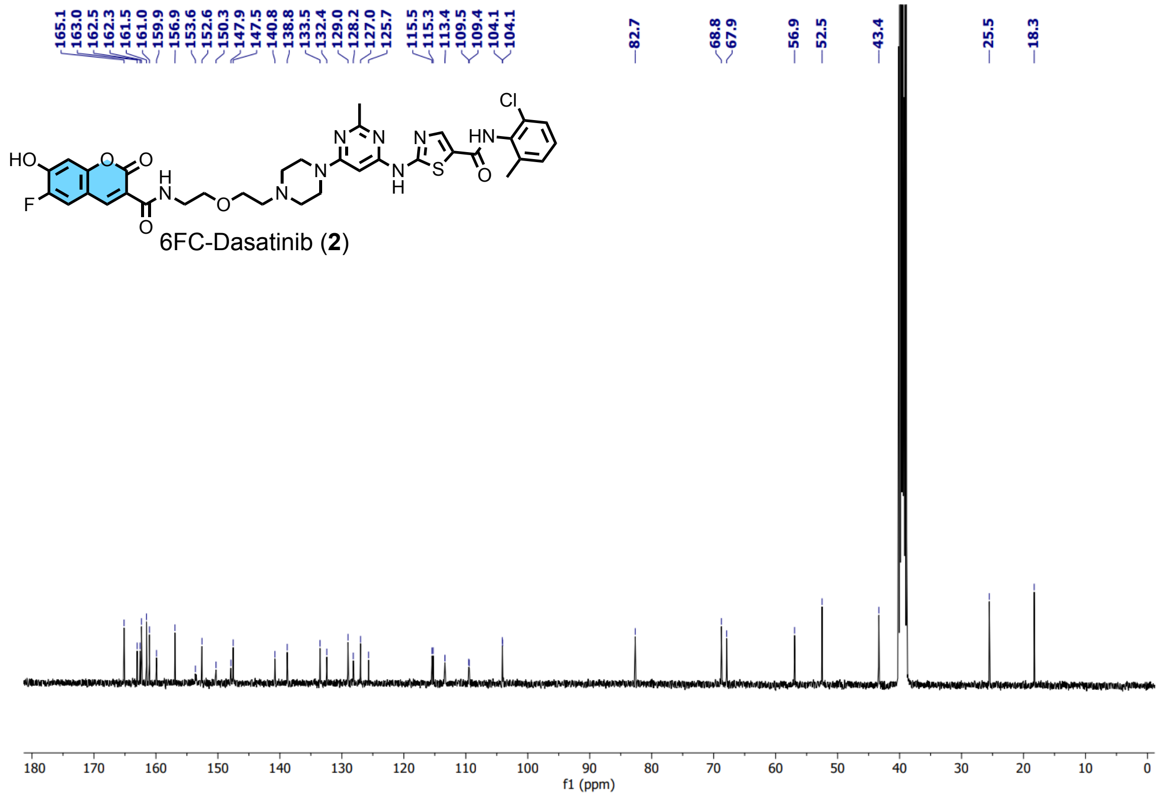

Figure S27. ^{13}C NMR (175 MHz, DMSO-d_6) spectrum of **2**.

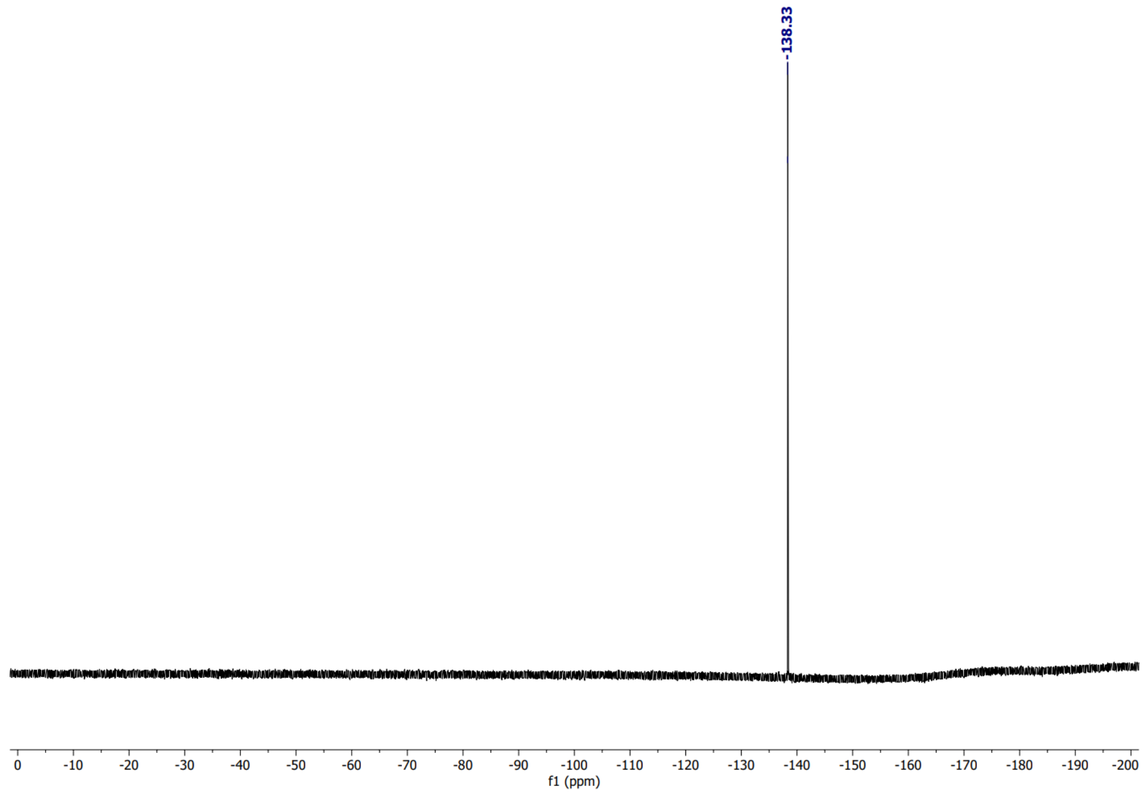

Figure S28. ^{19}F NMR (377 MHz, DMSO-d_6) spectrum of **2**.

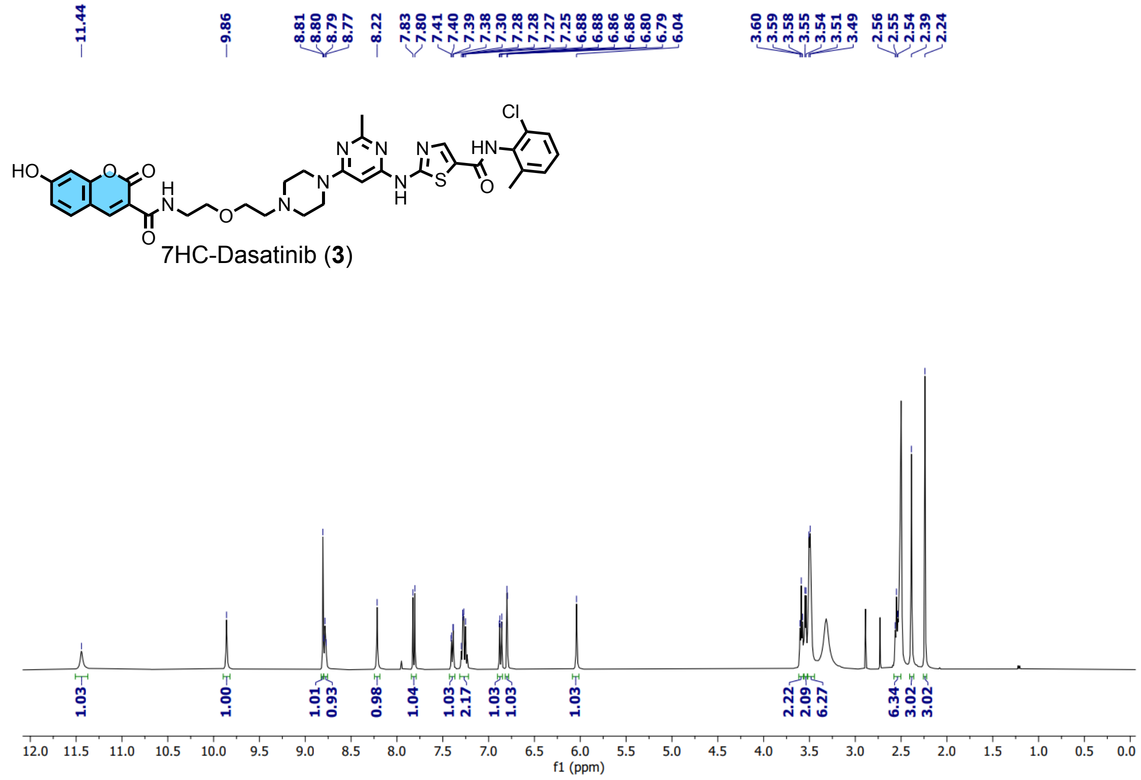

Figure S29. ^{1}H NMR (700 MHz, DMSO-d_6) spectrum of **3**.

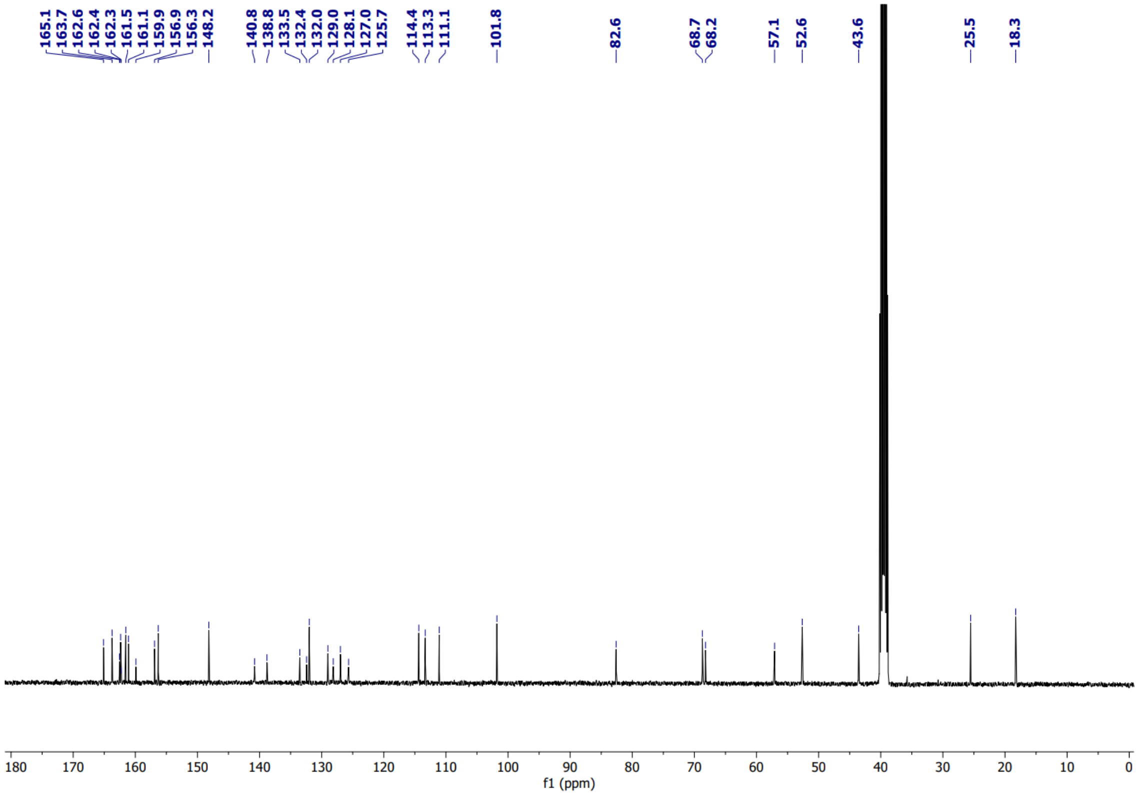

Figure S30. ^{13}C NMR (175 MHz, DMSO-d_6) spectrum of **3**.

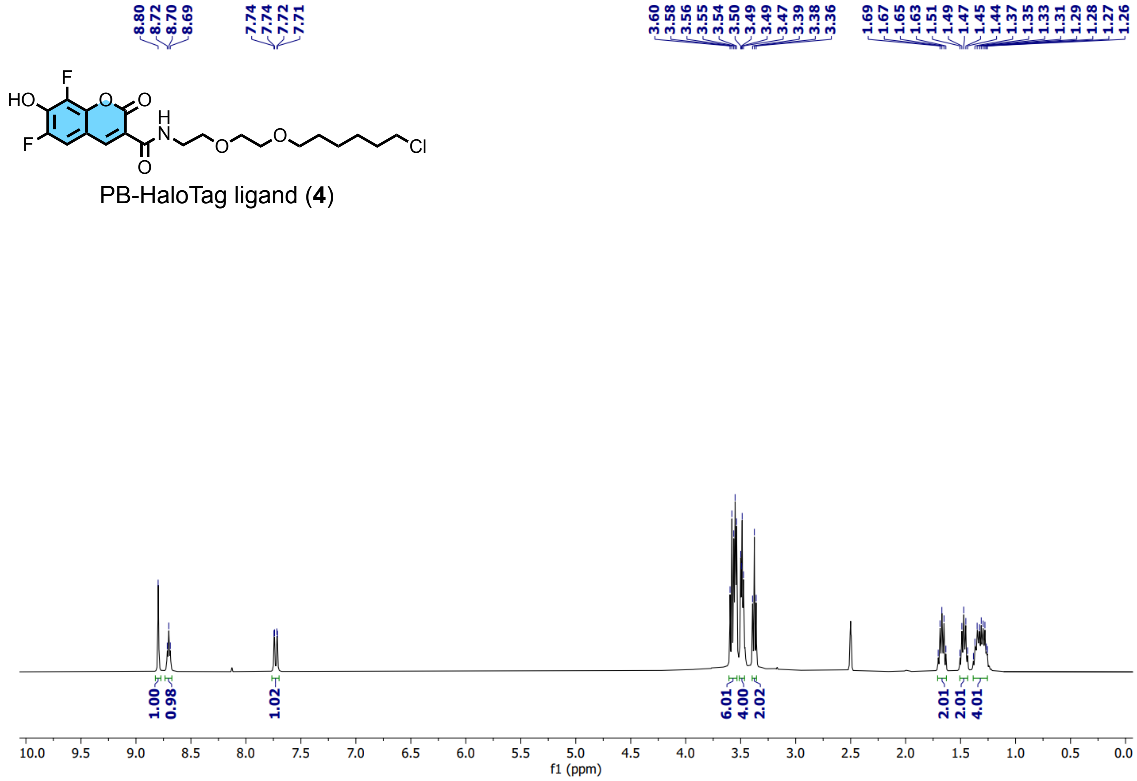

Figure S31. ^{1}H NMR (700 MHz, DMSO-d_6) spectrum of **4**.

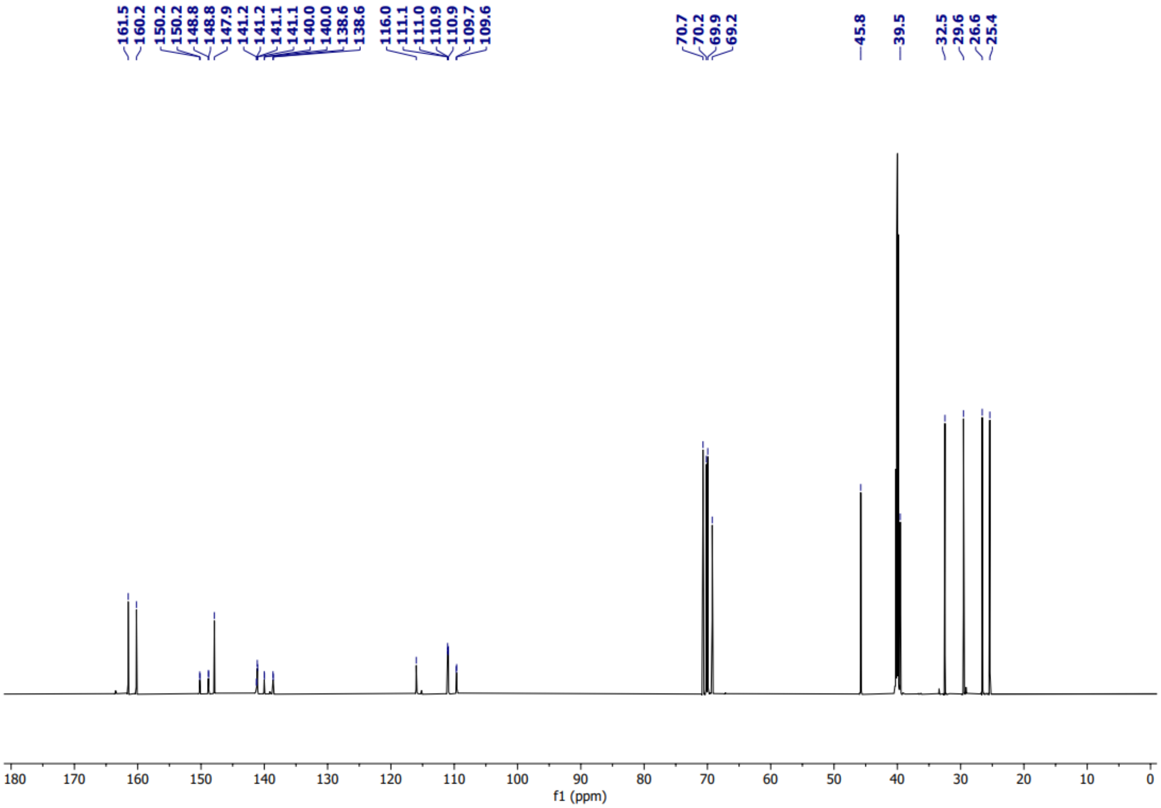

Figure S32. ^{13}C NMR (175 MHz, DMSO-d_6) spectrum of **4**.

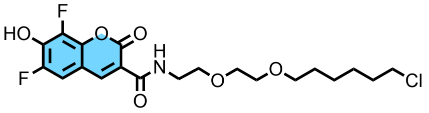

PB-HaloTag ligand (**4**)

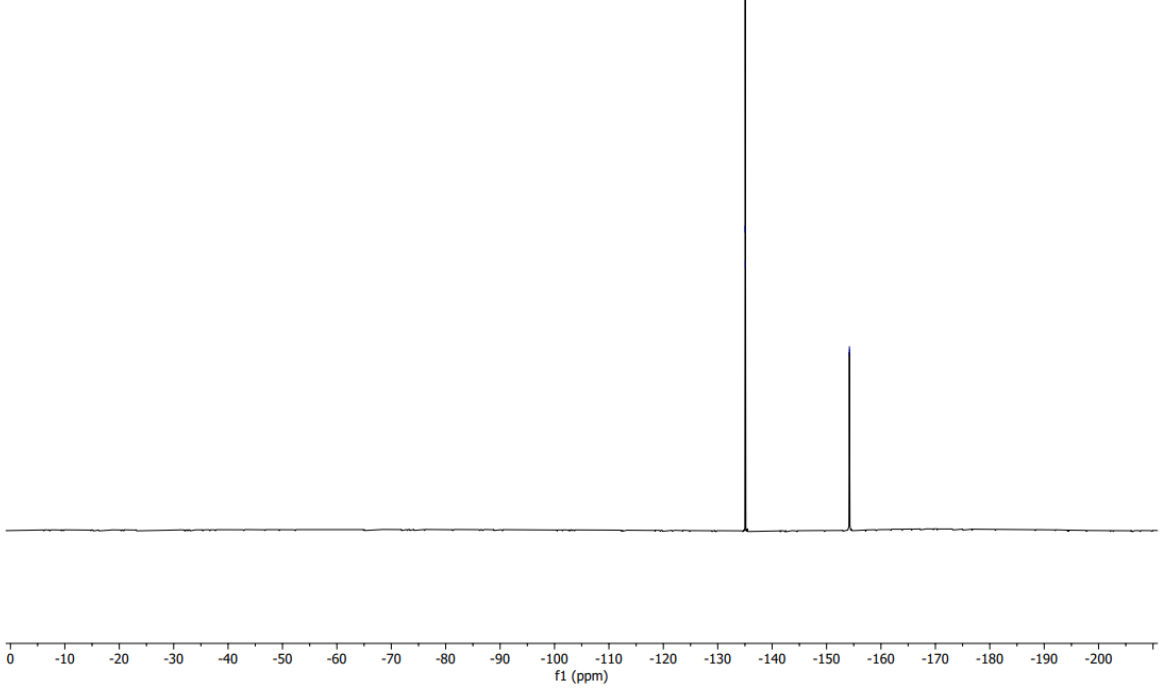

Figure S33. ^{19}F NMR (377 MHz, DMSO-d_6) spectrum of **4**.

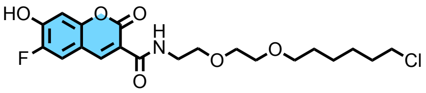

6FC-HaloTag ligand (**5**)

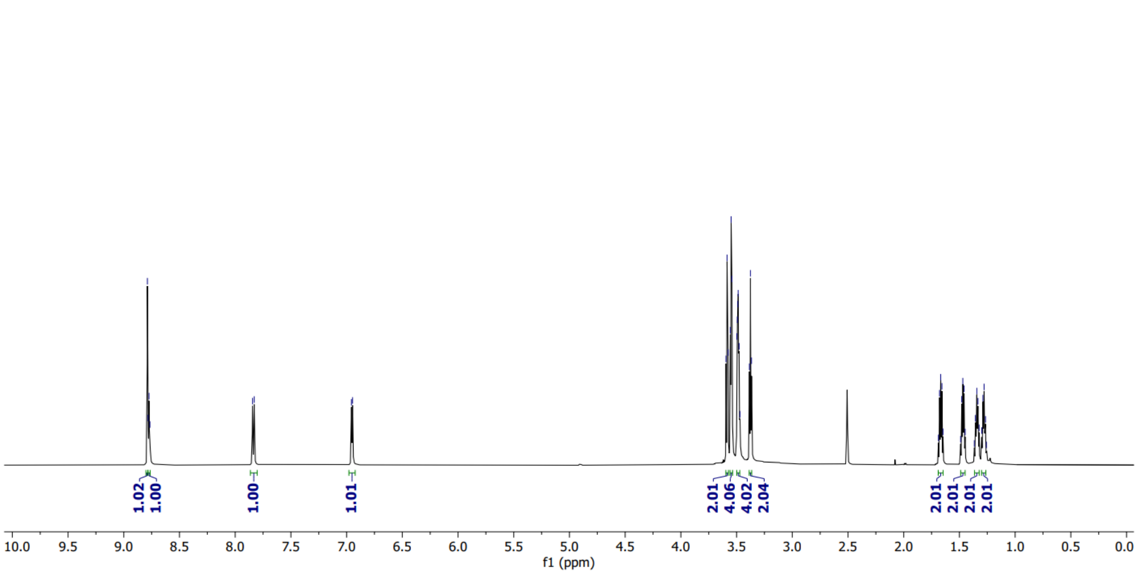

Figure S34 ^{1}H NMR (700 MHz, DMSO-d_6) spectrum of **5**.

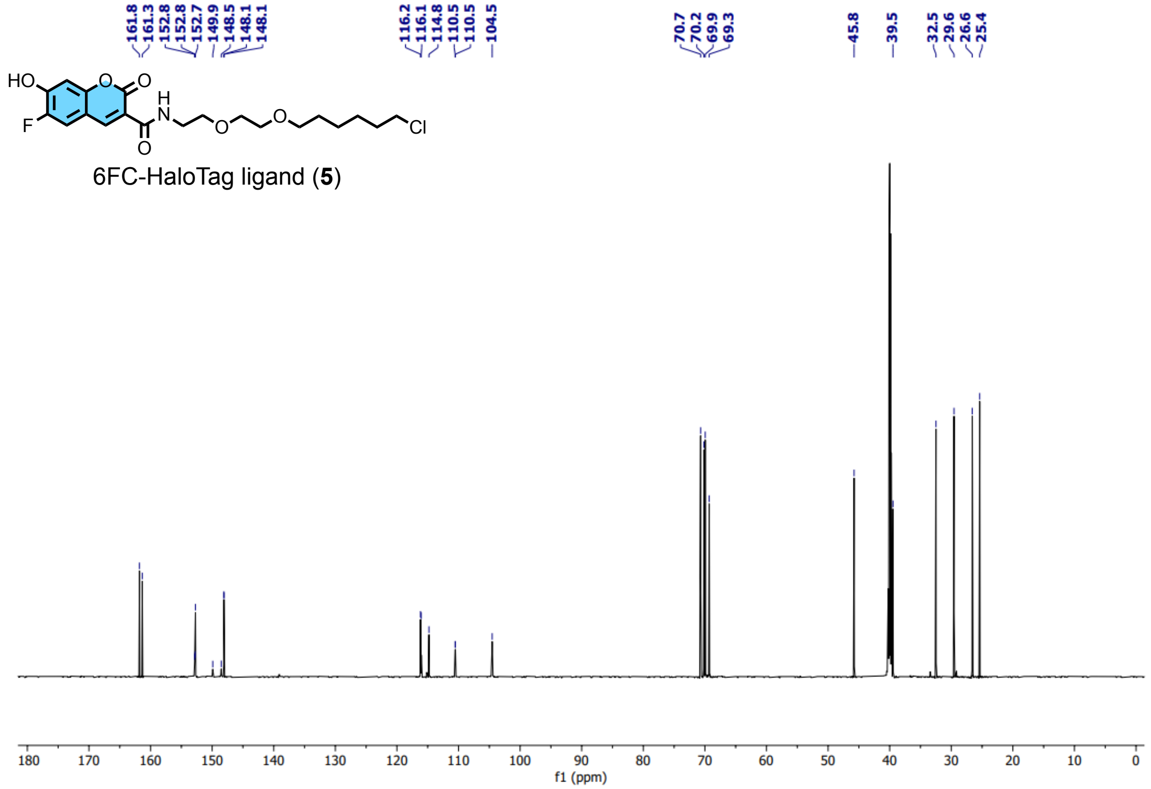

Figure S35. ^{13}C NMR (175 MHz, DMSO-d_6) spectrum of **5**.

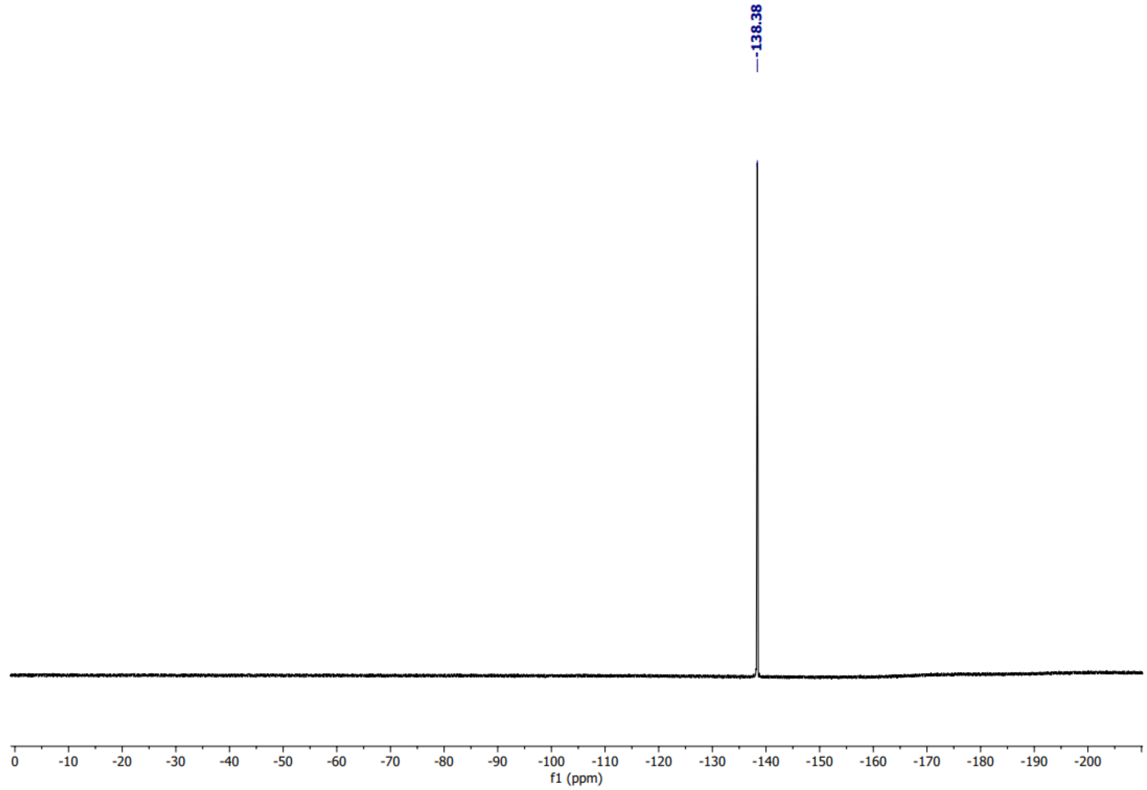

Figure S36. ^{19}F NMR (377 MHz, DMSO-d_6) spectrum of **5**.

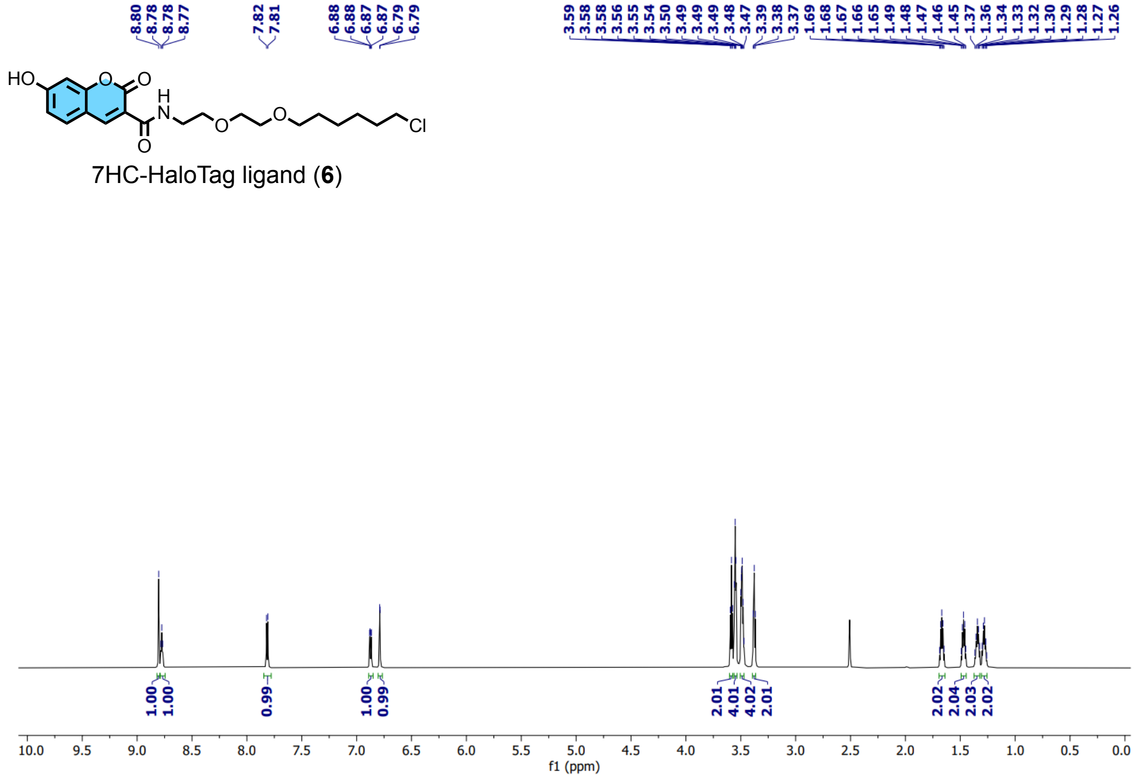

Figure S37. ^{1}H NMR (700 MHz, DMSO-d_6) spectrum of **6**.

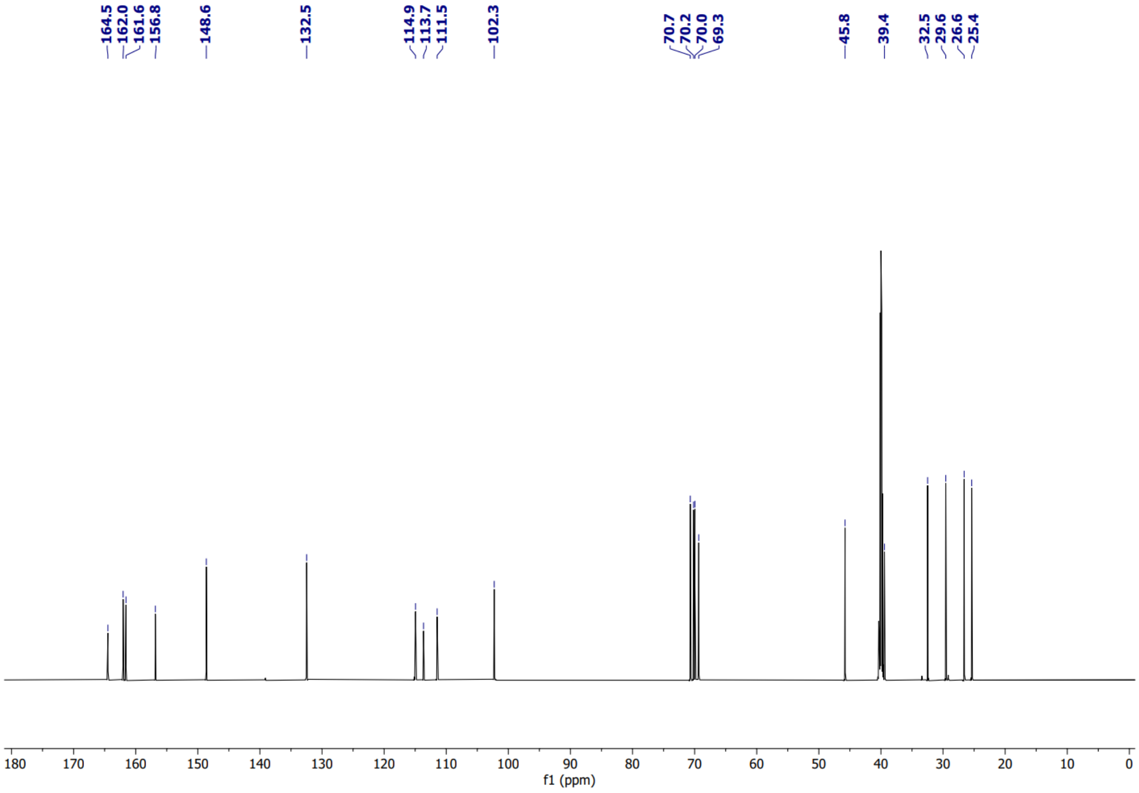

Figure S38. ^{13}C NMR (175 MHz, DMSO-d_6) spectrum of **6**.

Biological Methods

Generation of the HC2 (HEK293T-OATP1B3) cell line. The Gene Editing Shared Resource (GEdSR) and the Drug Discovery Shared Resource HTS lab (DDSR-HTS) of the Ohio State University Comprehensive Cancer Center generated the HC2 cell line. VectorBuilder (https://en.vectorbuilder.com) was used to design and supply a piggyBac transposon vector (pPB-Puro-CMV>hSLCO1B3) containing inverted terminal repeats (ITRs), the hSLCO1B3 (OATP1B3) gene [NM_019844.4] under a CMV promoter, and a puromycin resistance cassette. A hyperactive piggyBac transposase plasmid (pRP[Exp]-mCherry-CMV>hyPBase) was also obtained from VectorBuilder. HEK293T cells (mycoplasma-negative) were seeded in complete DMEM at 0.2 × 10^5 cells per 10 mL (6-well plate). After 24 h, cells were transfected with Lipofectamine 3000 using either pRP[Exp]-mCherry-CMV>hyPBase alone (1.5 µg; control) or pPB-Puro-CMV>hSLCO1B3 plus pRP[Exp]-mCherry-CMV>hyPBase (1.5 µg each; 3 µg total). 48 h later, cells were expanded to 60-mm dishes and selected with 0.5 µg/mL puromycin, with medium changes every 2–3 days, and subsequently expanded to 100-mm dishes. Control cells (transposase only) died within four days, whereas cells transfected with both plasmids survived, confirming puromycin resistance. Puromycin-resistant OATP1B3$^+$ cells were trypsinized, and single clones were isolated by DDSR-HTS staff using a Sony MA900 cell sorter. Cells expressing high OATP1B3 levels were labeled with PB-Gly-Taxol (0.5 µM, 2 h, 37 °C), and single cells in the top 5% of the Pacific Blue–positive population (Ex 405 nm) were plated. Expanded clones were re-tested with PB-Gly-Taxol (0.5 µM, 2 h, 37 °C) and analyzed by flow cytometry. One clone, designated HC2, exhibited ~40-fold higher median blue fluorescence relative to parental HEK293T due to OATP1B3-mediated PB-Gly-Taxol uptake. HC2 cells were morphologically indistinguishable from HEK293T (Figure S39).

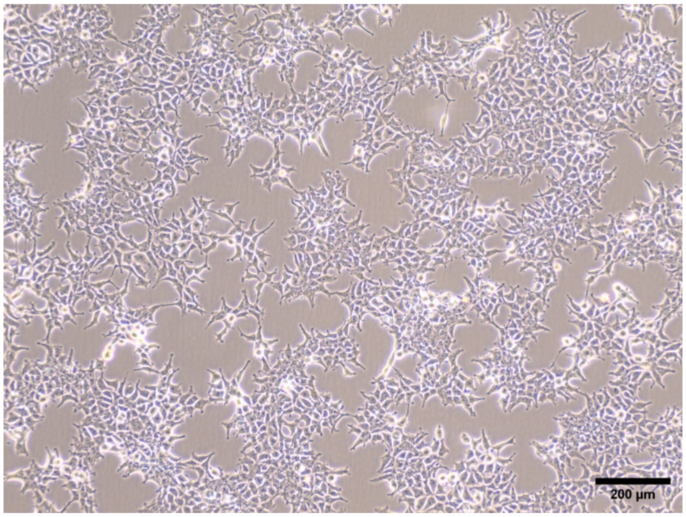

Figure S39. Brightfield micrograph showing the morphology of the HC2 (HEK293T-OATP1B3) cell line. Image acquired with an EVOS XL Core Imaging System (Invitrogen, 10X magnification).

Generation of expression vectors. To coexpress native full-length human kinases with mVenus as a separate marker protein, the previously reported[7] pCMV-IRES-mVenus vector was used. Most full-length kinase cDNAs, including the native stop codon, were purchased in pCMV-IRES-mVenus from TWIST Biosciences. The constructs SRC-IRES-mVenus, DDR1-IRES-mVenus, and mVenus-ABL1b were constructed using In-Fusion cloning. NCBI reference numbers for all kinases investigated are shown in Table 1. For PCR amplification of genes used for In-Fusion cloning, primers were designed to include a 15-bp sequence homologous to the pCMV-IRES-mVenus vector, which had been linearized with BamHI and EcoRI. A Kozak consensus sequence (CCACC) was added immediately upstream of the ATG start codon to ensure efficient translation initiation. The full coding region of each gene, including the stop codon, was cloned into pIRES-mVenus immediately upstream of the IRES site as previously described[7] for Protein Kinase C isozymes. For construction of the DDR1-IRES-mVenus plasmid, DDR1-NanoLuc (Promega, NV2451) was used as the PCR template with the following primers:

DDR1-IRES-F: 5′-CCTTTCGCCAGAATTCTTAAACCGTGTTGAGTGCATCC-3′

DDR1-IRES-R: 5′-TACCGAGCTCGGATCCCCACCATGGGACCAGAGGC-3′

For construction of the SRC-IRES-mVenus plasmid, SRC-NanoLuc (Promega, NV207A) was used as the PCR template with the following primers:

SRC-IRES-F: 5′-TACCGAGCTCGGATCCCCACCATGGGTAGCAACAAGAG-3′

SRC-IRES-R: 5′-CCTTTCGCCAGAATTAGAGGTTCTCCCCGGGCTG-3′

To construct the mVenus-ABL1b expression vector, the nLuc coding sequence in the Promega-nLuc-ABL1b vector (Promega NV1011) was precisely replaced with mVenus using an In-Fusion cloning strategy. The mVenus insert was PCR-amplified from the pCMV-IRES-mVenus plasmid using the primer pair mVenus-F-PromegaV (5'-GGGCTAGCGCTCACCATGGTGAGCAAGGGCGAGG-3') and mVenus-R-PromegaV (5'-ACCACCTGAACCACCCTTGTACAGCTCGTCCATGCC-3'). These primers were designed to introduce 15 bp overlaps homologous to the ends of the linearized vector backbone. The vector backbone was generated by PCR amplification of the Promega-nLuc-ABL1b plasmid using primers PromegaV-F (5'-GGTGGTTCAGGTGGTGGC-3') and PromegaV-R (5'-GGTGAGCGCTAGCCCTATAGTG-3'), thereby excluding the nLuc coding sequence and creating ends compatible with the mVenus insert. PCR products were purified and assembled using the In-Fusion cloning system according to the manufacturer’s instructions, allowing recombination between the overlapping regions of the insert and vector backbone. The resulting construct, mVenus-ABL1b, was transformed into competent *E. coli* cells, and positive clones were verified by whole plasmid DNA sequencing (PlasmidSaurus).

Protein sequence for mVenus-linker-ABL1b in plasmid CMV-mVenus-ABL1b.

MVSKGEELFTGVVPILVELDGDVNGHKFSVSGEGEGDATYGKLTLKLICTTGKLPVPWPTLVTTLGYGLQCF
ARYPDHMKQHDFFKSAMPEGYVQERTIFFKDDGNYKTRAEVKFEGDTLVNRIELKGIDFKEDGNILGHKLEY
NYNSHNVYITADKQKNGIKANFKIRHNIEDGGVQLADHYQQNTPIGDGPVLLPDNHYLSYQSKLSKDPNEKR
DHMVLLEFVTAAGITLGMDELYKGGSGGGGSGGGSSGGAIAMGQQPGKVLGDQRRPSLPALHFIKGAGKKES
SRHGGPHCNVFVEHEALQRPVASDFEPQGLSEAARWNSKENLLAGPSENDPNLFVALYDFVASGDNTLSITK
GEKLRVLGYNHNGEWCEAQTKNGQGWVPSNYITPVNSLEKHSWYHGPVSRNAAEYLLSSGINGSFLVRESES
SPGQRSISLRYEGRVYHYRINTASDGKLYVSSESRFNTLAELVHHHSTVADGLITTLHYPAPKRNKPTVYGV
SPNYDKWEMERTDITMKHKLGGGQYGEVYEGVWKKYSLTVAVKTLKEDTMEVEEFLKEAAVMKEIKHPNLVQ
LLGVCTREPPFYIITEFMTYGNLLDYLRECNRQEVNAVVLLYMATQISSAMEYLEKKNFIHRDLAARNCLVG
ENHLVKVADFGLSRLMTGDTYTAHAGAKFPIKWTAPESLAYNKFSIKSDVWAFGVLLWEIATYGMSPYPGID

```
LSQVYELLEKDYRMERPEGCPEKVYELMRACWQWNPSDRPSFAEIHQAFETMFQESSISDEVEKELGKQGVR
GAVSTLLQAPELPTKTRTSRRAAEHRDTTDVPEMPHSKGQGESDPLDHEPAVSPLLPRKERGPPEGGLNEDE
RLLPKDKKTNLFSALIKKKKKTAPTPPKRSSSFREMDGQPERRGAGEEEGRDISNGALAFTPLDTADPAKSP
KPSNGAGVPNGALRESGGSGFRSPHLWKKSSTLTSSRLATGEEEGGGSSSKRFLRSCSASCVPHGAKDTEWR
SVTLPRDLQSTGRQFDSSTFGGHKSEKPALPRKRAGENRSDQVTRGTVTPPPRLVKKNEEAADEVFKDIMES
SPGSSPPNLTPKPLRRQVTVAPASGLPHKEEAGKGSALGTPAAAEPVTPTSKAGSGAPGGTSKGPAEESRVR
RHKHSSESPGRDKGKLSRLKPAPPPPPAASAGKAGGKPSQSPSQEAAGEAVLGAKTKATSLVDAVNSDAAKP
SQPGEGLKKPVLPATPKPQSAKPSGTPISPAPVPSTLPSASSALAGDQPSSTAFIPLISTRVSLRKTRQPPE
RIASGAITKGVVLDSTEALCLAISRNSEQMASHSAVLEAGKNLYTFCVSYVDSIQQMRNKFAFREAINKLEN
NLRELQICPATAGSGPAATQDFSKLLSSVKEISDIVQRV*
```

Cell Culture. HEK293T cells (ATCC CRL-3216) were obtained from the American Type Culture Collection and verified by STR profiling at The Ohio State University Genomics Shared Resource. This cell line was used for FPCBA because of its high transfection efficiency and capability for high-level protein expression.[8] Cells were routinely tested for mycoplasma contamination (Abcam Mycoplasma PCR detection kit). HEK293T cells and OATP1B3-expressing HEK293T (HC2) cells were cultured in Dulbecco's Modified Eagle Medium (DMEM, high glucose with phenol red; Sigma-Aldrich D6429 or Gibco 11995065) supplemented with 10% fetal bovine serum (FBS), 1% penicillin/streptomycin, and 1% GlutaMAX (Gibco 35050061) to afford complete medium. Cells were maintained at 37 °C in a humidified CO_2 (5%) incubator. Inclusion of phenol red in the culture medium is recommended for FPCBA, as this pH indicator appears to reduce nonspecific cellular binding of some fluorescent probes, potentially by upregulating cellular efflux pathways, as previously described.[9] For cellular binding assays and competition assays, DMEM supplemented with 4% FBS (termed assay medium) was used to reduce ligand depletion. To resuspend adherent cells, culture medium was removed, wells were washed once with PBS (384-well workflow) or medium was removed by aspiration (96-well workflow), and trypsin-EDTA (0.25%, Sigma-Aldrich T4049; 3 mL per T-75 flask) was added. After incubation for 3–5 min at 37 °C, trypsin was quenched by adding fresh complete medium at twice the volume of trypsin used. Cells were pelleted by centrifugation (2000 rpm, 2 min), the supernatant was removed, and cells were resuspended in fresh medium.

Transient Transfection of HEK293T Cells. Transient transfection was performed using the PolyJet In Vitro DNA Transfection Reagent (SignaGen Laboratories, #SL100688). HEK293T or HC2 cells

were seeded in 6-well treated plates at approximately 0.5–0.7 × 10^6 cells/well in 2 mL of complete medium and incubated for 16–24 h at 37 °C (5% CO_2) to promote adhesion. On the day of transfection, plasmid DNA encoding the kinase of interest in a pCMV-IRES-mVenus bicistronic vector (1.0–2.0 μg per well) and PolyJet reagent were combined in a 1:3 ratio (μg DNA : μL PolyJet) in 100 μL of serum-free DMEM per well. The mixture was gently pipetted to mix and incubated at 22 °C for 15 min to allow complex formation. The transfection mixture was then added dropwise to each well, the plate was gently swirled to ensure even distribution, and cells were incubated for 24 h at 37 °C (5% CO_2) prior to assay. For 384-well plate assays conducted at the OSUCCC Drug Discovery Shared Resource High Throughput Screening (DDSR-HTS) laboratory, transfection efficiency was assessed 24 h post-transfection using a Tecan Spark Cyto 600 multimode microplate reader to quantify mVenus fluorescence; typical transfection efficiencies were 50–70%. For 96-well plate assays conducted in the Peterson laboratory, transfection efficiency and cell density were evaluated by flow cytometry analysis of a 1:4 dilution of resuspended cells prior to assay setup.

Fluorescent Probe Cellular Binding Assays (FPCBA). To quantify binding of small molecules to untagged, full-length kinases in living cells, the FPCBA method was used as previously reported.[7] The fluorescent probe 6FC-dasatinib (**2**; $\varepsilon_{413\ nm}$ = 37,000 M^{-1} cm^{-1} in PBS/DMSO 9:1, pH 7.4) was freshly prepared as a 10 mM stock solution in DMSO from dry powder and the concentration was normalized by absorbance using a CLARIOstar Plus (BMG LABTECH) multimode plate reader. PB-dasatinib (**1**; $\varepsilon_{413\ nm}$ = 29,000 M^{-1} cm^{-1} in PBS/DMSO 9:1, pH 7.4) and 7HC-dasatinib (**3**; $\varepsilon_{405\ nm}$ = 22,000 M^{-1} cm^{-1} in PBS/DMSO 9:1, pH 10 to fully deprotonate the phenol) were similarly normalized. Competitor compounds dasatinib and imatinib were prepared as 10 mM stock solutions in DMSO by mass and molecular weight. All assays were incubated at 37 °C, 5% CO_2 for 2 h to allow equilibration prior to analysis by flow cytometry without washing. Living cells were gated by light scatter (FSC-A vs. SSC-A). The bimodal mVenus fluorescence distribution arising from the CMV-IRES-mVenus bicistronic construct was used to distinguish transiently transfected cells (top 20% of the

FITC/mVenus channel population) from non-transfected cells (bottom 20%), which served as an internal nonspecific-binding control. Median fluorescence intensity in the probe detection channel (Pacific Blue, PB450) was plotted as a function of probe or competitor concentration for the transfected and non-transfected populations.

Data Analysis for FPCBA. Probe saturation binding and competitive displacement were analyzed using the FPCBA Database, a custom web application for FPCBA data management, curve fitting, and figure generation (Figures S5–S8 were generated using this application and Adobe Illustrator 2026). All analyses are fully reproducible using GraphPad Prism 11 with the models, constraints, and confidence interval settings described below.

Calculation of probe saturation binding. Fluorescence intensity data from Total and Nonspecific (NS) wells were fit simultaneously to a global four-parameter model equivalent to the One Site – Total and Nonspecific Binding model of GraphPad Prism:

Total: $Y = B_{max} \cdot X/(K_d + X) + NS \cdot X + \text{Background}$

NS: $Y = NS \cdot X + \text{Background}$

where B_{max}, K_d, NS, and Background were shared global parameters optimized jointly across all wells by nonlinear least-squares regression (Levenberg–Marquardt algorithm), minimizing the sum of squared residuals (SSQ) between observed and predicted fluorescence. 95% confidence intervals were computed by the profile likelihood method using the threshold:

$$SSQ_{threshold} = SSQ_{min} \cdot (1 + F(1, df, 0.05) / df)$$

where df = $n - p$ is the degrees of freedom, n is the total number of non-excluded replicate observations across both Total and NS datasets combined, and $p = 4$ is the number of free parameters (B_{max}, K_d, NS, and Background), with the remaining three parameters re-optimized at each step of the profile sweep. Data points above K_d where nonspecific fluorescence dominated the specific signal, or where probe-associated biological activity caused deviation from the binding model, were excluded prior to fitting. Across the 62 probe saturation experiments, n ranged from 20

to 36 non-excluded replicate observations (df = 16–32), with 8–28 points excluded per experiment, and the profile likelihood threshold factor ($1 + F_{crit}/\mathrm{d}f$) ranged from 1.130 to 1.281.

Calculation of signal-to-background ratios. The maximum signal-to-background ratio (S/B) reported for each probe saturation experiment was calculated as:

$$\mathbf{S/B(\mathit{x}) = (T(\mathit{x}) - T_0) / (NS(\mathit{x}) - NS_0)}$$

where T(x) and NS(x) are the mean fluorescence intensities of the Total and Nonspecific wells at probe concentration x, and T_0 and NS_0 are the corresponding means at 0 nM probe. Baseline subtraction removes cellular autofluorescence and probe-independent background common to both well types. Max S/B is the maximum of S/B(x) across all non-excluded concentrations at which the background-subtracted NS denominator ($NS(x) - NS_0$) is positive.

At probe concentrations well below K_d, the NS fluorescence may be indistinguishable from background, causing the denominator to approach zero and S/B to be spuriously inflated (a denominator-collapse artifact). Entries were flagged when the denominator at the concentration contributing Max S/B was less than 10% of the maximum denominator observed across the experiment; for flagged entries, a corrected Max S/B was calculated by restricting the maximization to concentrations meeting this 10% threshold. The corrected value is reported in Table S1 and labeled "S/B (corr.)" in Figures S5–S8. Corrected S/B ≥ 3 was considered adequate for reliable K_i determination. Values of 2–3 should be interpreted with caution, as reduced dynamic range compresses the competition curve and can underestimate K_i via the Cheng–Prusoff equation, and values < 2 may be unreliable without additional supporting evidence.

Calculation of IC_{50} and K_i values. IC_{50} values were calculated from competitor dose–response data fit to a three-parameter sigmoidal model with Hill slope fixed at unity, equivalent to the log(inhibitor) vs. response (three parameters) model of GraphPad Prism:

$$\mathbf{Y = Bottom + (Top - Bottom) / (1 + 10\text{^}(\log IC_{50} - \log[competitor]))}$$

Top was fitted as a shared parameter across replicates. Bottom was fixed to the nonspecific binding value (NS · [probe] + Background, evaluated at the probe concentration used in that experiment) when the saturation fit provided a well-constrained NS baseline. Otherwise, primarily for ABL family kinases at high probe concentrations and selected imatinib experiments, Bottom was fitted as a free parameter, yielding p = 2 (Top and logIC_{50}; 45 of 62 experiments) or p = 3 (Top, Bottom, and logIC_{50}; 17 of 62 experiments). Profile likelihood 95% CIs for logIC_{50} used df = n − p with the appropriate p for each entry. n ranged from 20 to 24 (df = 17–22). When the curve did not reach 50% inhibition within the tested range, IC_{50} and K_i are reported as ">10,000 nM."

Competitor K_i values were calculated from IC_{50} using the Cheng–Prusoff equation:

$\mathbf{K_i = IC_{50} / (1 + [L]/K_d)}$, where [L] is the fixed probe concentration used in that experiment (0.5–500 nM depending on kinase and cell line; see Table S1) and K_d is the cellular K_d from the corresponding saturation experiment. K_i 95% CIs were obtained by applying the same transformation to the lower and upper profile likelihood IC_{50} bounds.

Validation of FPCBA Database calculations. The FPCBA Database was validated by direct comparison with GraphPad Prism 11 across all 62 probe saturation binding and 62 competitive displacement experiments (including ABL1 isoform replicates across probes **1**–**3**, both cell lines, and both competitors) using the profile likelihood CI method described above. Best-fit K_d, IC_{50}, and K_i estimates agreed with Prism 11 within <1% in all cases; K_d and B_{max} CIs converged in all 62 entries and matched Prism 11 output within rounding (± 1 nM). All 62 saturation fits yielded $R^2 \geq 0.95$ (range 0.95–1.00). For competitive displacement, IC_{50} and K_i CIs converged in all 42 experiments where binding was detected (IC_{50} < 5,000 nM, R^2 = 0.76–1.00); CI bounds did not converge for the 20 imatinib entries with IC_{50} > 10,000 nM, consistent with no detectable competition, and these are reported as ">10,000 nM" in Tables 2 and S1.

FPCBA in 96-Well Plate Assay Format (Beckman Coulter CytoFLEX S). Following transfection on 6-well plates, cells were detached by trypsinization (1 mL/well, 37 °C, 3–5 min), quenched with 2

mL/well assay medium (4% FBS), resuspended with a serological pipet, transferred to 15 mL conical tubes, and pelleted by centrifugation (2000 rpm, 2 min). After removal of the supernatant by vacuum aspiration, cells were resuspended in assay medium (1 mL) and a 1:4 dilution was analyzed by flow cytometry to assess transfection efficiency and cell density. Cell density was adjusted to ~500K cells/mL in assay medium. For assay setup, 2× stock solutions of cells in suspension (100 µL) were combined with 2× stock solutions of compounds (100 µL) on CytoOne non-treated 96-well plates (USA Scientific, cat. #5665-5185) in duplicate.

For saturation binding assays, 10-point dose–response curves were generated by 2-fold serial dilution; the highest final probe concentration was 10 µM (HEK293T cells) or 1 µM (HC2 cells). Serial dilutions of 1000× probe stocks in DMSO were prepared in 1.5 mL microcentrifuge tubes to span 10 µM to 39 nM (HEK293T) or 1 µM to 3.9 nM (HC2) (dilutions 1–9), with a DMSO-only vehicle control (1% final DMSO, 0 nM probe; dilution 10). On 96-well assay plates, 0.70 µL of each 1000× stock was transferred into 6.3 µL DMSO to provide a 100× intermediate solution, which was then diluted 1:50 into 350 µL assay medium. For competition assays, dasatinib or imatinib stocks (10 mM in DMSO) were serially diluted 3-fold in DMSO across 11 points (dilutions 1–11; 10 µM to 0.17 nM or 1 µM to 0.017 nM final), with a DMSO-only control (1% final [DMSO]; dilution 12). A 1000× stock of 6FC-dasatinib at the fixed concentration shown in Table S1 was prepared separately and combined with the competitor dilutions as described above.

Cells were analyzed using a Beckman Coulter CytoFLEX S flow cytometer (configuration: B2-R0-V2-Y2) equipped with 405 nm and 488 nm diode lasers. Emitted photons were collected through 450/45 BP (Pacific Blue, probe channel) and 525/40 BP (mVenus, expression reporter) filters. Cells were analyzed for 30 s per well at fast flow speed. Instrument settings: FSC threshold = 500,000; mixing and backflush times = 3 s; gain settings: FITC = 20, PB450 = 20. K_d, K_i, and IC_{50} values were calculated from two technical replicates and confirmed in at least one independent biological replicate.

FPCBA in 384-Well Plate Assay Format (Sartorius iQue3). Following transfection on 6-well plates, cells were resuspended by trypsinization and adjusted to a density of 0.25 × 10^6 cells/mL in assay medium. Cells were dispensed into 384-well plates at 50 µL/well using a multiflo liquid handler and incubated at 37 °C (5% CO_2) for equilibration prior to compound addition. Compounds were dispensed using an Acoustic Liquid Handler (Echo 650, Beckman Coulter, cat. #001-16079), with a final DMSO concentration of ≤ 0.3% in all wells. For saturation binding assays, 12-point dose–response curves were generated by 2-fold serial dilution of 6FC-dasatinib (**2**). The highest final probe concentration was 10 µM for HEK293T cells and 1 µM for HC2 cells. For competition assays, dasatinib (highest final concentration: 1 µM) or imatinib (highest final concentration: 10 µM) were dispensed as 12-point, 3-fold serial dilutions in the presence of a fixed concentration of 6FC-dasatinib (**2**) probe. Plates were shaken for 2 min following compound addition to ensure mixing and then incubated at 37 °C (5% CO_2) for 2 h before analysis by flow cytometry. Data acquisition was performed on a Sartorius iQue3 High-Throughput Flow Cytometer. Signal reduction of 70% for FITC and 90% for PB450 was implemented to approximate the sensitivity of the CytoFLEX flow cytometer at the gain settings used and improve linearity of response. Cell singlets were identified and gated based on FSC-A versus FSC-H. The mVenus fluorescence distribution (488 nm excitation; 530/30 nm emission) was used to gate the bimodal transfected (top 20%) and non-transfected (bottom 20%) cell populations as described above. Median PB450 fluorescence values (405 nm excitation; 445/45 nm emission) for each population were plotted against probe or competitor concentration for determination of cellular K_d and K_i values by non-linear regression as previously described.

Confocal microscopy. For confocal microscopy of transfected cells (Figure 5), HEK293T and HC2 cells in complete DMEM medium were seeded onto an 8-well cover glass slide (Ibidi, #80826-90, 200 µL, 100K cells/mL) and incubated (37 °C, 5% CO_2) for 16 h to promote adhesion. After 16 h, 180 µL of the cell culture medium was carefully removed from the well and replaced with the same volume of PB-Gly-Taxol (0.5 µM with or without 100 µM of verapamil in HEK293T or 0.1 µM in HC2,

1% DMSO). Cells were treated for 2 h at 37 °C (5% CO2) before imaging with an inverted Leica TCS SP8 confocal laser-scanning microscope (63X oil-immersion objective). Fluorescent probes were excited at 405 nm with emitted photons collected from 425–500 nm. Laser power and PMT gain settings were identical for all images within a given experiment for accurate comparisons of cellular fluorescence.

Optical spectroscopy. Emission spectra of fluorescent probes were obtained using a CLARIOstar Plus multimode plate reader (gain = 1000, focal height = 8 mm) in black 96-well plates (Greiner Bio-One, item #: 655076). Probes **1**–**6** were prepared in PBS (pH 7.4, 1% DMSO) or other solutions at 500 nM in a final volume of 150 µL per well. For probes **4**–**6**, recombinant HaloTag standard protein (50 µM, Promega, catalog #: G4491) was added to relevant wells at a final concentration of 1 µM to evaluate the effects of protein complexation on fluorescence. To examine higher concentrations of protein, FBS was introduced at a final concentration of 50% (pH = 7.3). Following addition of HaloTag protein and/or FBS, plates were shaken at room temperature for 1 h at 300 rpm before measurement. Absorbance spectra of probes in PBS (pH 7.4) and coumarin 30 (**C30**) in acetonitrile were acquired at 10 µM (1% DMSO) in clear 96-well plates (Greiner Bio-One, item #: 655801). The molar extinction coefficient of **C30** in acetonitrile was determined by plotting absorbance versus concentration. Absorbance spectra were measured at 1 nm intervals between 220-1000 nm. Fluorescence emission spectra were recorded at 1 nm intervals (8 nm bandwidth) at 500 nM using excitation at 405 nm (8 nm bandwidth). Area under the curve values from 425–650 nm and 428–473 nm were calculated using rfu values with GraphPad Prism. Spectra were normalized to λ_{max} of **C30** for comparison. The 428–473 nm region corresponds to the 450/45 nm CytoFLEX violet-channel bandpass filter used for flow cytometry. Addition of 100% FBS to samples to provide a final concentration of 50% FBS reduced the concentrations of HaloTag ligands and HaloTag protein by 2-fold, and these emission spectra were multiplied by 2 to allow comparison with undiluted samples.

NanoBRET assays of Nluc-ABL1 (Promega cat# NV1011). HEK293T and HC2 cells were maintained in complete growth medium consisting of DMEM supplemented with 10% FBS at 37 °C in a humidified 5% CO_2 incubator. For transfection, cells were detached using 0.25% trypsin-EDTA, resuspended in complete medium, and adjusted to a final density of 2 × 10^5 cells/mL. Lipid:DNA complexes were prepared at a 1:3 DNA-to-PolyJet ratio by combining 900 ng of carrier DNA with 100 ng NanoLuc–ABL1 (NLuc-ABL1) fusion vector (ABL1b) in Opti-MEM, followed by addition of PolyJet transfection reagent. Complexes were incubated for 15 min at room temperature before mixing with the cell suspension and dispensing into 6-well plates for 24 h expression of NLuc-ABL1. NanoBRET assays were performed using the NanoBRET Nonbinding Surface (NBS) format to quantify intracellular binding of BODIPY-Dasatinib (**8**, Promega Tracer K4) to NLuc-ABL1 in intact cells. HEK293T and HC2 cells were harvested 24 h post-transfection, resuspended in assay medium (Opti-MEM with 4% FBS), and diluted to 2.5 × 10^5 cells/mL. A 20X tracer stock was prepared and serially diluted 2-fold across 11 concentrations. For each condition, 5 µL tracer dilution was added to 95 µL of cell suspension per well in white 96-well NBS plates. To determine nonspecific binding, a final concentration of 10 µM unlabeled dasatinib was included in parallel wells. Plates were incubated for 2 h at 37 °C before adding 50 µL of 3X Nano-Glo Substrate/Inhibitor solution. After a 2 min room temperature incubation, donor (450 nm) and acceptor (610 nm) emission values were measured on a CLARIOstar Plus multimode plate reader. BRET ratios were converted to milliBRET units (mBU). Tracer binding curves were fitted in GraphPad Prism using a one-site total and nonspecific binding model to determine the apparent tracer affinity (cellular K_d). Competitive NanoBRET assays were performed to quantify intracellular binding affinities (cellular K_i) of dasatinib and imatinib for NLuc-ABL1. Transfected HEK293T and HC2 cells were prepared as described above and adjusted to 2.5 × 10^5 cells/mL in assay medium. BODIPY-Dasatinib (**8**) was added to achieve final concentrations of 12.5 nM (HC2) or 50 nM (HEK293T). Test compounds were prepared as 10 mM stocks in DMSO and serially diluted 3-fold across 11 concentrations, then diluted to 10X

in assay medium. Each well received 85 µL cells with tracer plus 10 µL of diluted compound. Background wells lacking tracer were prepared similarly. Plates were mixed for 2 min at 900 rpm and incubated for 2 h at 37 °C. After addition of 3X NanoBRET substrate/inhibitor solution and a 2 min incubation, luminescence was recorded as above. mBU values were plotted against log compound concentration. Cellular IC_{50} values were obtained using a log(inhibitor) vs. response (three parameters) model, and cellular K_i values were calculated using the one site Fit K_i model of GraphPad Prism 11.

Cytotoxicity assays. HEK293T and HC2 cells in complete DMEM medium were seeded in a white flat-bottom 96-well plate (Corning, #3917, 150 µL, 30,000 cells/well). The seeded cells were treated with probes **1**–**3**, dasatinib, imatinib, or the cytotoxic control compound staurosporine (0.5% DMSO) and incubated (37 °C, 5% CO_2) for 24 h. Control wells containing only complete DMEM were used to determine background luminescence. The CellTiter-Glo 2.0 Reagent (Promega, #G9242, 50 µL/well, pre-equilibrated at room temperature) was added and the contents were mixed for 2 min using an orbital shaker (Labnet, 500 RPM). The plate was incubated at room temperature in dark for 10 min. Luminescence was recorded on a CLARIOstar Plus multimode plate reader (λ_{em} = 555 nm, 80 nm bandpass filter, gain = 2500). Cytotoxicity was measured by luminescence using a log(inhibitor) vs. response (three parameters) model (GraphPad Prism 11).

Analysis of the kinetics of probe uptake. Kinetics of uptake of probes **1**–**3** in living HEK293T and HC2 cells transiently transfected with ABL1a-IRES-mVenus (Figure S2) were measured by confocal microscopy essentially as previously described.[7] HEK293T cells were harvested with 0.25% trypsin–EDTA, resuspended in complete DMEM, and plated at 5000 cells/well (200 µL) in black, tissue-culture–treated 96-well PhenoPlates (Revvity/PerkinElmer). Plates were incubated overnight at 37 °C with 5% CO_2. The next day, cells were transiently transfected with ABL1a-IRES-mVenus using PolyJet by mixing plasmid DNA (1.0 µg) and PolyJet (3 µL) in unsupplemented DMEM (100 µL), incubating for complex formation, and adding 20 µL to each well. These conditions were chosen to

avoid the reduced viability observed at higher DNA:cell ratios. Cells were incubated 24 h before imaging. Probe solutions were prepared fresh from dry powders, and concentrations were normalized by absorbance spectroscopy as described previously. A 2 mM DMSO stock of 7HC-Dasatinib was diluted into DMEM containing 4% FBS to generate a 2 µM intermediate solution; 6FC- and PB-Dasatinib DMSO stocks (1 mM) were similarly diluted to 2 µM. For kinetic assays, 2 × probe solutions (100 µL) were added 1:1 to the existing well volume (100 µL) to achieve 1 µM final probe concentration and 1% DMSO. Experiments were performed in wells E7–E12 of transfected plates. Uptake kinetics were collected on an Opera Phenix Plus high-content confocal imaging system using the on-board liquid handler for rapid probe addition. Cells were maintained at 37 °C and 5% CO_2 in the humidified chamber of the Opera Phenix. Time-lapse confocal and widefield images were acquired at 1-min intervals with a 20 × water-immersion objective. Quantitative analysis was restricted to highly transfected cells (50th–75th percentile of mVenus fluorescence). Pacific Blue fluorescence (contrast) in these cells was quantified over time using PerkinElmer eHarmony 5.1 PhenoLOGIC, background-subtracted, and normalized to 100% of the brightest probe. Data from duplicate experiments were fit to a one-phase association model in GraphPad Prism to obtain probe accumulation half-times ($t_{1/2}$).

Calculation of median total intracellular probe concentrations by flow cytometry using calibration beads. NIST-standardized Ultra Rainbow Fluorescent Beads (Spherotech, URQP-38-6K) were used to quantify intracellular concentrations of probes **1**–**3** for analysis of cellular uptake by qFlow. Equivalent Reference Fluorophore (ERF) values for coumarin 30 (**C30**, Ex 405 nm, Em 450/45 nm) on beads corresponding to five PB450 intensities (52,400; 323,000; 995,000; 3,830,000; 7,170,000, provided by the manufacturer) were plotted against median PB450-A signals to generate standard curves (Figure S4). Using this PB450 standard curve on a CytoFLEX cytometer (gain = 20), the median intracellular concentration of coumarin fluorophores per cell (Y)

for non-transfected cells (bottom 20% of bimodal distributions in competition assays) was calculated as:

Y = median [intracellular fluorophores]

$$= (65.46 \times (X/Z) + 152860) / ((6.022 \times 10^{23}\ \text{mol}^{-1})(1.7 \times 10^{-12}\ \text{L})) - C$$

where:

X = median PB450 value for the bottom 20% of non-transfected live cells treated with probes **1**–**3**

Z = probe-specific correction factor relative to **C30** (1.4 for PB-Dasatinib (**1**), 2.2 for 6FC-Dasatinib (**2**), 1.2 for 7HC-Dasatinib (**3**); Table 1; see calculation method below)

C = background fluorescence correction, defined as Y when X equals background fluorescence (~2000 rfu; e.g., at [probe] = 0 nM during cellular K_d determinations), ensuring Y = 0 at background levels of fluorescence in the absence of added probe. Correction factors (Z) reflect the fraction of coumarin 30 emission captured through the 450/45 filter. For the CytoFLEX flow cytometer, the slope (65.46) and Y intercept (152,860) from calibration with coumarin 30-equivalent beads (Figure S4A) were used. For analysis on the iQue3 flow cytometer, the slope (38.72) and Y-intercept (161098) values shown in Figure S4B (90% signal reduction for PB450) were used. For probes **1**–**3** in FBS (1% DMSO, 500 nM), Z = $AUC_{(428–473)}$ (probe) / ($AUC_{(428–473)}$ (**C30**)). Cell volume (1.41–1.95 × 10^{-12} L; average 1.7 × 10^{-12} L) was based on reported[10, 11] spherical diameters of HEK293 cells (13.9–15.5 µm).

__**Estimation of concentrations of overexpressed kinases in living cells by flow cytometry.**__ For kinases overexpressed using pCMV-IRES-mVenus vectors, cellular concentrations in the top 20% of transfected cells were estimated using B_{max} values from probe binding and **C30** bead standards. For the CytoFLEX flow cytometer, the concentration of native kinase per cell (molar) was calculated as:

Median [kinase] per cell $= (65.46 \times B_{max}/Z + 152860) / ((6.022 \times 10^{23}\ \text{mol}^{-1})(1.7 \times 10^{-12}\ \text{L}))$

with the same definitions for Z, slope, and intercept as above. B_{max} values for binding of probes to ABL1 are shown in Table S1. For data obtained with the iQue3 flow cytometer, the slope and Y-intercept shown in Figure S4B (90% signal reduction for PB450) were used.

The median effective bulk concentration of expressed kinase (the equivalent concentration if the intracellular kinase were distributed throughout the well volume) was calculated as:

Bulk [kinase] = (median [intracellular kinase] per cell × (1.7 × 10^{-12} L) × (2.5 × 10^{8} cells/L), where 250,000 cells/mL were added to a 96-well (or 384-well) plate.

High-level overexpression of kinases such as ABL1 (e.g., from pCMV-IRES-mVenus in HEK293T cells) typically produces 5–10 µM ABL1 in the top 20% of cells (e.g., B_{max} = 270,000 rfu for probe **2** at PB450 gain = 20). The total bulk [kinase] was estimated based on these assumptions: bimodal transfection (50% express, 50% don't express); top 20% are at full expression, remaining 30% at 50% expression; 250,000 cells/mL. This provided the total bulk expressed kinase concentrations listed in Table S1 (0.19–5.8 nM). For most competition assays, the probe concentration substantially exceeded the estimated bulk kinase concentration (Table S1), making ligand depletion negligible. For HC2 competition assays where the applied probe concentration (0.5 nM) was comparable to the estimated bulk kinase concentration (0.72–0.96 nM for ABL1a), some probe depletion by kinase binding cannot be excluded; however, because the cellular K_d values used in the Cheng–Prusoff equation were measured under identical cell density and probe concentration conditions, any systematic depletion effect is incorporated into the fitted K_d, and the resulting K_i values remain internally self-consistent.

<u>Equilibrium dialysis of rat liver homogenate to model non-specific intracellular protein binding.</u> We partnered with WuXi AppTec to use equilibrium dialysis of rat liver homogenate as an established[12] model of intracellular protein binding of small molecules. Test compounds (dasatinib, imatinib, PB-Dasatinib (**1**), 6FC-Dasatinib (**2**), 7HC-Dasatinib (**3**)) and the control propranolol were assayed at 2 µM. Stock solutions (10 mM in DMSO) were diluted to 400 µM working stocks and

added to liver homogenate to generate matrix-based loading solutions at the target concentration. Stop solutions consisted of acetonitrile containing tolbutamide (250 nM) and labetalol (250 nM). Male Sprague–Dawley rat liver homogenate (WuXi AppTec) was thawed, equilibrated at 37 °C, and diluted 1:5 (v/v) in phosphate-buffered saline (PBS; 100 mM sodium phosphate, 150 mM NaCl, pH 7.4 ± 0.1). Dialysis membranes (12–14 kDa MWCO, HT Dialysis) were preconditioned by sequential soaking in ultrapure water, 20:80 ethanol/water, and water. Equilibrium dialysis was performed in a 96-well HT-Dialysis plate. For time-zero (T_0) samples, loading solutions were mixed 1:1 (v/v) with PBS and immediately quenched with stop solution. For experimental dialysis, 100 µL of loading matrix (donor) and 100 µL PBS (receiver) were added in triplicate. Plates were incubated for 4 h at 37 °C, 5% CO_2, with orbital rotation (~100 rpm). After incubation, donor and receiver samples were collected, matrix and PBS were matched 1:1 (v/v), quenched, vortexed, centrifuged, and supernatants analyzed by LC–MS/MS. Matrix-matched blanks were prepared using untreated homogenate and PBS. Unbound fraction in undiluted liver homogenate was calculated using dilution-corrected equilibrium dialysis:

% Unbound (undiluted) = 100(1/D) / ((1/(F/T) – 1) + 1/D)

where F and T are analyte/internal standard peak-area ratios in receiver and donor chambers after 4 h, T_0 is the corresponding time-zero ratio, and D is the homogenate dilution factor. Percent bound drug was calculated as 100 – % unbound. Recovery was assessed as:

% Recovery = 100(F + T) / T_0.

References for the supporting information

(1) Harris, R. K.; Becker, E. D.; De Menezes, S. M.; Granger, P.; Hoffman, R. E.; Zilm, K. W.; International Union of, P.; Applied Chemistry, P.; Biophysical Chemistry, D. Further conventions for NMR shielding and chemical shifts (IUPAC Recommendations 2008). *Magn. Reson. Chem.* **2008**, *46* (6), 582-598. DOI: 10.1002/mrc.2225

(2) Lee, M. M.; Gao, Z.; Peterson, B. R. Synthesis of a Fluorescent Analogue of Paclitaxel That Selectively Binds Microtubules and Sensitively Detects Efflux by P-Glycoprotein. *Angew. Chem. Int. Ed. Engl.* **2017**, *56* (24), 6927-6931. DOI: 10.1002/anie.201703298

(3) Vasta, J. D.; Corona, C. R.; Wilkinson, J.; Zimprich, C. A.; Hartnett, J. R.; Ingold, M. R.; Zimmerman, K.; Machleidt, T.; Kirkland, T. A.; Huwiler, K. G.; Ohana, R. F.; Slater, M.; Otto, P.; Cong, M.; Wells, C. I.; Berger, B. T.; Hanke, T.; Glas, C.; Ding, K.; Drewry, D. H.; Huber, K. V. M.; Willson, T. M.; Knapp, S.; Muller, S.; Meisenheimer, P. L.; Fan, F.; Wood, K. V.; Robers, M. B. Quantitative, Wide-Spectrum Kinase Profiling in Live Cells for Assessing the Effect of Cellular ATP on Target Engagement. *Cell Chem. Biol.* **2018**, *25* (2), 206-214 e211. DOI: 10.1016/j.chembiol.2017.10.010

(4) Ottis, P.; Toure, M.; Cromm, P. M.; Ko, E.; Gustafson, J. L.; Crews, C. M. Assessing Different E3 Ligases for Small Molecule Induced Protein Ubiquitination and Degradation. *ACS Chem. Biol.* **2017**, *12* (10), 2570-2578. DOI: 10.1021/acschembio.7b00485

(5) Lee, M. M.; Peterson, B. R. Quantification of Small Molecule-Protein Interactions using FRET between Tryptophan and the Pacific Blue Fluorophore. *ACS Omega* **2016**, *1* (6), 1266-1276. DOI: 10.1021/acsomega.6b00356

(6) Rane, D.; Shaik, A. B.; Lee, S.; Hu, X. J.; Peterson, B. R. Synthesis of Monofluorinated 7-Hydroxycoumarin-3-Carboxamides as Cell-Permeable Fluorescent Molecular Probes. *ACS Med. Chem. Lett.* **2024**, *15* (5), 677-683. DOI: 10.1021/acsmedchemlett.4c00085

(7) Yin, Y.; Zhao, S. L.; Rane, D.; Lin, Z.; Wu, M.; Peterson, B. R. Quantification of Binding of Small Molecules to Native Proteins Overexpressed in Living Cells. *J. Am. Chem. Soc.* **2024**, *146* (1), 187-200. DOI: 10.1021/jacs.3c07488

(8) Sullivan, C. S.; Pipas, J. M. T antigens of simian virus 40: molecular chaperones for viral replication and tumorigenesis. *Microbiol. Mol. Biol. Rev.* **2002**, *66* (2), 179-202. DOI: 10.1128/MMBR.66.2.179-202.2002

(9) Hopp, L.; Bunker, C. H. Lipophilic Impurity of Phenol Red Is a Potent Cation-Transport Modulator. *J. Cell. Physiol.* **1993**, *157* (3), 594-602. DOI: DOI 10.1002/jcp.1041570320

(10) Mateus, A.; Matsson, P.; Artursson, P. Rapid measurement of intracellular unbound drug concentrations. *Mol. Pharm.* **2013**, *10* (6), 2467-2478. DOI: 10.1021/mp4000822

(11) Dietmair, S.; Hodson, M. P.; Quek, L. E.; Timmins, N. E.; Gray, P.; Nielsen, L. K. A multi-omics analysis of recombinant protein production in Hek293 cells. *PLoS One* **2012**, *7* (8), e43394. DOI: 10.1371/journal.pone.0043394

(12) Riccardi, K.; Ryu, S.; Lin, J.; Yates, P.; Tess, D.; Li, R.; Singh, D.; Holder, B. R.; Kapinos, B.; Chang, G.; Di, L. Comparison of Species and Cell-Type Differences in Fraction Unbound of Liver Tissues, Hepatocytes, and Cell Lines. *Drug Metab. Dispos.* **2018**, *46* (4), 415-421. DOI: 10.1124/dmd.117.079152
